# The broad‐spectrum antimicrobial peptide BMAP‐27B potentiates carbapenems against NDM‐producing pathogens in food animals

**DOI:** 10.1002/mlf2.70020

**Published:** 2025-06-24

**Authors:** Xiaoxiao Zhang, Yongdong Li, Lei Xu, Zhe Chen, Shengzhi Guo, Jun Liao, Min Ren, Yao Wang, Yi Chen, Chuanxing Wan, Jing Zhang, Xihui Shen

**Affiliations:** ^1^ State Key Laboratory for Crop Stress Resistance and High‐Efficiency Production, Shaanxi Key Laboratory of Agricultural and Environmental Microbiology College of Life Sciences, Northwest A&F University Yangling China; ^2^ Ningbo Municipal Center for Disease Control and Prevention Ningbo China; ^3^ College of Life Science and Technology Tarim University Alar China

**Keywords:** antibacterial mechanism, antibiotic synergist, antimicrobial peptides, carbapenem resistance, NDMs

## Abstract

The emergence and spread of antibiotic‐resistant pathogens in food animals pose a major threat to global public health. Carbapenem‐resistant *Enterobacteriaceae* (CRE), particularly those producing New Delhi Metallo‐β‐lactamase (NDM‐CRE), are prevalent in livestock and have acquired resistance to nearly all commonly used β‐lactam antibiotics. This study evaluated the efficacy of the antimicrobial peptide BMAP‐27B, a derivative of the cathelicidin family, against NDM‐CRE strains in food animals. BMAP‐27B showed potent antibacterial activity and rapid bactericidal effects against CRE, as well as comparable effects against human carbapenem‐resistant *Acinetobacter baumannii*. Furthermore, BMAP‐27B effectively penetrated and cleared biofilms formed by virulent strains of *Escherichia coli* and *Klebsiella pneumoniae*. Mechanistic studies indicated that BMAP‐27B exerts its antibacterial activity by disrupting bacterial membranes and inhibiting bacterial energy metabolism. BMAP‐27B effectively enhances the efficacy of carbapenems against NDM‐positive isolates by inhibiting efflux pump activity and chelating Zn^2+^ to inhibit NDM proteases, thus reversing carbapenem resistance in NDM‐CRE. Importantly, BMAP‐27B maintained excellent antimicrobial stability under extreme pH changes and high salt concentrations, along with resistance to serum and protease degradation. Investigations revealed that BMAP‐27B also shows ideal biocompatibility and therapeutic efficacy in vivo. In summary, the highly potent antibacterial activity of BMAP‐27B, along with its potential role as a broad‐spectrum antibiotic adjuvant, makes it a promising candidate for combating infections caused by foodborne NDM‐CRE and preventing pathogen transmission at the animal‐human‐environment interface.

## Impact statement

The rise of antibiotic resistance and superbugs in food animals is a major challenge in treating bacterial infections, which is seriously affecting human and animal health. We report that the peptide derivative BMAP‐27B shows excellent bactericidal activity and biofilm penetration against carbapenem‐resistant pathogens carrying New Delhi metallo‐β‐lactamase (NDM). Mechanistically, BMAP‐27B kills bacteria through multiple mechanisms, including enhancing cell membrane permeability and disrupting bacterial energy metabolism. Notably, subinhibitory concentrations of BMAP‐27B significantly restore the efficacy of carbapenem against NDM‐positive bacteria. Its antibacterial stability, favorable biocompatibility, and therapeutic potential highlight BMAP‐27B as a promising candidate for developing new treatment strategies against NDM‐CRE from food animals.

## INTRODUCTION

The overuse of antibiotics in clinical settings has resulted in a concerning increase in Carbapenem‐resistant *Enterobacteriaceae* (CRE)^1^. Notably, while carbapenems are not approved for use in food animal production, CRE has become widespread among food animals. This phenomenon can be attributed to the co‐selection of antibiotic resistance, particularly with agents like ceftiofur, which is permitted for use in livestock^2^. Research indicates that New Delhi metallo‐β‐lactamases (NDMs) play a crucial role in antibiotic resistance, allowing bacteria to withstand nearly all β‐lactam antibiotics, including carbapenems^3^. The catalytic function of NDMs relies on the binding of Zn^2+^ to the active site, which activates nucleophilic water to cleave the β‐lactam ring, thereby inactivating carbapenems^4^. Over 20 NDM variants have been identified, with NDM‐5 showing greater carbapenemase activity than NDM‐1^5^. These bacteria have rapidly acquired resistance to nearly all available antibiotics, earning them the label of “superbugs”, which significantly exacerbates the issue of bacterial resistance^6^. Furthermore, research has shown that the *bla*_NDM_ resistance genes are often located on transferable plasmids, facilitating transmission to humans via the food chain^7^. Given the lack of new antibiotics to combat the rapidly spreading and highly virulent CRE carrying NDM (NDM‐CRE), along with the impending reality of a post‐antibiotic era, there is an urgent need to identify effective and safe antimicrobials to control foodborne CRE pathogens.

Currently, the most cost‐effective strategy to combat NDM‐producing bacteria originating from food animals involves developing natural antimicrobials or utilizing antibiotic adjuvants to restore pathogen susceptibility to existing antibiotics^8^. Antimicrobial peptides (AMPs) represent a class of bioactive peptides with broad‐spectrum antibacterial activity and potential efficacy against multi‐drug‐resistant (MDR) bacteria. AMPs have garnered considerable attention as a novel type of bactericidal agents^9^, ^10^. These peptides primarily interact with bacterial membranes, causing physical damage that leads to cytoplasmic leakage and bacterial death^11^. Unlike traditional antibiotics, AMPs show minimal cross‐resistance and demonstrate a more pronounced bactericidal effect against drug‐resistant bacteria^12^. Significant progress has been made in applying AMPs as food additives, with nisin being widely utilized as a food preservative^13^. In 2024, Wei et al. reported a novel AMP, bacipeptin, which shows promise in the food industry due to its activity against foodborne pathogens^14^. Research into AMPs as antibiotic adjuvants has also substantially advanced, demonstrating their ability to enhance the intracellular accumulation of antibiotics by altering cell membrane permeability^15^, ^16^. However, the combined mechanisms by which AMPs restore the susceptibility of NDM producers to β‐lactam antibiotics remain unclear. Recently, the AMP thanatin has shown considerable promise as an antibiotic adjuvant by disrupting the bacterial outer membrane and displacing zinc ions from the active site of the NDM enzyme^17^. Similarly, further exploration of AMPs that can reduce resistance in CRE strains from food animals will expand treatment options for livestock, poultry, animal feed, and humans.

The cathelicidin family comprises a class of natural AMPs found in mammals, which exert bactericidal effects primarily by disrupting microbial cell membrane structures^18^. Among these, BMAP‐27, a peptide derived from cattle, has demonstrated significant antibacterial activity against Gram‐negative bacteria (G^‐^ bacteria). However, it was also found to cause reduced proliferation of mammalian cells. To address this issue, the sequence of BMAP‐27 was modified to create a new peptide, BMAP‐27B, which not only shows lower toxicity to mammalian cells but also decreases TLR3 signaling activation^19^. Previous studies have indicated that BMAP‐27B rapidly eliminates colistin‐resistant Gram‐negative bacteria and inhibits the growth of carbapenem‐resistant *Klebsiella pneumoniae* ^20^. However, it remains unclear whether BMAP‐27B exerts similar effects against NDM‐CRE bacteria found in food animals, and its specific mechanism of action as a carbapenem antibiotic adjuvant and bactericidal agent requires further investigation.

In this study, we examined the antibacterial activity of BMAP‐27B against NDM‐producing strains from food animals. BMAP‐27B significantly inhibited these strains and broadened its antibacterial spectrum against *Acinetobacter baumannii* and *Salmonella* was broadened. It acts as an antibiotic adjuvant, enhancing the effectiveness of meropenem (MEM) by inhibiting bacterial efflux pumps and chelating zinc ions at the NDM enzyme's active site, leading to increased intracellular antibiotic accumulation. BMAP‐27B also demonstrates stability in the presence of salt, serum, and proteases, showing excellent in vivo therapeutic efficacy without toxicity. These findings suggest that BMAP‐27B is a promising antimicrobial agent for treating infections caused by foodborne NDM‐CRE strains.

## RESULTS

### In vitro antibacterial activity of BMAP‐27B

Our investigation revealed a high prevalence of NDM‐CRE among food animals, particularly in pigs. These NDM‐CRE strains harbored multiple resistance genes, which highlights their formidable resistance profiles. Notably, the *bla*_NDM_ gene was frequently observed in conjunction with various extended‐spectrum beta‐lactamase (ESBL) genes (Table 1). Furthermore, multilocus sequence typing (MLST) analyses indicated that *bla*_NDM_‐like genes spread through horizontal transfer as well as clonal transmission across strains (Table 1). These results indicate a significant emergence of NDM‐CRE in food animals, with complex resistance mechanisms that pose a serious threat to public health.

**Table 1:** Isolation and identification of *bla*_NDM_‐positive strains.

Strain^a^	MLST	Resistance gene phenotype
CREC1	ST56	*bla*_NDM‐5_‐*bla*_CTX‐M‐9_‐*bla*_CTX‐U_‐*floR*‐*tetA*‐*tetB‐aac(6′)‐Ib*
CREC2	ST410	*bla*_NDM‐5_‐*floR‐cfr‐* *tetA*‐*aac(6′)‐Ib*
CREC3	ST10	*bla*_NDM‐5_‐*bla*_CTX‐M‐9_‐*bla*_TEM_ *‐floR*‐*tetA*‐*aac(6′)‐Ib*
CREC4	ST410	*bla*_NDM‐5_‐*bla*_CTX‐M‐9_‐*bla*_CTX‐U_‐*floR*‐*tetA*‐*tetB*‐*aac(6′)‐Ib*
CREC5	New	*bla*_NDM‐5_‐*bla*_TEM_ *‐floR*‐*tetA*‐*aac(6′)‐Ib*
CREC6	ST410	*bla*_NDM‐5_‐*bla*_TEM_ *‐floR‐cfr‐tetA*‐*aac(6′)‐Ib*
CREC7	ST410	*bla*_NDM‐5_‐*bla*_CMY2_ *‐floR*‐*tetA*‐*aac(6′)‐Ib*
CRCB1	ST19	*bla*_NDM‐1_‐*bla*_CMY2_ *‐floR*‐*tetA*‐*oqxA*‐*qnrB*‐*aac(6′)‐Ib*
CRCB2	ST19	*bla*_NDM‐1_‐*bla*_CMY2_ *‐floR*‐*tetA*‐*qnrB*‐*aac(6′)‐Ib*
CRCB3	ST19	*bla*_NDM‐1_‐*bla*_CMY2_ *‐floR*‐*tetA*‐*oqxA*‐*qnrB*‐*aac(6′)‐Ib*
CRKP1	ST629	*bla*_NDM‐5_‐*bla*_CMY2_ *‐floR*‐*tetA*‐*oqxA*‐*aac(6′)‐Ib‐fosA*
CRKP2	ST629	*bla*_NDM‐5_‐*bla*_CMY2_ *‐floR*‐*tetA*‐*oqxA*‐*aac(6′)‐Ib‐fosA*
CRKP3	ST629	*bla*_NDM‐5_‐*floR*‐*tetA*‐*oqxA*‐*aac(6′)‐Ib‐fosA*
CRKP4	ST629	*bla*_NDM‐5_‐*floR*‐*tetA*‐*oqxA*‐*aac(6′)‐Ib‐fosA*
CRKP5	ST629	*bla*_NDM‐5_‐*floR*‐*tetA*‐*oqxA*‐*aac(6′)‐Ib‐fosA*
CRKP6	ST629	*bla*_NDM‐5_‐*bla*_CMY2_ *‐floR*‐*tetA*‐*oqxA*‐*aac(6′)‐Ib‐fosA*

^a^ CREC, carbapenem‐resistant *Escherichia coli*; CRCB, carbapenem‐resistant *Citrobacter*; CRKP, carbapenem‐resistant *Klebsiella pneumonia*; *bla*_NDM‐5,_ carbapenem‐resistance gene; *bla*_CTX‐M,_ *bla*_TEM,_ *bla*_CMY2,_ extended‐spectrum beta‐lactamases (ESBL) genes; *floR* and *cfr*, chloramphenicol‐florfenicol resistance genes; MLST, multilocus sequence typing; *tetA*(*B*), tetracycline‐resistance genes; *oqxA* and *qnrB*, fluoroquinolone‐resistance genes; *fosA*, fosfomycin resistance gene.

**Figure 1:**
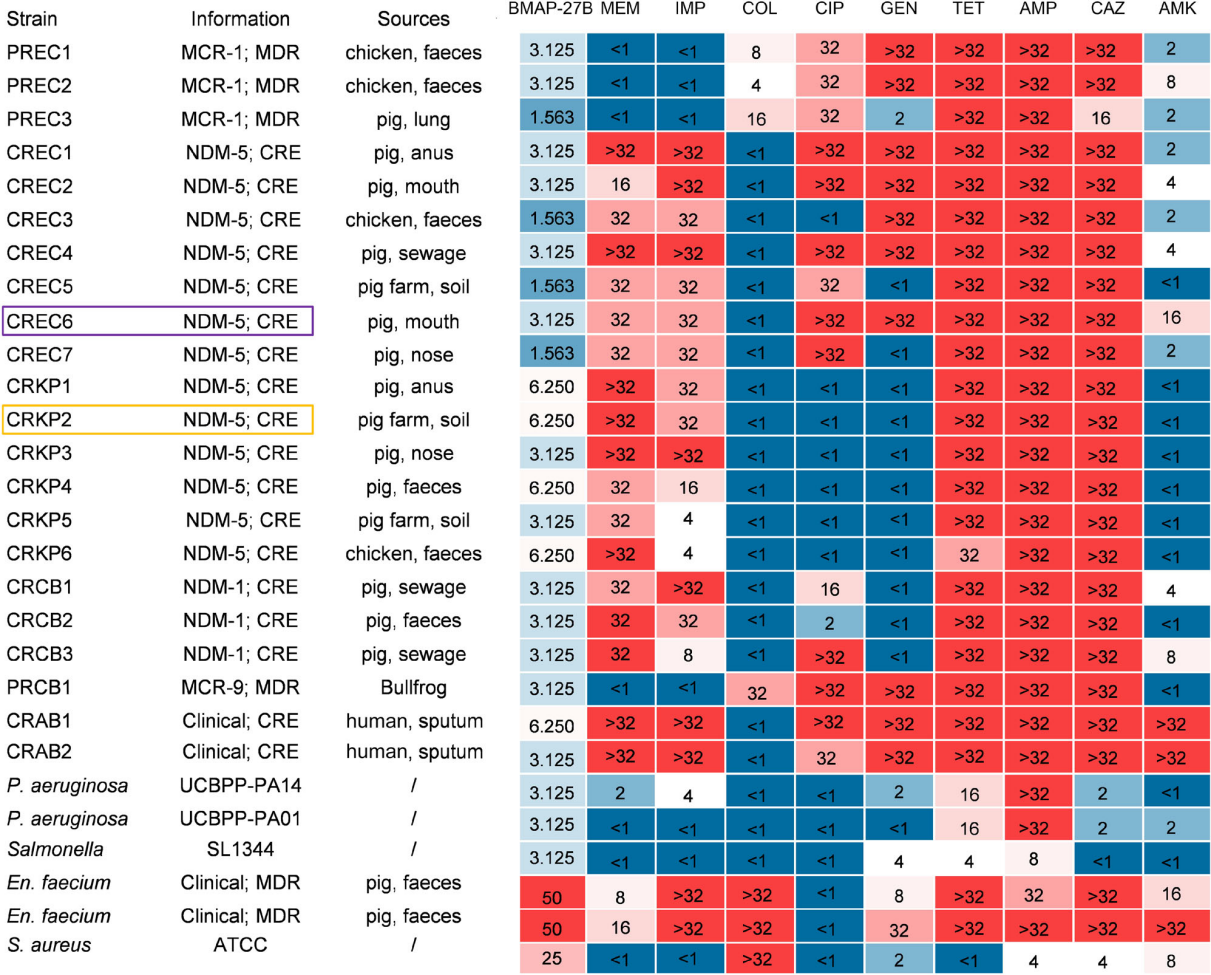
MICs of BMAP‐27B against multi‐drug‐resistant (MDR) Gram‐negative bacteria. AMK, amikacin; AMP, ampicillin; ATCC, American Type Culture Collection; CAZ, ceftazidime; CIP, ciprofloxacin; COL, colistin; CRAB, carbapenem‐resistant *Acinetobacter baumannii*; CRCB, carbapenem‐resistant *Citrate bacillus*; CRE, carbapenem‐resistant *Enterobacteriaceae*; CREC, carbapenem‐resistant *Escherichia coli*; CRKP, carbapenem‐resistant *Klebsiella pneumoniae*; *En. faecium*, *Enterococcus faecium*; GEN, gentamicin; IMP, imipenem; MCR, colistin‐resistance gene *mcr* encoded protein; MDR, multiple drug resistance; MEM, meropenem; NDM, carbapenem resistance gene *bla*_NDM_ encoded protein; PRCB, polymyxin‐resistant *Citrobacter*; PREC, polymyxin‐resistant *Escherichia coli*; *S. aureu*s, *Staphylococcus aureus*; TET, tetracycline. Experiments were performed in independent triplicates. The two strains boxed in yellow and purple gray were selected for the following studies.

In the broth microdilution antimicrobial susceptibility analysis, BMAP‐27B demonstrated significant inhibitory activity against Gram‐negative bacteria, including multi‐drug‐resistant (MDR) pathogens that produce MCR or NDM enzymes. Specifically, BMAP‐27B showed robust inhibitory effects on all NDM‐1/5‐producing CRE strains, with minimum inhibitory concentrations (MICs) ranging from 1.563 to 6.250 μg/ml (Figure 1). These CRE strains displayed varying levels of resistance to penicillin, ceftazidime, carbapenems, polymyxins, tetracycline, aminoglycosides, and quinolones. Additionally, BMAP‐27B showed strong in vitro antibacterial activity against *Acinetobacter baumannii* (MIC ≤ 6.250 μg/ml), which was resistant to carbapenems but did not produce NDM. BMAP‐27B also showed comparable bacteriostatic effects against MCR‐1/9‐producing Gram‐negative bacteria. In contrast, BMAP‐27B showed limited efficacy in inhibiting the growth of Gram‐positive bacteria that include *Staphylococcus aureus* and *Enterococcus faecium*, with MICs of ≥25 μg/ml (Figure 1). Overall, these findings suggest that BMAP‐27B possesses strong antibacterial activity and a broad antibacterial spectrum of efficacy against NDM‐positive bacteria.

### Rapid bactericidal efficiency of BMAP‐27B against Gram‐negative strains in vitro

**Figure 2:**
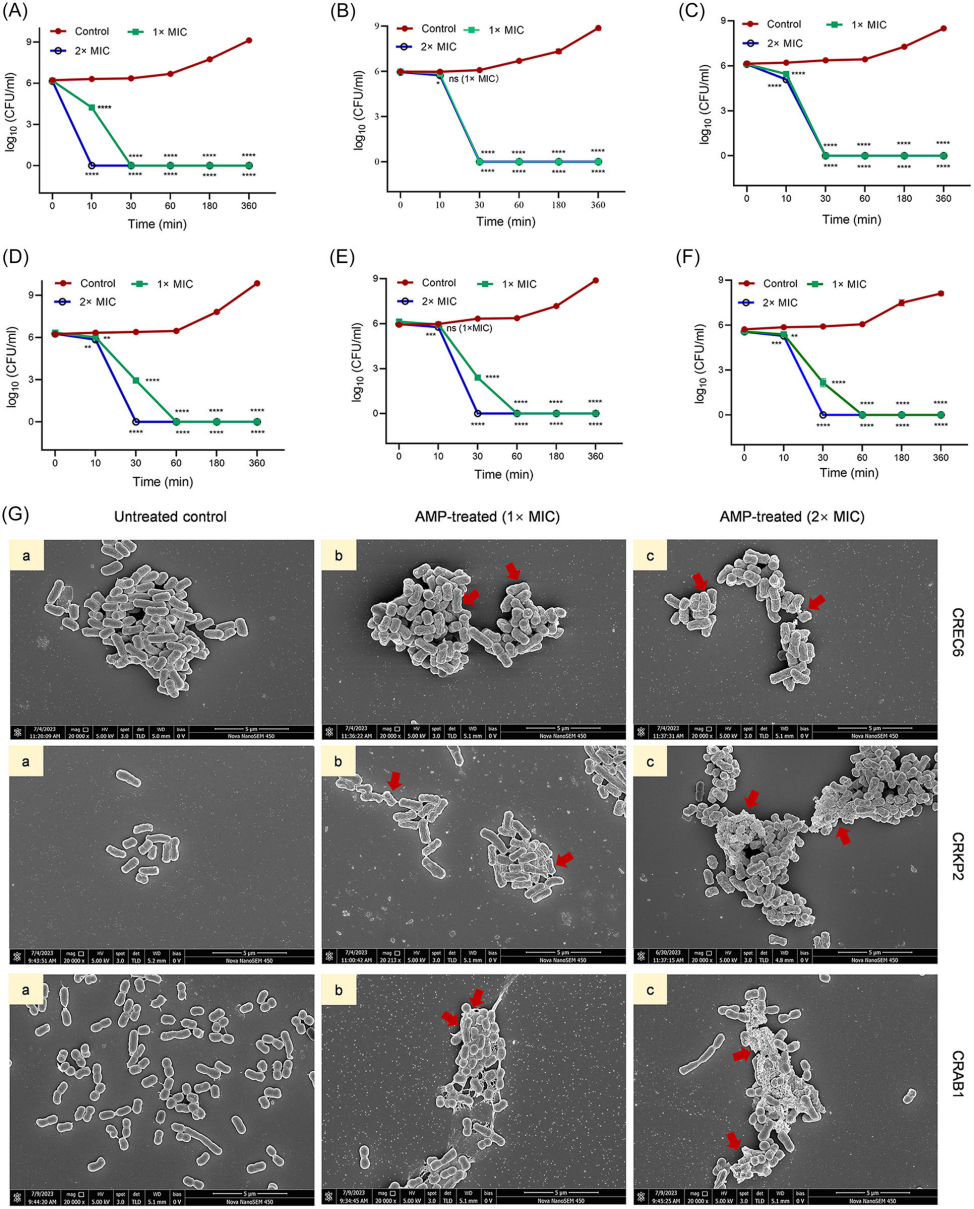
Time‐kill curves of BMAP‐27B against Gram‐negative bacteria and morphological observation of bacteria. (A–C) The colony counts of the New Delhi Metallo‐β‐lactamas(NDM)‐producing Gram‐negative bacteria including CRCB1 (A), CRKP2 (B), and CREC6 (C). (D–F) The colony counts of the other resistant bacteria including clinically isolated CRAB1 (D), *Salmonella* SL1344 (E), and *Pseudomonas aeruginosa* PA14 (F). The bacterial cells were treated with 1× MIC and 2× MIC concentrations of BMAP‐27B, and the colony counts at different time points were recorded using the drop plate method. (G) Morphological changes in CRKP2, CREC6, and CRAB1 investigated by scanning electron microscopy (SEM) 1 h after treatment with 1× MIC and 2× MIC concentrations of BMAP‐27B. Scale bar, 5 µm. Red arrows represent damaged bacteria. Data are expressed as mean ± SD, based on three biologically independent samples. Statistical significance was analyzed using an unpaired Student's *t*‐test. ns, not significant; *p < 0.05; **p < 0.01; ***p < 0.001; ****p < 0.0001. Comparison of results of statistical analysis of the colony counts at each time point between the treatment groups (1× MIC and 2× MIC) and the control group.

The bactericidal efficacy of BMAP‐27B against Gram‐negative strains was evaluated in vitro, revealing rapid killing kinetics. As shown in Figure 2, the kinetics study conducted at 1× MIC and 2× MIC concentrations of BMAP‐27B showed a pronounced bactericidal effect on NDM‐producing *Enterobacter*. Within 30 min of exposure, colony‐forming unit counts (CFUs) decreased by approximately 6 log units, with complete bacterial elimination achieved within 60 min of co‐incubation (Figure 2A–C). A similar trend was observed with carbapenem‐resistant *A. baumannii* (CRAB1), *Salmonella* SL1344, and *Pseudomonas aeruginosa* PA14 (Figure 2D–F). These results suggest that BMAP‐27B has a rapid bactericidal effect against MDR Gram‐negative bacteria. To further investigate the mechanism of action, scanning electron microscopy (SEM) was used to examine the morphological changes in bacterial cells following treatment with 1× MIC and 2× MIC concentrations of BMAP‐27B. SEM micrographs revealed that the cell structure and membrane of the control group remained intact, characterized by a smooth surface and short, slightly curved rods (Figure 2G, a). In contrast, after 1 h of treatment with 1× MIC BMAP‐27B, cells showed significant surface damage, including depressions, adhesions, wrinkles, and lysis. With increasing concentrations of BMAP‐27B, the proportion of bacterial cells displaying severe morphological alterations also increased, resulting in pronounced corrugation, membrane rupture, and the release of cellular contents (Figure 2G, b,c). These results suggest that the antibacterial effect of BMAP‐27B could be attributed to the rapid destruction of the bacterial cell membrane.

### Antimicrobial stability analysis of BMAP‐27B under different conditions

**Figure 3:**
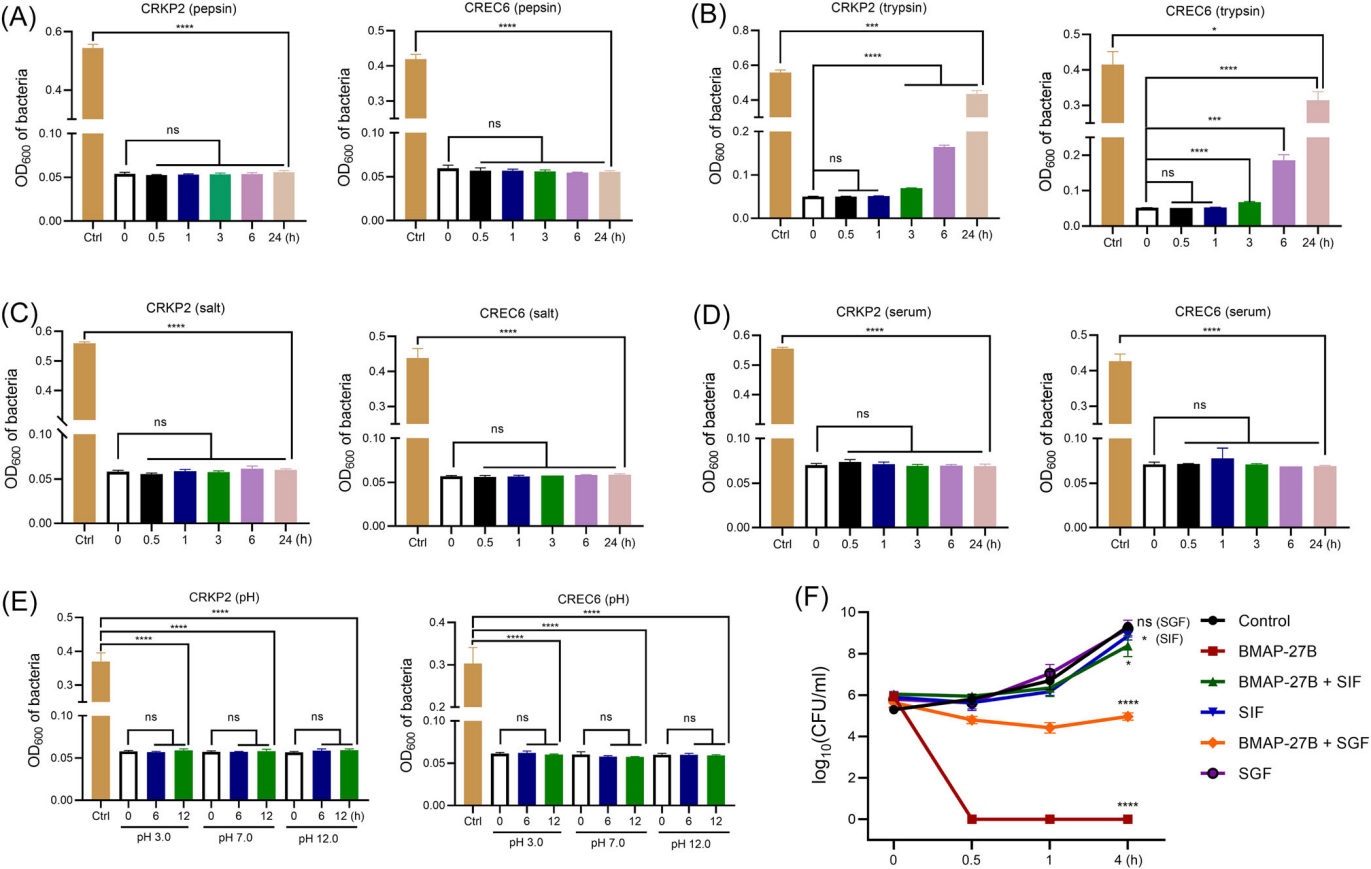
Antimicrobial stability of BMAP‐27B at a concentration of 1× MIC against CREC6 and CRKP2 under different conditions. (A, B) Bacteriostatic activity of BMAP‐27B against CRKP2 and CREC6 after incubation with pepsin (1 mg/ml) (A) or trypsin (1 mg/ml) (B) for 0–24 h. (C) Salt sensitivity of BMAP‐27B against CRKP2 and CREC6 at a final salt concentration of 400 mM for 0–24 h. (D) Serum stability of BMAP‐27B against CRKP2 and CREC6 in 25% fresh fetal bovine serum for 0–24 h. (E) pH stability of BMAP‐27B in PBS (pH 7.4), aqueous HCl (pH 3.0), and aqueous NaOH (pH 11.0) for 0, 6, and 12 h. (F) The stability of BMAP‐27B against CREC6 in simulated intestinal fluid (SIF) and simulated gastric fluid (SGF) evaluated using the drop plate method. Data are expressed as mean ± SD, based on three biologically independent samples. Statistical significance was analyzed using an unpaired Student's *t*‐test. ns, not significant; *p < 0.05; ***p < 0.001; ****p < 0.0001.

We analyzed the stability of BMAP‐27B under different conditions, including extreme pH, high NaCl concentration, and the presence of proteolytic enzymes and fetal bovine serum. After 24 h of co‐incubation with a 1 mg/ml pepsin solution, BMAP‐27B retained stable antimicrobial activity against the strains CREC6 and CRKP2 (Figure 3A). In contrast, BMAP‐27B showed poor stability in the presence of 1 mg/ml trypsin, with antibacterial activity gradually declining after 3 h, resulting in inhibition rates of 21.80% and 24.45% against CRKP2 and CREC6 after 24 h, respectively (Figure 3B). Furthermore, BMAP‐27B's antimicrobial activity was unaffected by the NaCl concentration, indicating its excellent salt tolerance in the presence of Na^+^ (Figure 3C). Notably, following 24 h of co‐incubation with 25% fetal bovine serum, BMAP‐27B maintained considerable efficacy against the tested strains (Figure 3D). In addition, concentrations of trypsin or proteinase K above 200 μg/ml resulted in a loss of BMAP‐27B's activity (Figure S1). The effect of pH on the antimicrobial activity of BMAP‐27B against NDM‐5‐producing bacteria was also examined. The presence of HCl (pH 3.0) or NaOH (pH 12.0) did not affect the peptide's activity, even after prolonged incubation (Figure 3E). Additionally, we tested BMAP‐27B's antimicrobial stability in simulated intestinal fluid (SIF) or simulated gastric fluid (SGF) to mimic the in vivo environment. As shown in Figure 3F, BMAP‐27B completely killed bacteria within 30 min, indicating potent bactericidal activity. However, in SIF, BMAP‐27B showed no activity, with bacterial counts comparable to those of the control group. Interestingly, after incubation with SGF for 4 h, the viable bacterial count was lower than that of the control group. Overall, BMAP‐27B has strong resistance against drastic pH changes, serum, pepsin, and high NaCl levels, while retaining some antibacterial activity in the gastric environment.

### BMAP‐27B reverses carbapenem resistance in vitro

**Figure 4:**
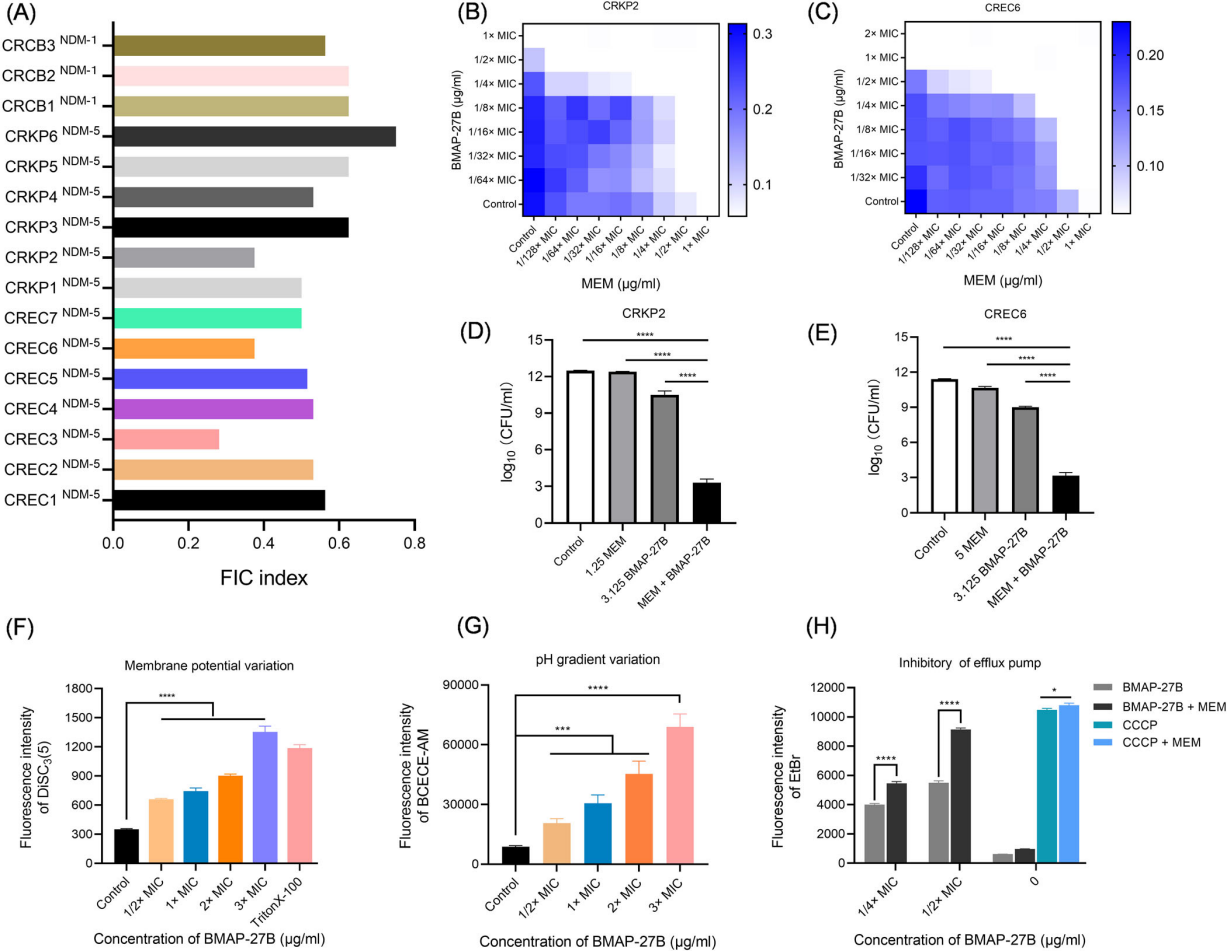
Synergistic potency of BMAP‐27B with MEM. (A) Synergistic effects of BMAP‐27B in combination with MEM against NDM‐producing Gram‐negative bacteria. The FIC index of 0.5 < FIC index < 1 indicates a synergistic effect. (B, C) Heat maps from microdilution checkerboard assays illustrating the combination of BMAP‐27B and MEM against CRKP2 (B) and CREC6 (C). Each heat map represents the average of three independent experiments. (D, E) Subinhibitory concentration of BMAP‐27B reversing the in vitro resistance of CRKP2 (D) and CREC6 (E) to MEM. (F, G) The proton motive force (PMF) destroyed by BMAP‐27B. Changes in Δψ (F) and ΔpH (G) were observed after treatment with different concentrations of BMAP‐27B for 1 h. (H) Inhibitory effect of BMAP‐27B on the CREC6 efflux pump. The accumulation of EtBr was measured to assess the activity of efflux pumps induced by BMAP‐27B, MEM, or their combination. Data are expressed as mean ± SD, based on three biologically independent samples. Statistical significance was analyzed using an unpaired Student's *t*‐test. *p < 0.05; ***p < 0.001; ****p < 0.0001. BCECE‐AM, 2′,7′‐bis‐(2‐carboxyethyl)‐5‐(and‐6)‐carboxyfluorescein, acetoxymethyl ester; CCCP, carbonyl cyanide m‐chlorophenyl hydrazone; DiSC3‐5, 3,3‐dipropylthiadicarbocyanine iodide; CCCP + MEM, the combination of CCCP and meropenem. 1.25 MEM and 5 MEM refer to 1.25 μg/ml and 5 μg/ml of MEM, respectively; 3.125 BMAP‐27B refer to 3.125 μg/ml of BMAP‐27B.

The synergistic activities of BMAP‐27B in combination with carbapenems were first determined using a checkerboard assay against 16 CRE isolates. We observed that the combination of BMAP‐27B and MEM showed synergistic or additive effects on NDM‐producing bacteria, with fractional inhibitory concentration (FIC) indices of ≤1 (Figure 4A). Notably, the presence of subinhibitory concentration (sub‐MIC) BMAP‐27B significantly restored the antibacterial efficacy of MEM against NDM‐5‐producing *K. pneumoniae* and *E. coli* (CRKP2 and CREC6), reducing the MIC value by ≥ 16‐fold (Figure 4B,C). Furthermore, we assessed the bactericidal potency of the combination of BMAP‐27B and MEM against the strains CRKP2 and CREC6. As shown in Figure 4D,E, the combination of sub‐MIC BMAP‐27B with MEM markedly decreased bacterial CFUs compared to monotherapy, indicating a considerable bactericidal effect against NDM‐CRE.

To determine whether BMAP‐27B targets the bacterial cytoplasmic membrane, we investigated its damaging effects on membrane components, specifically the cytoplasmic membrane potential (Δψ) and the pH gradient (ΔpH). We measured the fluorescence intensity of the 3,3‐dipropylthiadicarbocyanine iodide (DiSC3‐5) probe in CREC6, observing significant fluorescence enhancement with increasing concentrations of BMAP‐27B, indicating that Δψ was disrupted (Figure 4F). The ΔpH of the strain was measured using the fluorescent probe 2′,7′‐bis‐(2‐carboxyethyl)‐5‐(and‐6)‐carboxyfluorescein, acetoxymethyl ester (BCECF‐AM). The fluorescence absorption values showed a trend similar to that of Δψ, suggesting that BMAP‐27B severely disrupted ΔpH in the bacteria (Figure 4G). These findings indicate that BMAP‐27B dissipates the proton motive force (PMF) by disrupting both Δψ and ΔpH. PMF is essential for the function of bacterial efflux pumps, which play a major role in antibiotic resistance^21^. To investigate the inhibitory effect of BMAP‐27B on bacterial efflux pumps, we conducted an ethidium bromide (EtBr) accumulation assay. Compared to the groups treated with peptide alone, the combination of 1/2× MIC BMAP‐27B and 1/2× MIC MEM significantly enhanced fluorescence intensity (Figures 4H and S2). These results suggest that BMAP‐27B effectively inhibits the efflux pumping activity of NDM‐positive bacteria, which is crucial for combating antibiotic resistance.

### Binding ability of BMAP‐27B to lipopolysaccharides (LPS) and disruption of membrane permeability

**Figure 5:**
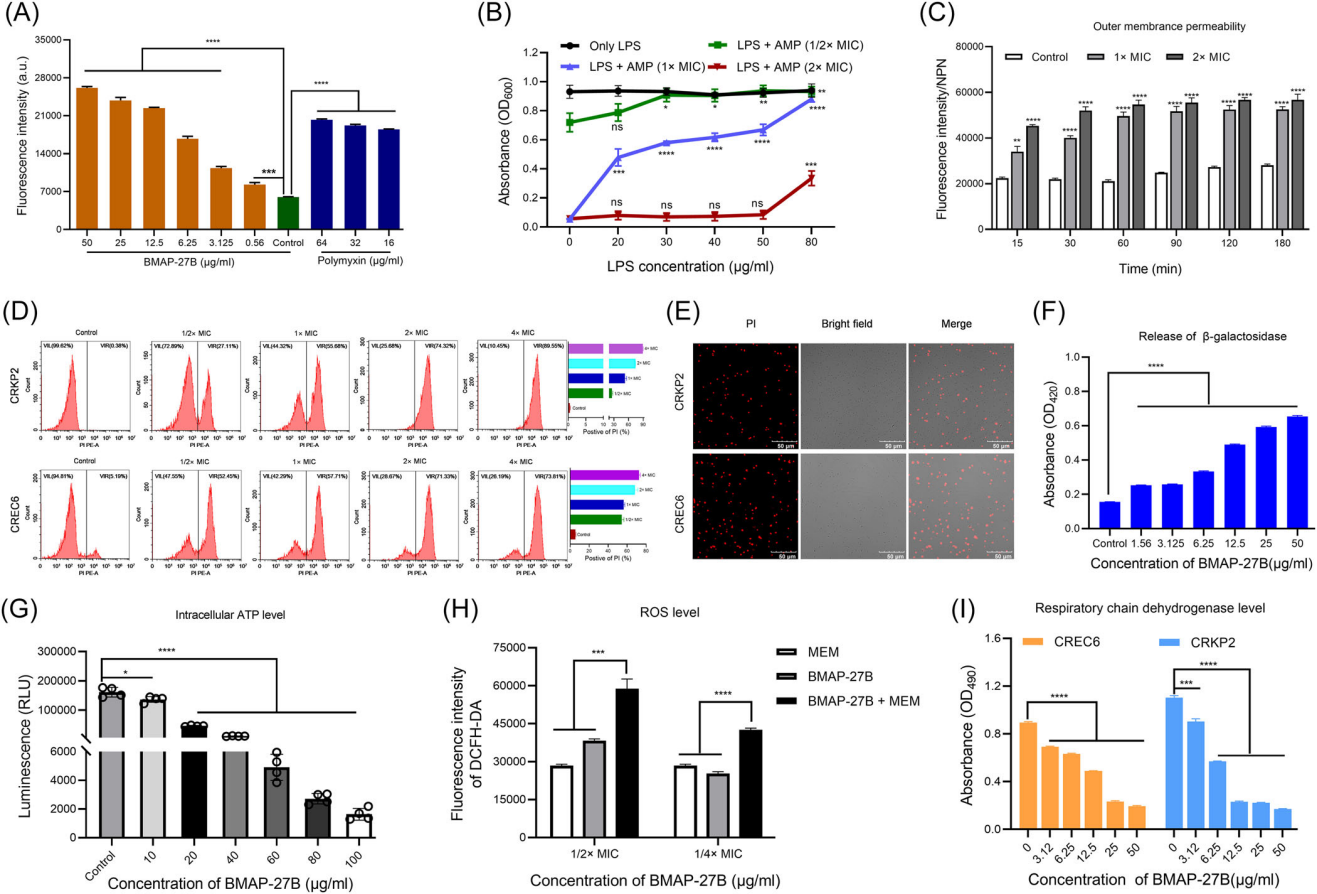
Bactericidal mechanism of BMAP‐27B against carbapenem‐resistant bacteria. (A) Binding affinity of peptide BMAP‐27B to lipopolysaccharides (LPS). LPS binding affinity of polymyxin was used as a positive control. (B) Effect of different concentrations of LPS on the antibacterial activity of BMAP‐27B at 1/2, 1, and 2× MIC against CREC6. (C) Outer membrane permeabilization of CREC6 after treatment with different concentrations and durations of BMAP‐27. (D) Propidium iodide (PI) uptake assay for membrane integrity of CRKP2 and CREC6. The histogram on the right quantifies the proportion of PI‐positive stained cells. (E) Confocal microscopy images of CRKP2 and CREC6 under the treatment of 1× MIC BMAP‐27B at 37°C for 1 h. The red signal indicates PI. Scale bar, 50 µm. (F) Release of β‐galactosidase from CREC6 after treatment with different concentrations of BMAP‐27B for 2 h. (G) Changes in the level of intracellular ATP in CREC6 treated with different concentrations of BMAP‐27B for 1 h. (H) Reactive oxygen species (ROS) accumulation in CREC6 treated with BMAP‐27B (1/2× MIC or 1/4× MIC), MEM (1/2× MIC), and their combinations for 30 min. (I) Inhibitory effect on respiratory chain dehydrogenase in CREC6 and CRKP2 after treatment with different concentrations of BMAP‐27B for 3 h. Data are expressed as mean ± SD, based on three biologically independent samples. Statistical significance was analyzed using an unpaired Student's *t*‐test. ns, not significant; *p < 0.05; **p < 0.01; ***p < 0.001; ****p < 0.0001.

LPS is a major component of the Gram‐negative bacterial membrane and a key contributor to the negative charge on the bacterial surface^22^. Therefore, we used CREC6 as a model to test the interaction between BMAP‐27B and LPS. We initially used the BODIPY‐TR‐cadaverine (BC) fluorescent probe replacement method to detect the binding affinity of BMAP‐27B for LPS. As shown in Figure 5A, BMAP‐27B showed strong binding affinity for LPS in a dose‐dependent manner. When the concentration of BMAP‐27B exceeded 6.25 µg/ml, its LPS binding affinity was comparable to that of polymyxin. Furthermore, we examined the impact of the interaction between LPS and BMAP‐27B on antimicrobial activity, revealing that the addition of exogenous purified LPS derived from *E. coli* (055: B5) significantly inhibited the antimicrobial efficacy of BMAP‐27B. Under a treatment condition of 1× MIC, the inhibitory effect became more obvious with increasing LPS concentration, and at the highest tested LPS concentration (80 μg/ml), BMAP‐27B almost lost its antibacterial activity (Figure 5B).

Subsequently, we assessed the effect of BMAP‐27B on outer membrane permeability, and the results showed that the degree of permeability increased with the BMAP‐27B concentration (Figures 5C and S3). Additionally, the propidium iodide (PI) uptake assay revealed a concentration‐dependent increase in the proportion of PI‐positive bacteria, reaching 89.5% and 73.81% at 4× MIC in CRKP2 and CREC6, respectively, suggesting enhanced permeability of the inner membrane (Figure 5D). Confocal laser scanning microscopy (CLSM) further corroborated these findings, showing that red fluorescent bacteria labeled with PI predominantly occupied the bacterial area in the bright field (Figures 5E and S4). After a 2‐h incubation with BMAP‐27B, we observed a concentration‐dependent increase in the release of cytoplasmic β‐galactosidase (Figure 5F). Collectively, these results indicate that BMAP‐27B exerts its bactericidal activity by disrupting membrane integrity.

### BMAP‐27B impairs the energy metabolism of MDR bacteria

The levels of intracellular adenosine triphosphate (ATP) in *E. coli* CREC6 after treatment with BMAP‐27B were analyzed. We observed that BMAP‐27B significantly reduced the intracellular ATP content in a dose‐dependent manner (Figure 5G). Correspondingly, the extracellular ATP level increased in a dose‐dependent manner (Figure S5). Previous studies have shown that the disruption of membrane homeostasis can lead to the accumulation of reactive oxygen species (ROS)^23^, ^24^. We thus aimed to evaluate the effects of BMAP‐27B on ROS accumulation in bacterial cells. As shown in Figures 5H and S6, treatment with BMAP‐27B resulted in a significant and dose and dependent increase in intracellular ROS levels in both CREC6 and CRKP2. Notably, the combined treatment with BMAP‐27B and MEM further amplified ROS accumulation. These results indicate that bacterial cells show a pronounced stress response following BMAP‐27B treatment, resulting in substantial ROS release. To further explore the impact of BMAP‐27B on bacterial energy metabolism, we evaluated its effects on the activity of respiratory chain dehydrogenases. As shown in Figure 5I, BMAP‐27B strongly inhibited respiratory chain dehydrogenase activity in CREC6 and CRKP2 in a dose‐dependent manner. Specifically, at concentrations of BMAP‐27B up to 50 μg/ml, the respiratory chain dehydrogenases activity was inhibited by 78% and 84% in CREC6 and CRKP2, respectively. These data suggest that BMAP‐27B impairs bacterial survival and proliferation by disrupting respiration and energy metabolism.

### Penetration and eradication of biofilms by BMAP‐27B on NDM‐5‐producing *E. coli* and *K. pneumoniae*

**Figure 6:**
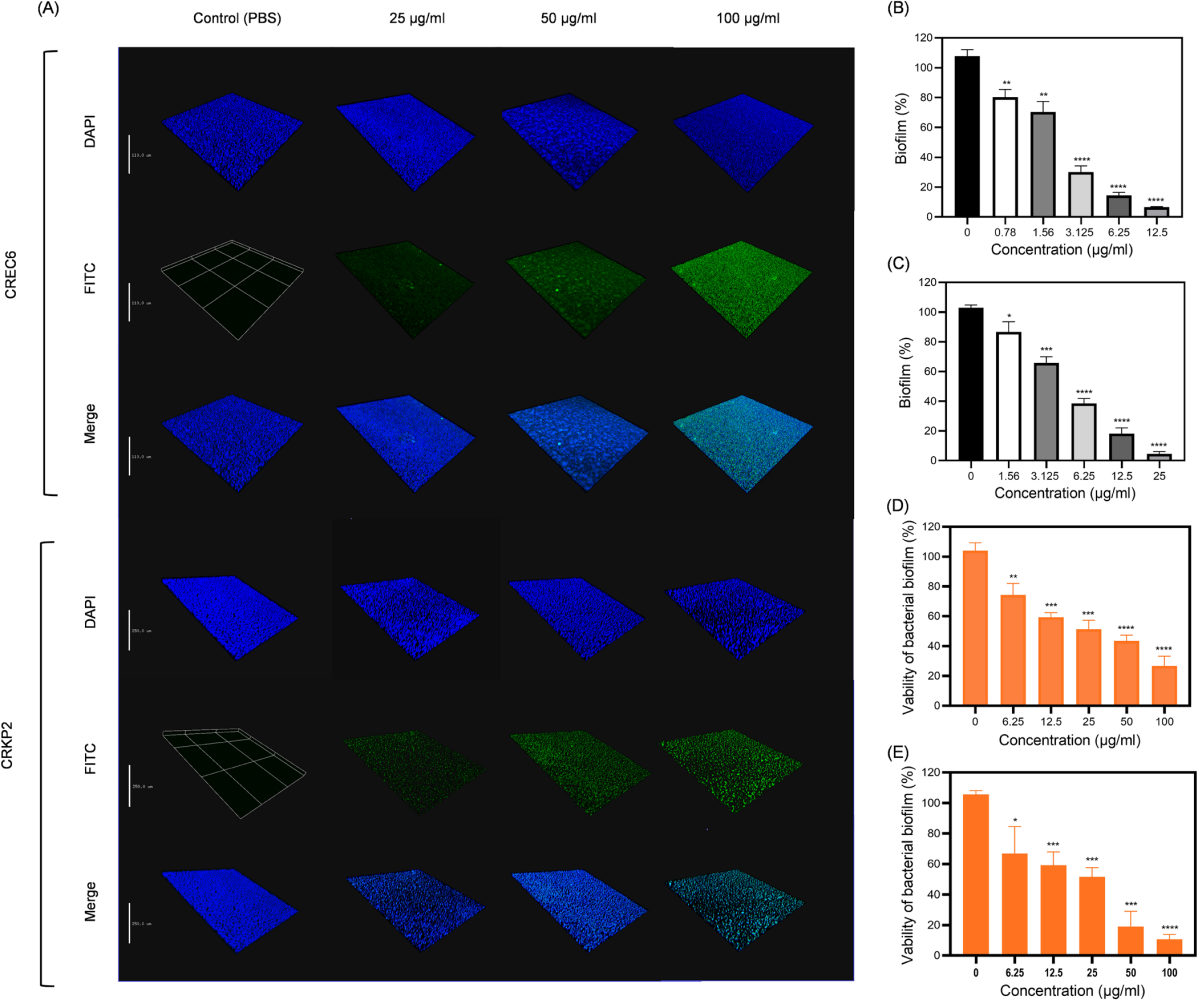
Penetration and eradication of biofilms by peptide BMAP‐27B. (A) Confocal laser scanning microscopy (CLSM) images of NDM‐5‐producing CREC6 and CRKP2 biofilms after incubation with PBS or FITC‐labeled BMAP‐27B (blue fluorescence, biofilms of bacteria stained by 4′,6‐diamidino‐2‐phenylindole (DAPI); green fluorescence, FITC‐peptide BMAP‐27B). The scale bars for CREC6 and CRKP2 are 110 and 250 µm, respectively. (B, C) Biomass of NDM‐5‐producing CREC6 (B) and CRKP2 (C) biofilms after treatment with different concentrations of BMAP‐27B. Results are expressed as the biofilm percentage, measured using crystal violet staining. (D, E) Effect of peptide BMAP‐27B on bacterial viability within the mature biofilms of NDM‐5‐producing CREC6 (D) and CRKP2 (E). Data are expressed as mean ± SD, based on three biologically independent samples. Statistical significance was analyzed using an unpaired Student's *t*‐test. *p < 0.05; **p < 0.01; ***p < 0.001; ****p < 0.0001.

Biofilms are multicellular aggregates encased in extracellular polymers, and their formation is closely associated with antibiotic resistance and virulence of bacteria. To investigate the potential anti‐biofilm mechanism of BMAP‐27B, we evaluated the peptide's ability to penetrate mature biofilms of CREC6 and CRKP2. In this experiment, fluorescein isothiocyanate (FITC)‐labeled BMAP‐27B was incubated with the bacterial biofilms at 37°C for 3 h. The results indicated minimal green fluorescence within the biofilms at a peptide concentration of 25 μg/ml, with the fluorescence intensity increasing as higher peptide concentrations were used (Figure 6A). Notably, at a concentration of 100 μg/ml, a pronounced fluorescence signal was detected in the deeper layers of the biofilms. These results suggested that BMAP‐27B was effective in penetrating the biofilm, with its penetration ability positively correlated with the peptide concentration.

The antibiofilm activity of BMAP‐27B against the CREC6 and CRKP2 strains was assessed using a crystal violet staining assay. At concentrations below 1.56 μg/ml, BMAP‐27B showed minimal effects on preformed bacterial biofilms. However, at concentrations ranging from 0.78 to 25 μg/ml, BMAP‐27B significantly reduced biofilm biomass (Figure 6B,C), indicating the peptide's ability to disrupt biofilm integrity. Additionally, the methylthiazolyldiphenyl‐tetrazolium bromide (MTT) assay was used to quantify bacterial viability within the biofilms of CREC6 and CRKP2. The results showed that increasing concentrations of BMAP‐27B corresponded to a higher bacterial death rate in the CREC6 and CRKP2 biofilms (Figure 6D,E). Overall, these findings suggest that BMAP‐27B has the potential to treat infections caused by biofilm‐forming bacteria.

### BMAP‐27B is localized within the cell membranes of NDM‐producing *K. pneumoniae* and *E. coli*

**Figure 7:**
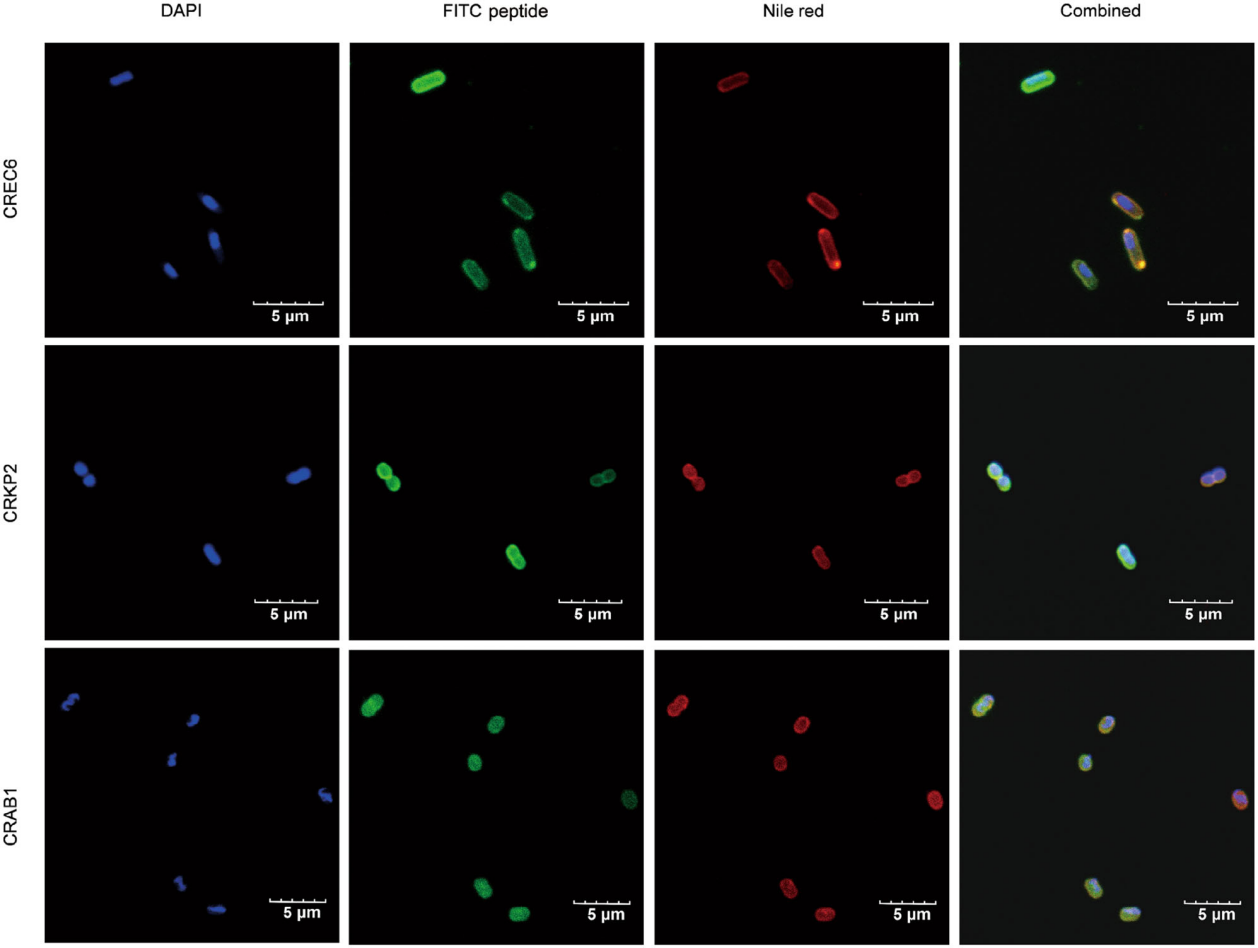
CLSM images of CREC6, CRKP2, and CRAB1 after treatment with FITC‐labeled BMAP‐27B, Nile red, and DAPI. BMAP‐27B is colocalized with Nile red, indicating a membrane‐binding propensity. Blue fluorescence, nuclei of bacteria stained by DAPI; green fluorescence, FITC‐peptide BMAP‐27B; red fluorescence, cell membrane of bacteria stained by Nile red. Scale bar, 5 µm.

In a confocal microscopy experiment, we investigated the colocalization of BMAP‐27B within bacterial cells. Specifically, we used CRE strains of CREC6, CRKP2, and CRAB1 for this test. The isolated strains were stained with FITC‐labeled BMAP‐27B, Nile red (a lipophilic cell membrane dye), and 4′,6‐diamidino‐2‐phenylindole (DAPI) (a nucleic acid dye) to track the interaction process between BMAP‐27B and the bacterial cells. The colocalization analysis revealed a significant overlap between BMAP‐27B and Nile red signals in CREC6, CRKP2, and CRAB1, indicating that BMAP‐27B acts on the bacterial membrane (Figure 7).

### In vivo biocompatibility of BMAP‐27B

**Figure 8:**
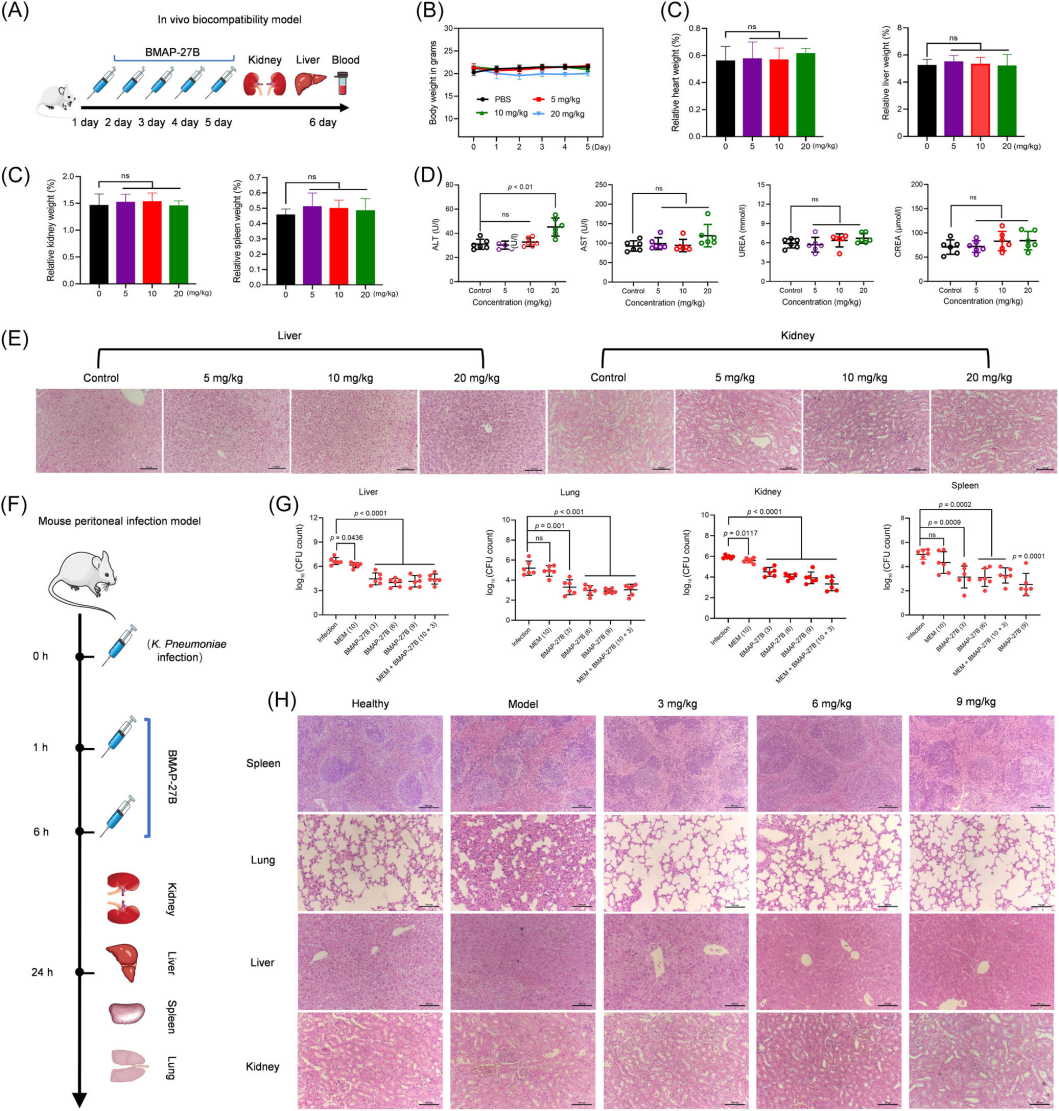
Evaluation of in vivo toxicity and efficacy of BMAP‐27B. (A) Schematic diagram illustrating the in vivo biocompatibility of multiple doses of BMAP‐27B injected intraperitoneally into mice. (B) Daily monitoring of body weight changes in mice over 6 days following administration of PBS or BMAP‐27B (5, 10, or 20 mg/kg). (C) Assessment of relative organ weights for the heart, liver, spleen, and kidneys in mice after an intraperitoneal injection of multiple doses of BMAP‐27B. (D) Measurement of liver function indexes (ALT and AST) and renal function indexes (CREA and UREA) in mice post‐administration of PBS or BMAP‐27B. (E) Histopathological hematoxylin and eosin (H&E) staining of mouse liver and kidney tissues treated with PBS or different concentrations of BMAP‐27B. Scale bar, 100 µm. (F) Schematic diagram illustrating the mouse peritoneal infection and treatment model induced by CRKP2. (G) Evaluation of bacterial load changes in the liver, spleen, lungs, and kidneys of mice following treatment with BMAP‐27B monotherapy or in combination with MEM in the mouse peritoneal infection model. (H) Histopathological H&E staining of spleen (scale bar, 200 µm), lung, liver, and kidney tissues from healthy, infected, and BMAP‐27B‐treated groups (scale bar, 100 µm). Healthy group: received PBS injections at 0, 1, and 6 h; Model group: received CRKP2 suspension injections at 0 h and treated with PBS at 1 and 6 h; BMAP‐27B‐treated group: received CRKP2 suspension injections at 0 h and treated with BMAP‐27B at concentrations of 3, 6, and 9 mg/kg at 1 and 6 h. Statistical significance was analyzed using an unpaired Student's *t*‐test (ns, not significant; *p < 0.05; ***p < 0.001; ****p < 0.0001). ALT, alanine aminotransferase; AST, aminotransferase; CREA, creatinine; UREA, urea. MEM (10): treated with meropenem at concentrations of 10 mg/kg; BMAP‐27B (3), BMAP‐27B (6), BMAP‐27B (9): treated with BMAP‐27B at concentrations of 3, 6, or 9 mg/kg; MEM + BMAP‐27B (10+3): combined treatment with BMAP‐27B at a concentration of 3 mg/kg and meropenem at a concentration of 10 mg/kg.

The hemolytic activity of BMAP‐27B was measured, and at a concentration of 250 μg/ml, the hemolytic activity remained relatively low (Figure S7). To evaluate in vivo biocompatibility, 6 BALB/c mice per group received intraperitoneal injections of either saline or different doses of BMAP‐27B (5, 10, and 20 mg/kg) for 6 days at 24‐h intervals. Potential adverse effects of BMAP‐27B were monitored through changes in body weight, blood parameters, and organ‐related indices (Figure 8A). After administration, mice in the high‐dose group (20 mg/kg) showed temporary piloerection and inactivity, which resolved after 3 h. Although all groups showed initial weight loss on the first day postinjection, body weight increased over the 5‐day treatment period (Figure 8B). Analysis indicated no significant differences in the relative weights of the heart, liver, spleen, and kidneys among the groups (Figure 8C). Additionally, liver and kidney function indicators in the BMAP treatment groups, including aspartate aminotransferase (AST), creatinine (CREA), and urea (UREA), remained within the normal range and showed no statistical differences compared to the control group (Figure 8D). However, a modest increase in alanine aminotransferase (ALT) levels was observed in the high‐dose treatment group (20 mg/kg), suggesting a potential mild adverse effect on liver function. Hematoxylin and eosin (H&E) staining revealed no discernible pathological changes in the liver and kidneys of mice treated with BMAP‐27B at doses of 5, 10, and 20 mg/kg (Figure 8E). Collectively, these data indicated that BMAP‐27B does not induce primary hepatorenal toxicity at doses up to 20 mg/kg.

### In vivo efficacy of BMAP‐27B

We evaluated the in vivo efficacy of BMAP‐27B using a mouse peritonitis model induced by CRKP2. Following an intraperitoneal injection of the bacterial solution, infection and treatment groups received saline, MEM, or BMAP‐27B at 1 and 6 h, respectively. Tissue samples were collected 24 h after infection (Figure 8F). Bacterial CFUs were analyzed, revealing that BMAP‐27B monotherapy and the combination with MEM significantly reduced bacterial titers in the liver, spleen, lung, and kidneys compared to the infection group (Figure 8G). In contrast, the bacterial titers in the MEM monotherapy group were only slightly lower than those in the infection group (Figure 8G). Histopathological analysis showed tissue injuries in the infection group, characterized by decreased lymphocyte populations in the spleen, alveolar fusion, thickened respiratory membranes, multiple microvacuoles, and hepatocyte congestion, along with notable hemorrhage in the kidneys (Figure 8H). However, BMAP‐27B monotherapy therapy substantially mitigated these pathological changes, underscoring the potential of BMAP‐27B as a therapeutic agent for bacterial infections in vivo.

### Molecular docking study of BMAP‐27B and NDMs enzyme

**Figure 9:**
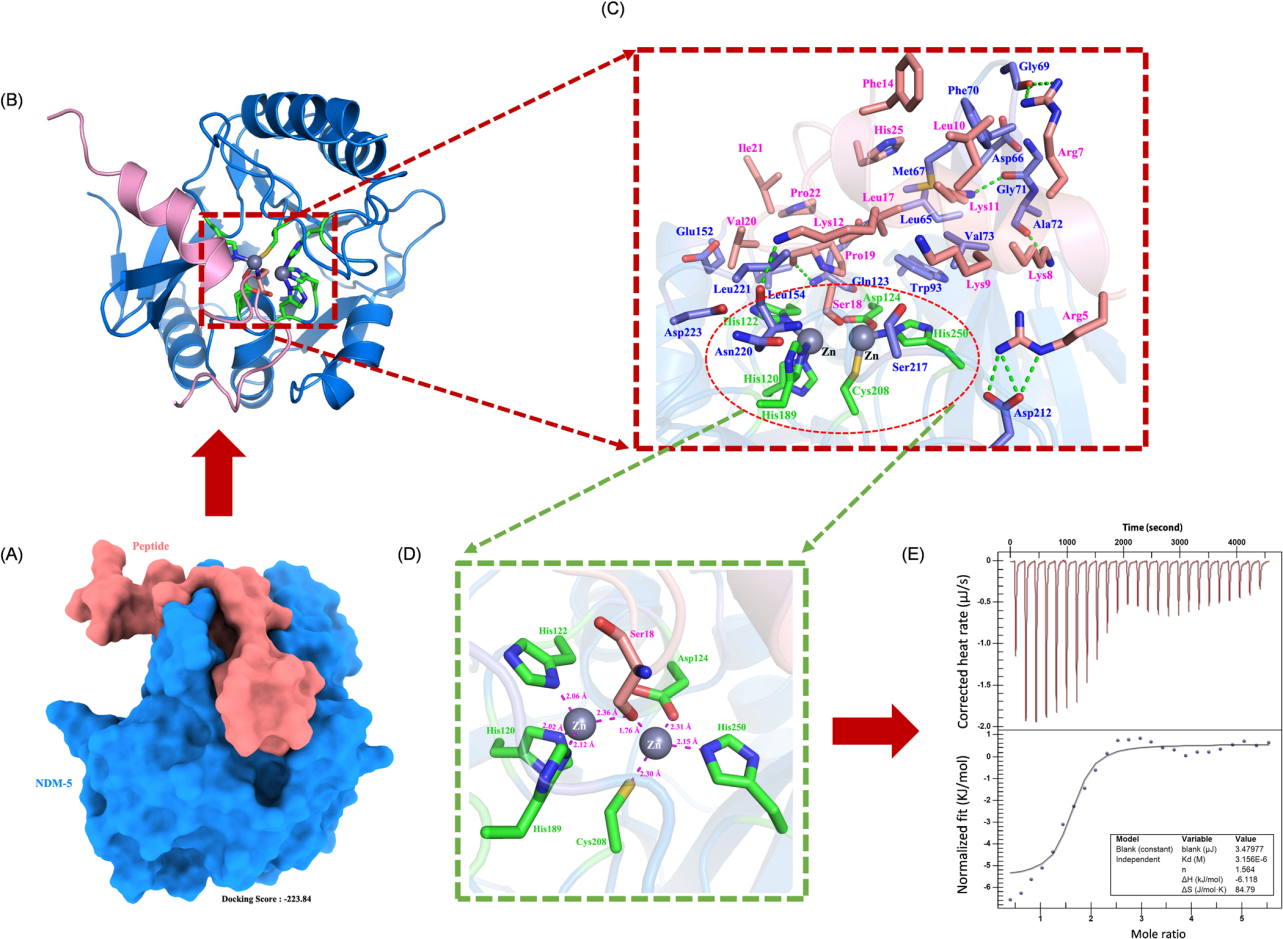
BMAP‐27B disrupts the activity of NDM‐5 by chelating zinc ions. (A) Structural representation of the complex formed by docking NDM‐5 with peptide molecules using the Hybrid Docking (HDOCK) program. (B) Interaction patterns between BMAP‐27B and the NDM‐5 protein. (C, D) Binding patterns between amino acid residues of peptide molecules and the NDM‐5 active site (the dotted green line shows hydrogen bonding). (E) Isothermal titration calorimetry (ITC) thermograms for the binding of BMAP‐27B to Zn^2+^. The downward peaks indicate an exothermic process.

The optimal docking result of NDM‐5 and the peptide molecule BMAP‐27B is shown in Figure 9A, with a confidence score of 0.814. BMAP‐27B fits well into the grooves of the NDM‐5 protein, facilitating favorable shape complementarity. Further analysis revealed the formation of 6 hydrogen bond interactions between the NDM‐5 and BMAP‐27B, with key amino acids involved in these interactions being Gly69, Gly71, Ala72, Gln123, Asp212, and Asn220 (Figure 9B,C). Additionally, a substantial number of hydrophobic residues contribute to strong hydrophobic interactions that enhance binding affinity (Table S1). To confirm the inhibitory effect of the peptide on NDM‐5, we analyzed its binding mode in the active center of NDM‐5. It was observed that Ser18 in the peptide forms a stable complex with two zinc ions at distances of 2.36 Å and 1.76 Å, indicating stable binding of the peptide within the active site of NDM‐5 (Figure 9D). Similarly, BMAP‐27B showed stable coordination with zinc ions in the active site of NDM‐1 at distances of 2.13 and 2.37 Å (Figure S8, Table S2). To provide direct evidence of binding, we examined the affinity of BMAP‐27B for zinc ions using isothermal titration calorimetry (ITC), which revealed an equilibrium dissociation constant (*K*_d_) of 3.156 µM, indicating a strong affinity of BMAP‐27B for zinc ions (Figure 9E).

## DISCUSSION

Consistent with previous studies, we found that NDM‐CRE strains remain widespread in food animals^7^, ^25^. Most of these strains show an MDR profile and carry multiple resistance genes, facilitating the spread of *bla*_NDM_‐like genes among different hosts. This underscores the urgent need to identify effective antimicrobial treatments against NDM‐CRE. However, the emergence of resistance to tigecycline and colistin, which are considered “last‐resort” antibiotics, has compromised their efficacy^26^, ^27^. Recent studies have shown that AMPs, as a novel type of antimicrobial compounds, are promising against CRE^28^. In this study, we demonstrated that the peptide BMAP‐27B shows excellent antibacterial efficacy against polymyxin‐resistant Gram‐negative bacteria, as reported in previous studies^19^. Additionally, our results indicated that BMAP‐27B can rapidly kill NDM‐CRE isolated from food animals as well as carbapenem‐resistant clinical strains of *A. baumannii*, thereby expanding the antibacterial spectrum of BMAP‐27B. Recent studies have reported that animal‐derived carbapenem‐resistant *Enterobacteriaceae* pose a significant threat to public health^25^, ^29^. Therefore, we propose the use of BMAP‐27B as a potential agent to combat NDM‐producing bacteria and reduce the transmission of CRE resistance in food animals.

**Figure 10:**
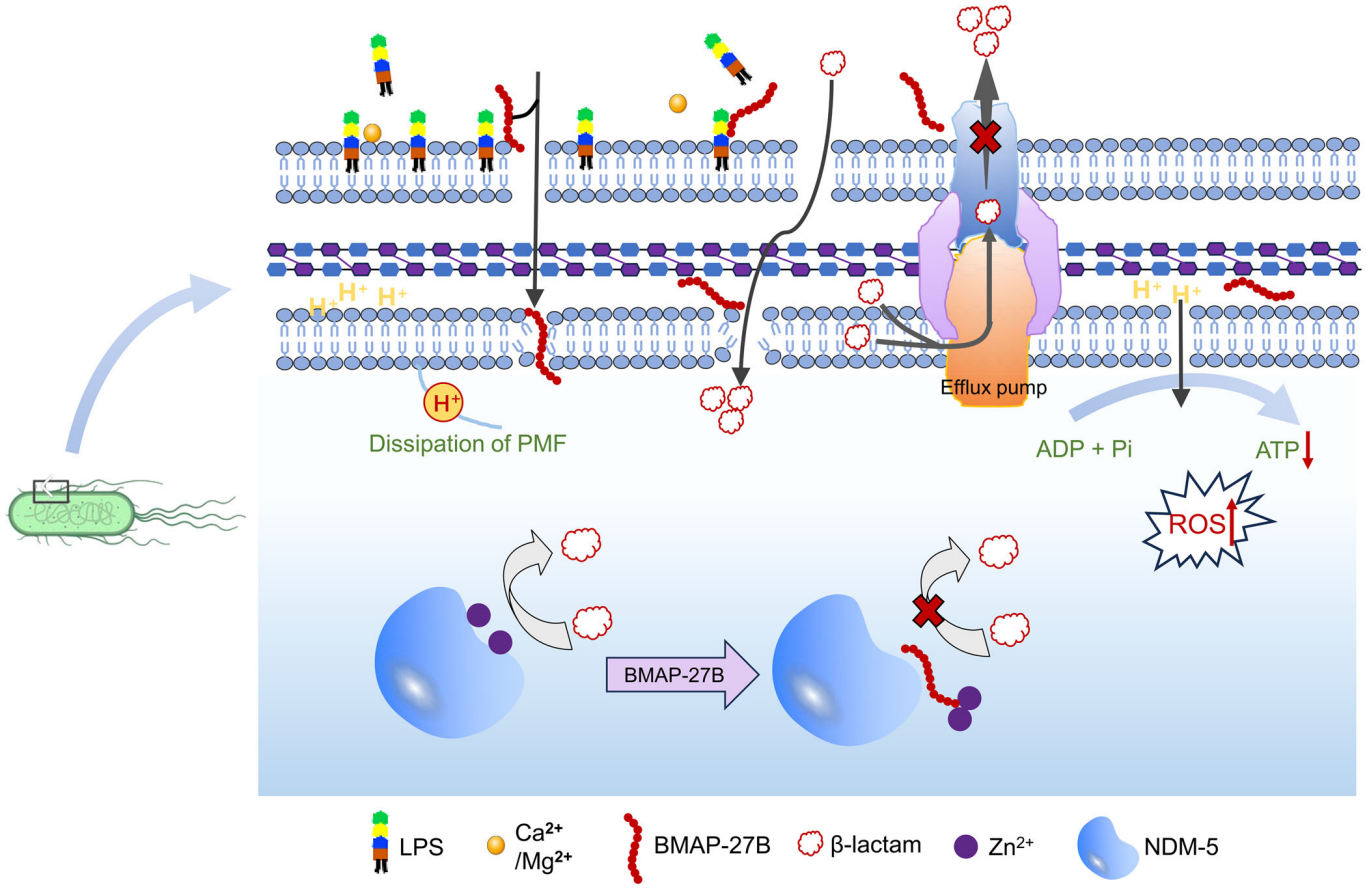
Mechanism of action of BMAP‐27B against MDR Gram‐negative bacteria. BMAP‐27B exerts a bactericidal effect by disrupting bacterial cell membranes and inhibiting bacterial energy metabolism. BMAP‐27B restores the sensitivity of carbapenem‐resistant bacteria to antibiotics by inhibiting the dual modes of efflux pump activity and metallo‐β‐lactamase activity. PMF, proton motive force; ROS, reactive oxygen species.

LPS is a major component of Gram‐negative bacteria and is responsible for maintaining the structural stability of their cell membranes^30^. Consequently, antimicrobials that target bacterial cell membranes hold significant therapeutic potential^31^. Our results showed that the antibacterial mechanism of BMAP‐27B resembles that of antibiotics targeting LPS^32^. BMAP‐27B binds to LPS in a concentration‐dependent manner through electrostatic interactions, leading to the aggregation of peptide molecules on the membrane surface, resulting in increased membrane permeability, membrane rupture, and the release of cellular contents. In this study, we found that BMAP‐27B‐induced membrane damage disrupts respiratory chain function, leading to ROS accumulation and inhibition of respiratory chain dehydrogenase activity. These changes impaired bacterial energy metabolism, as evidenced by a suppression of ATP synthesis following BMAP‐27B treatment. Consistent with previous studies, AMPs or antibiotics can increase the ROS production, triggering oxidative stress responses that damage bacterial cells^24^, ^33^, ^34^. Taken together, BMAP‐27B effectively kills bacteria by simultaneously disrupting the bacterial membrane and inhibiting energy metabolism (Figure 10).

The PMF generated across the cell membrane is crucial for bacterial survival and pathogenicity^35^. As expected, BMAP‐27B significantly disrupts both ΔΨ and ΔpH, leading to PMF dissipation. Consequently, when BMAP‐27B interacts with bacteria, it compromises membrane integrity and collapses PMF, hindering bacteria's ability to acquire essential nutrients and energy, ultimately resulting in bacterial death^36^. Furthermore, PMF is linked to bacterial resistance mechanisms; for instance, efflux pumps rely on PMF to decrease intracellular antibiotic concentrations^37^. Currently, the search for efflux pump inhibitors has become a major strategy to prevent the transmission of MDR bacteria from animals to humans. A recent study reported that tetrandrine enhances the antimicrobial effects of colistin by inhibiting the activity of *Salmonella* efflux pumps^38^. In our study, the EtBr accumulation assay demonstrated that sub‐MICs of BMAP‐27B, whether used alone or in combination with MEM, significantly inhibited the efflux pump function to a similar extent as the reported efflux pump inhibitor carbonyl cyanide m‐chlorophenyl hydrazone (CCCP). These results suggest that BMAP‐27B disrupts the function of PMF‐driven efflux pumps in bacteria, preventing the efflux of MEM, thereby restoring the activity of carbapenems against NDM‐producing strains. Considering the limitations of antibiotic monotherapy and the spread of resistance, the development of combination therapies using BMAP‐27B and antibiotics represents a promising strategy.

In clinical contexts, NDM inhibitors are used to reduce the development of bacterial resistance to carbapenem antibiotics by antagonizing the catalytic activity of metallo‐β‐lactamase (MBL)^39^. These inhibitors are often combined with β‐lactam antibiotics to enhance their bactericidal activity, such as avibactam^40^. In addition to chelating zinc ions at the active site, MBL inhibitors can inhibit the hydrolytic activity of NDM proteases by displacing zinc ions. For example, the antimicrobial peptide thanatin acts as an NDM‐1 inhibitor by competitively displacing zinc ions^17^. A recent study showed that the liposomal antibiotic booster (M‐MFL@MB) irreversibly inactivated NDM‐1 protease by removing Zn^2+^ from NDM‐1^41^. Therefore, Zn^2+^ is an ideal target for MBL inhibitors. In our study, molecular docking analysis revealed that BMAP‐27B interacts with the amino acids of NDM‐1/5 through hydrogen bonds and hydrophobic interactions. Notably, BMAP‐27B can form a stable complex with Zn^2+^ in the active region of NDM, leading to a loss of NDM activity. These findings suggest that BMAP‐27B has the potential to become an NDM inhibitor.

The stability of AMPs is significantly affected by their external environment, which poses a major challenge for the development and application as effective antibacterial agents. Some AMPs have demonstrated enhanced stability through sequence or chemical modifications^42^. For instance, MSI‐1, derived from the truncated form of MSI‐78, shows exceptional stability under extreme pH values and high NaCl concentrations^43^. BMAP‐27B is a derivative generated by modifying the amino acid composition of BMAP‐27. In our study, BMAP‐27B interacts excellent tolerance to pH changes and high salt concentrations, while maintaining potent antimicrobial activity even after exposure to serum or pepsin. This indicates its excellent potential for application in antibacterial therapies. Despite being a cationic AMP, our analysis revealed that BMAP‐27B showed low hemolytic activity, thereby reducing toxicity to mammalian cells^19^. Biofilms play a crucial role in bacterial virulence. By inhibiting biofilm formation and development, the spread of pathogenic bacteria in food can be reduced, thereby extending food shelf‐life^44^. Importantly, we found that BMAP‐27B had strong permeability in biofilm environments and has shown potential therapeutic effects against biofilm‐forming bacteria in vivo. Collectively, this study confirms that BMAP‐27B is an effective antibacterial agent against NDM‐CRE derived from food animals.

In summary, our study highlights the potent antibacterial efficacy of BMAP‐27B against NDM‐CRE strains from food animals. By targeting the bacterial cell membrane and disrupting energy metabolism, BMAP‐27B effectively induces bacterial death, while also enhancing the effectiveness of carbapenems through efflux pump inhibition. BMAP‐27B also shows promise as an NDM inhibitor by binding to Zn^2+^ in metallo‐β‐lactamases, making it suitable for combination therapies with traditional antibiotics. Additionally, the peptide's stability under various environmental conditions and low hemolytic activity further suggest its clinical applicability. Overall, BMAP‐27B demonstrates potential as a novel antimicrobial agent to combat the growing threat of NDM‐producing bacteria.

## METHODS AND MATERIALS

### Bacterial strains

*E. coli* ATCC 25922, *K. pneumoniae* ATCC 700603, *S. aureus*, *P. aeruginosa* PA14 and PA01, and *Salmonella* SL1344 were preserved in our laboratory. Carbapenem‐resistant *A. baumannii* strains (AbC17, Ab18) were obtained from human clinical specimens at the Medical Laboratory Center of Guangzhou People's Hospital. Additionally, carbapenem‐resistant strains of *E. coli*, *K. pneumoniae*, and *Citrobacter*, as well as clinical isolates of *E. faecium*, were obtained from farm animals (pigs and chickens) in Guangxi, China. The methods used for isolating NDM‐CRE and for MLST detection are detailed in the Supporting Information section.

### Synthesis of BMAP‐27B

BMAP‐27B (GRFKRLRKKLKKLFKKLSPVIPLLHLG) was synthesized using 9‐fluorenylmethyloxycarbonyl (Fmoc)‐based SPPS at GL Biotech Co., Ltd (Shanghai, China). Briefly, 0.5 g of Fmoc‐Gly Wang Resin was placed into a sand core reactor, and then an appropriate amount of dichloromethane (DCM) was added, followed by soaking for 5 min. The solution was then removed under vacuum. The resin was treated with a deprotection solution (DMF: piperidine = 1:4) for 10 min to remove the Fmoc protecting group; this step was repeated using a 20% piperidine solution, followed by washing with DMF nine times. Then, the corresponding amounts of Fmoc‐Leu‐OH amino acid, condensation agent TBTU, and *N*‐methylmorpholine were added, and the mixture was washed with DMF 6 times after reacting for 1 h. The above steps were repeated until the final amino acid, Fmoc‐Gly‐OH, was successfully connected. Then, the Fmoc protecting group was removed and the resin was washed with DMF nine times. The peptide was dried with methanol and subsequently cut. The crude peptide was then purified using reverse‐phase high‐performance liquid chromatography (RP‐HPLC) to achieve a purity exceeding 95%.

### Antimicrobial susceptibility assay

The MIC of BMAP‐27B and other antibiotics was determined using the broth microdilution method, following the guidelines established by the Clinical and Laboratory Standards Institute (CLSI). Briefly, the bacterial cultures were grown to the logarithmic growth phase and subsequently diluted in Mueller–Hinton broth (MHB) to achieve a final concentration of 1 × 10^6^ CFU/ml. BMAP‐27B and the other antibiotics were prepared in twofold serial dilutions in MHB and mixed with an equal volume of bacterial suspension in 96‐well polypropylene microtiter plates. After incubation for 16–18 h at 37°C, the MIC was defined as the lowest concentration of the antibiotics that showed no significant bacterial growth, with MHB‐containing bacteria serving as the positive control.

### Bactericidal killing kinetics assay

The drop plate method was used to evaluate the time‐kill curves of Gram‐negative bacteria. A bacterial suspension at a final concentration of 1 × 10^6^ CFU/ml was added to the peptide solution at concentrations of 1× MIC and 2× MIC. Samples of the mixture were collected at various time intervals (0, 10, 30, 60, 180, and 240 min), serially diluted, and evenly plated onto agar plates. Following overnight incubation at 37°C, the antimicrobial kinetics of the peptide were assessed by counting the colonies on the plates. A bacterial suspension without peptide treatment served as the negative control. Each test was independently repeated at least three times.

### SEM characterization

The morphological characteristics of MDR *E. coli*, *K. pneumoniae*, and *A. baumannii* treated with BMAP‐27B were examined using SEM. Briefly, bacteria in the logarithmic growth phase were diluted in PBS to an OD_600_ of 0.4‐0.6 and subsequently cultured in the presence of BMAP‐27B at concentrations of 1× MIC and 2× MIC at 37°C for 1 h. Following incubation, samples were centrifuged and the supernatant was discarded. The bacterial pellets were fixed with 2.5% glutaraldehyde at 4°C for 24 h. Control samples were treated with Luria–Bertani (LB) broth without the peptide. The fixed samples were gradually dehydrated using a series of ethanol concentrations (30%, 50%, 70%, 80%, 90%, and 100%). The processed samples were then dried using supercritical carbon dioxide (CO_2_), coated with gold‐palladium, and examined using SEM, with images captured for analysis.

### Antibacterial stability analysis of BMAP‐27B

For the protease and serum stability assays, 1× MIC of the peptide was incubated with 1 mg/ml pepsin, 1 mg/ml trypsin, or 25% fetal bovine serum for 0.5, 1, 3, 6, and 24 h at 37°C. The antibacterial activity of the treated peptide was determined using CREC6 and CRKP2 as models. Bacterial colony survival was quantified by measuring the absorbance at 600 nm using a microplate reader. The activity of the peptide was also determined in the presence of NaCl to assess its salt sensitivity, with a final NaCl concentration of 400 mM. Additionally, 1× MIC of BMAP‐27B was incubated in PBS at pH 7.0, aqueous HCl at pH 3.0, and aqueous NaOH at pH 11.0 for 6 and 12 h at 37°C, after which the activity of the treated peptide was recorded. To evaluate the impact of SIF or SGF on the antimicrobial stability of BMAP‐27B, the peptide was mixed with SIF or SGF and then added to a diluted bacterial solution, achieving a final peptide concentration of 1× MIC. The bactericidal efficacy of BMAP‐27B against bacteria in SIF or SGF was evaluated using the plate counting method at different time points.

### FIC detection

The synergistic effect of BMAP‐27B and MEM was assessed in vitro using checkerboard assays. Different concentrations of BMAP‐27B and MEM were serially diluted and mixed in 96‐well plates. A bacterial solution at a final concentration of 0.5 to 1 × 10^6^ CFU/ml was added to each well. After incubation at 37°C for 14 to 16 h, the OD_600_ value was measured to evaluate bacterial growth. The synergistic or additive effect was determined by calculating the FIC index, defined as follows: FICI = (MIC of A in combination/MIC of A) + (MIC of B in combination/MIC of B). Based on standard criteria, an FIC index of 0.5 < FIC index < 1 indicates a synergistic effect, 1 ≤ FIC index < 4 indicates no interaction, and FIC index > 4 indicates an antagonistic effect. To further analyze the bactericidal effect of BMAP‐27B in combination with MEM, bacterial cells were diluted in MHB to a concentration of 1 × 10^6^ CFU/ml and incubated with 1/2× MIC of either BMAP‐27B, MEM, or their combination for 16 h. Finally, the CFUs were counted to determine bacterial survival.

### LPS binding assay

The binding affinity of BMAP‐27 for LPS was assessed using the fluorescent probe BC. LPS (50 μg/ml) derived from *E. coli* (055: B5) was incubated with BC (5 μg/ml) in PBS in the dark for 4 h. Following incubation, the LPS–BC mixture was combined with an equal volume of a peptide diluent and was transferred to a 96‐well plate. BMAP‐27B was then added at concentrations ranging from 0.56 to 50 μg/ml, and the plate was incubated at 37°C for 1 h. Fluorescence intensities were measured at excitation and emission wavelengths of 580 and 620 nm, respectively, with polymyxin included as a positive control. To evaluate the interaction between LPS and BMAP‐27B, the antimicrobial activity of BMAP‐27B against *E. coli* CREC6 was tested at 1/2× MIC, 1× MIC, and 2× MIC in the presence of increasing concentrations of LPS. The LPS–BMAP–27B mixture was added to an *E. coli* suspension and incubated for 16 h. Ultimately, bacterial growth was assessed by measuring the optical density at 600 nm.

### Outer and inner membrane permeability assay

The outer membrane permeability of BMAP‐27B was measured through the uptake of *N*‐phenyl‐1‐naphthylamine (NPN). Briefly, CREC6 and CRKP2 in the logarithmic growth phase were diluted in PBS (pH 7.4) to an OD_600_ of 0.4. Then, 10 μM NPN was added to the bacterial suspension and incubated in the dark for 30 min. Following this incubation, the mixture was incubated with varying concentrations of peptide at a ratio of 1:1 (v/v; 100 µl/100 µl) for an additional 30 min. Fluorescence was measured (excitation *λ* = 350 nm, emission *λ* = 420 nm) using a fluorescence spectrophotometer. In bacterial inner permeability analysis, CREC6 and CRKP2 were mixed with varying concentrations of BMAP‐27B and incubated at 37°C for 1 h. PI was subsequently added to the suspension at a final concentration of 20 μg/ml and incubated in the dark for 30 min. Membrane integrity was further assessed using flow cytometry and CLSM. Furthermore, a bacterial suspension was treated with varying concentrations of BMAP‐27B for 1 h at 37°C. The suspension was then centrifuged and the supernatant was collected. *O*‐nitrophenyl‐β‐d‐galactopyranoside (ONPG) was added to the collected supernatant at a final concentration of 1 mg/ml and incubated at 37°C for 30 min. The absorbance at 420 nm was measured and recorded.

### PMF assay

Changes in the cytoplasmic membrane potential (Δ ψ) of bacteria were assessed using the lipophilic membrane probe 3,3‐dipropylthiadicarbocyanine iodide (DiSC3‐5). Bacterial cells in the logarithmic growth phase were washed and diluted to an OD_600_ of 0.3 in HEPES (5 mM, pH 7.2) supplemented with glucose (5 mM). The bacterial suspension was then combined with DiSC3‐5 dye at a concentration of 1 μM and incubated in the dark for 30 min. The mixture was combined with the peptide solution in a 96‐well plate at a 1:1 (v/v) ratio and incubated at 37°C for 1 h. Fluorescence values were recorded using a fluorescence spectrophotometer, with an excitation wavelength of 620 nm and an emission wavelength of 670 nm. Triton X‐100 (0.1%) was used as a positive control. The pH gradient (ΔpH) of bacteria was assessed using the pH‐sensitive fluorescent probe BCECF‐AM. The bacterial suspension described previously was incubated with BCECF‐AM at a concentration of 4 μM in the dark for 30 min. An equal volume of the mixture was then incubated with a preprepared peptide solution for 1 h at 37°C. The excitation and emission wavelengths were 488 and 535 nm, respectively.

### ROS and ATP determination

The levels of ROS in CREC6 and CRKP2 strains following treatment with either the peptide or MEM were measured using 2′,7′‐dichlorofluorescein diacetate (DCFH‐DA). DCFH‐DA was added to the bacterial suspension to achieve a final concentration of 10 μM, and the mixture was incubated at 37°C for 30 min. Subsequently, peptide solution at concentrations of 1/2× MIC or 1/4× MIC, along with a subinhibitory concentration of MEM, were added to the probe‐labeled bacterial cells in a 96‐well plate. After additional incubation for 30 min, fluorescence intensity was measured immediately using a fluorescence spectrophotometer (excitation *λ* = 488 nm, emission *λ* = 525 nm). Extracellular and intracellular ATP levels in bacteria were determined using the ATP Assay Kit (Beyotime). First, different concentrations of BMAP‐27B were added to the bacterial suspension and incubated for 1 h. Then, the suspension was centrifuged to collect the supernatant for the measurement of extracellular ATP levels. The bacterial pellet was lysed using cell lysis solutions, followed by centrifugation, and the supernatant was prepared for the determination of intracellular ATP levels.

### Respiratory chain inhibition assay

In this study, red tetrazolium (RT) was used to measure the respiratory chain dehydrogenase activity of bacteria treated with BMAP‐27B. Bacterial cells were cultured to the logarithmic growth phase and subsequently diluted to an OD_600_ of 0.5 in Tris buffer (0.05 M, pH = 8.6). Following this, equal Tris HCl buffer (0.05 M, pH = 8.6), glucose (0.1 M), and RT (1 mg/ml) were added sequentially to the bacterial suspension. Different concentrations of BMAP‐27B were then introduced into the prepared mixture. OD_490_ measurements were recorded immediately following a 3‐h incubation at 37°C. Sterile water was used as a blank control.

### Efflux pump suppression assay

Inhibition of the bacterial efflux pump was assessed by measuring the accumulation of ethidium bromide (EtBr), as previously described^45^. A final concentration of 5 μM EtBr was added to the bacterial suspension prepared in PBS and incubated at 37°C for 30 min. Subsequently, 1 ml of the mixture containing either 1/2 or 1/4× MIC of BMAP‐27B, a subinhibitory concentration of MEM, or a combination of both was incubated at 37°C for an additional hour. A 100 μM concentration of the efflux pump inhibitor CCCP served as a positive control. Fluorescence intensity was measured with an excitation wavelength of 530 nm and an emission wavelength of 600 nm.

### Penetration ability of BMAP‐27B on biofilms

In this study, the penetration ability of BMAP‐27B into biofilms was evaluated using CLSM. Briefly, the CREC6 and CRKP2 strains in the logarithmic growth phase were adjusted to an OD_600_ of 0.4 in PBS (pH 7.4). The cells were then inoculated onto glass‐bottom petri dishes and incubated at 37°C for 24 h. Once mature biofilms formed, the supernatant was discarded and the biofilms were washed twice with PBS to remove any planktonic bacteria. FITC‐labeled BMAP‐27B was subsequently added to the biofilm samples at a final concentration ranging from 25 to 100 μg/ml. After a 3‐h incubation at 37°C, DAPI dye was added to the samples at a concentration of 20 μg/ml and incubated for an additional 30 min. Excess FITC‐labeled peptide and DAPI dye were washed away with PBS, and the biofilms were examined using CLSM. Biofilm viability was assessed using the MTT assay. A total of 100 µl of peptide solution, with concentrations ranging from 3.125 to 100 μg/ml, was added to the mature biofilm and incubated for 3 h. Subsequently, 20 µl of MTT solution (0.5 mg/ml) was added to each well, followed by a further 2‐h incubation at 37°C in the dark. The solution was subsequently discarded, and 150 µl of dimethyl sulfoxide (DMSO) was added to dissolve the formazan crystals. Finally, absorbance was measured at 570 nm using a microplate reader and results were reported as percentages of biofilm viability.

### Biofilm biomass detection

The clearing effect of BMAP‐27B on bacterial biofilms was detected using the crystal violet staining method. Exponentially growing bacteria were inoculated into MHB medium to an OD_600_ of 0.4. The bacterial suspensions were then aliquoted into 96‐well plates and serially diluted peptide solutions (ranging from 0.78 to 50 μg/ml) were added, with PBS without peptide serving as a control. After incubation at 37°C for 24 h to allow for biofilm formation, the planktonic bacteria were discarded and the plates were washed twice with PBS. The biofilms were subsequently stained with a 1% crystal violet solution for 15 min. Following rinsing with deionized water, the stained biofilms were solubilized in 95% ethanol. Finally, absorbance at 595 nm (OD_595_) was measured using a microplate reader to assess biofilm formation.

### Localization of FITC‐labeled BMAP‐27B with bacteria

The subcellular localization of BMAP‐27B was investigated using CLSM. A FITC‐labeled peptide was synthesized, and the bacterial cell membrane and nuclei were stained with Nile Red and DAPI, respectively. Bacterial cells in the logarithmic growth phase were harvested, washed, and resuspended in PBS to achieve an OD_600_ of 0.3. The FITC‐labeled peptide was then added to the culture at a final concentration of 1/2× MIC and incubated at 37°C for 30 min in the dark. Excess FITC‐labeled peptide was removed by washing with PBS and the cells were resuspended in 1 ml of PBS. Subsequently, DAPI and Nile red dyes were added at concentrations of 5 and 2 μg/ml, respectively, while keeping the culture in the dark. After 20 min of incubation, a small volume (3 µl) of the stained culture was transferred to a clean glass slide and covered with a glass coverslip. CLSM was used for imaging.

### In vivo biocompatibility assessment of BMAP‐27B

Male BALB/c mice (6–8 weeks of age, 18 ± 2 g) were purchased from Tengxin Biotechnology Co., Ltd., for the animal studies. Healthy male BALB/c mice were randomly divided into four groups, with six mice per group. Mice in the three experimental groups received intraperitoneal injections of peptide solutions at doses of 5, 10, and 20 mg/kg, while the control group received injections of PBS. Injections were administered once daily for five consecutive days, and the body weight of each mouse was recorded daily. On the sixth day, the mice were euthanized under deep anesthesia and blood samples were collected for biochemical analysis, including AST, CREA, UREA, and ALT. The livers and kidneys were subsequently weighed, fixed in 4% paraformaldehyde, and subjected to histopathological examination using H&E staining.

### Assessment of in vivo activity of BMAP‐27B

Male BALB/c mice were used in this study, with six mice per group. The mice were infected via an intraperitoneal injection with a suspension of 1.0 × 10^8^ CFU of CRKP2. At 1 and 6 h postinfection, the mice were treated with PBS, BMAP‐27B at doses of 3, 6, or 9 mg/kg, MEM at 10 mg/kg alone, or a combination of BMAP‐27B (3 mg/kg) and MEM (10 mg/kg). After 24 h of bacterial challenge, the surviving mice were euthanized by cervical dislocation. The kidneys, livers, spleens, and lungs of each mouse were harvested within 48 h postinfection. A portion of these organs was taken for histologic analysis using H&E staining, while the remaining tissue samples were weighed, homogenized in sterile normal saline, and serially diluted in saline. A volume of 100 µl of the homogenate dilutions was uniformly plated onto agar plates containing MEM (8 μg/ml). After overnight incubation at 37°C, colonies were counted and the results were expressed as CFU/g.

### Molecular docking and ITC assay

The detailed steps of molecular docking are provided in the Supporting Information. Microcalorimetric experiments were conducted to examine the interaction between BMAP‐27B and the divalent cation zinc (Zn^2+^) using ITC to determine the binding affinity of BMAP‐27B for Zn^2+^. Both BMAP‐27B and Zn^2+^ were dissolved in PBS (pH 7.4). Before the experiment, all samples were degassed in a sonication bath for 10 min. At 25°C, Zn^2+^ was sequentially injected into the calorimetric cell containing BMAP‐27B, with a total of 25 injections performed, allowing for a 180‐s equilibration interval between injections. The resulting data were analyzed using software to calculate *K*_d_.

### Statistical analyses

The results are expressed as the mean ± SD and were analyzed using GraphPad Prism 8.0. Statistical significance was analyzed using Student's *t*‐test, with *p < 0.05, **p < 0.01, ***p < 0.001, and ****p < 0.0001 indicating significance levels.

## AUTHOR CONTRIBUTIONS

**Xiaoxiao Zhang**: Conceptualization; data curation; formal analysis; methodology; validation; visualization; writing—original draft; writing—review and editing. **Yongdong Li**: Writing—review and editing; data curation. **Lei Xu**: Investigation. **Zhe Chen**: Investigation. **Shengzhi Guo**: Investigation. **Jun Liao**: Investigation. **Min Ren**: Investigation. **Yao Wang**: Investigation. **Yi Chen**: Investigation. **Chuanxing Wan**: Investigation; conceptualization; visualization. **Jing Zhang**: Writing—review and editing. **Xihui Shen**: Conceptualization; funding acquisition; resources; writing—review and editing.

## ETHICS STATEMENT

All procedures involving animals were approved by the Ethics Committee of Northwest A&F University (Shaanxi, China), approval number XN2023‐1004.

## CONFLICT OF INTERESTS

The authors declare no conflict of interests.

## Supporting information

Figure S1.

Figure S2.

Figure S3.

Figure S4.

Figure S5.

Figure S6.

FigureS7.

Figure S8.

Supplementary information.

## Data Availability

The data generated and analyzed during this study are available from the corresponding author upon reasonable request.
