# Supplementary figures and images for: The broad‐spectrum antimicrobial peptide BMAP‐27B potentiates carbapenems against NDM‐producing pathogens in food animals
Source: mLife. 2025 Jun 24;4(3):275–93. doi: 10.1002/mlf2.70020 (PMC12207908; doi:10.1002/mlf2.70020)

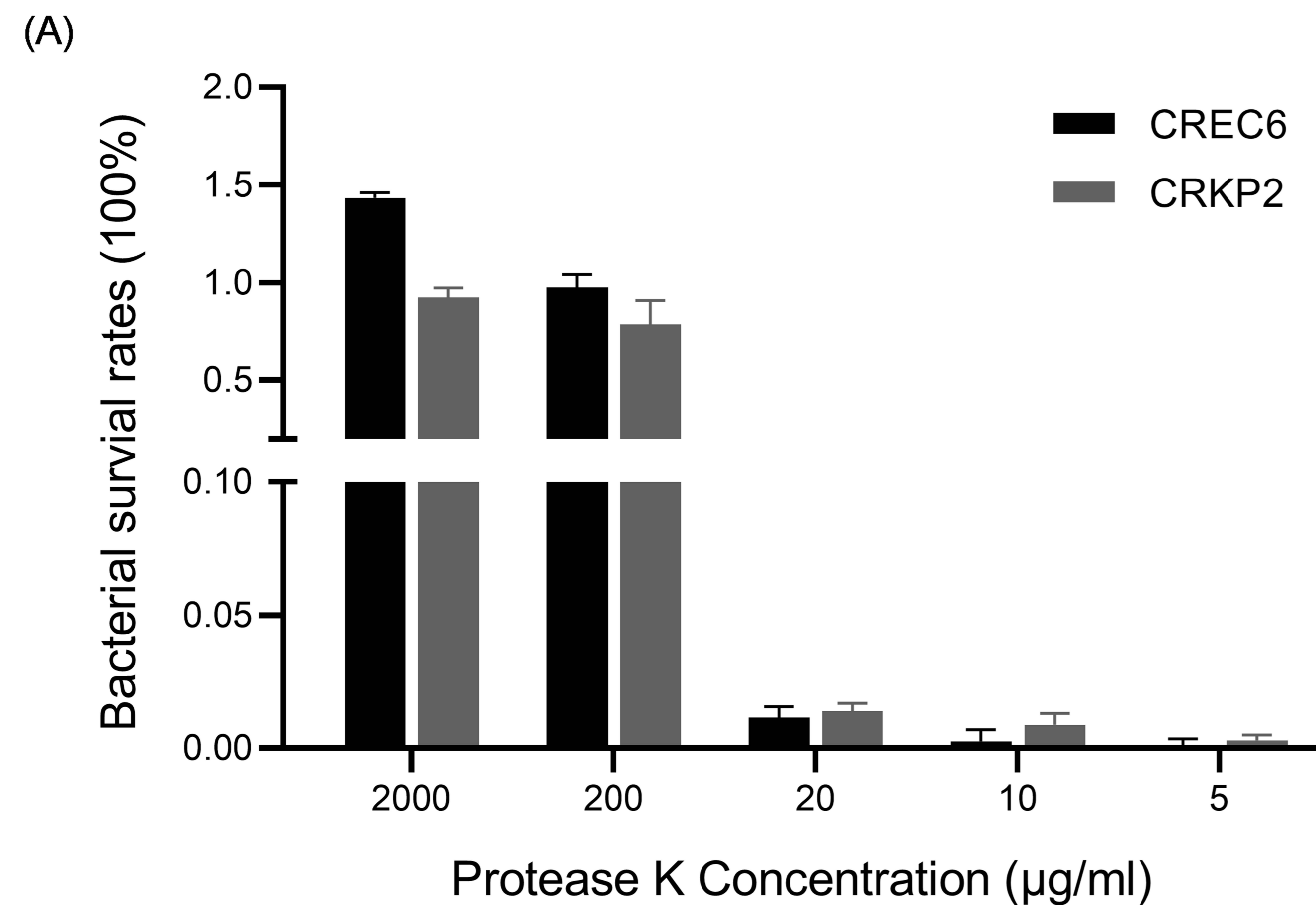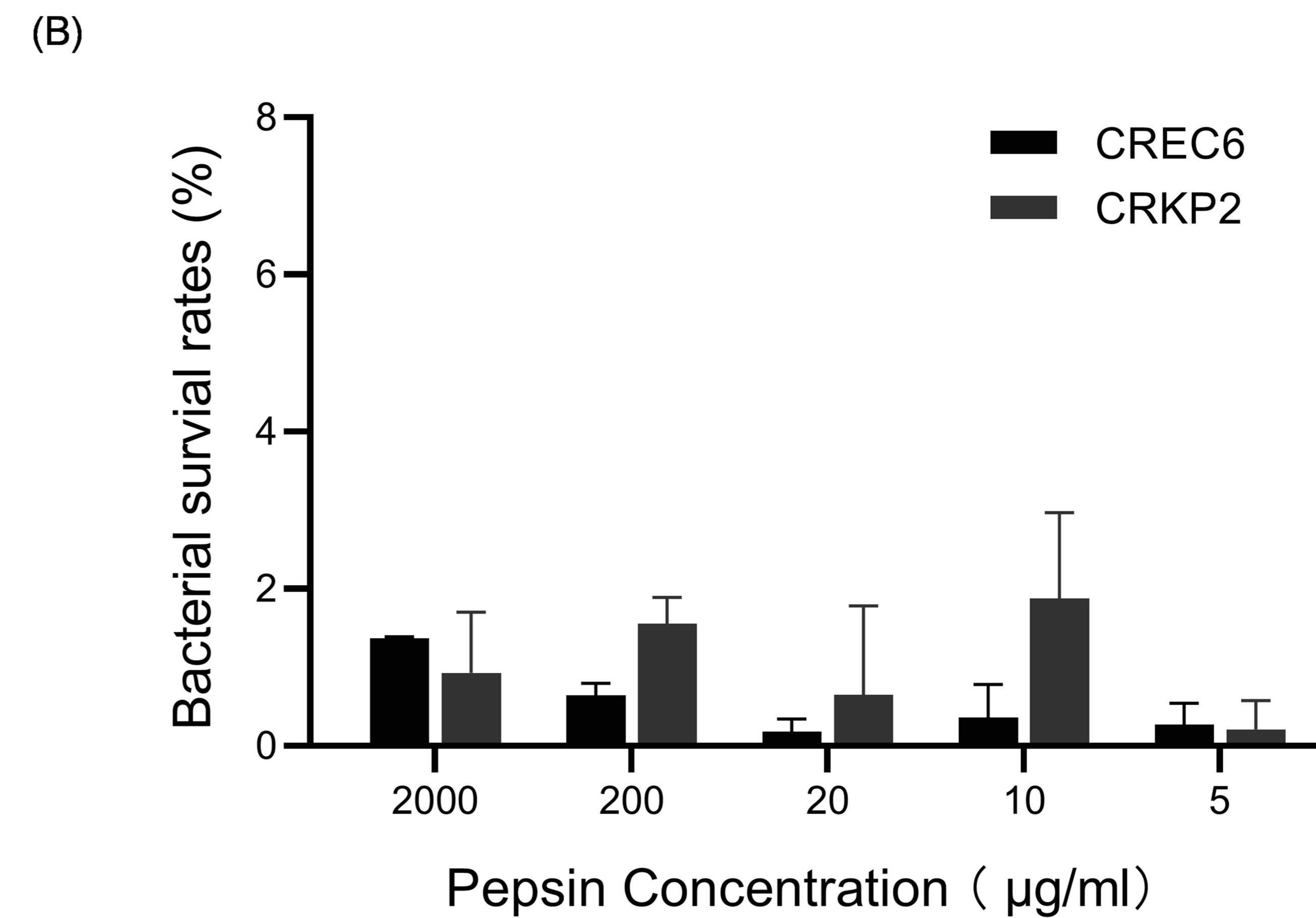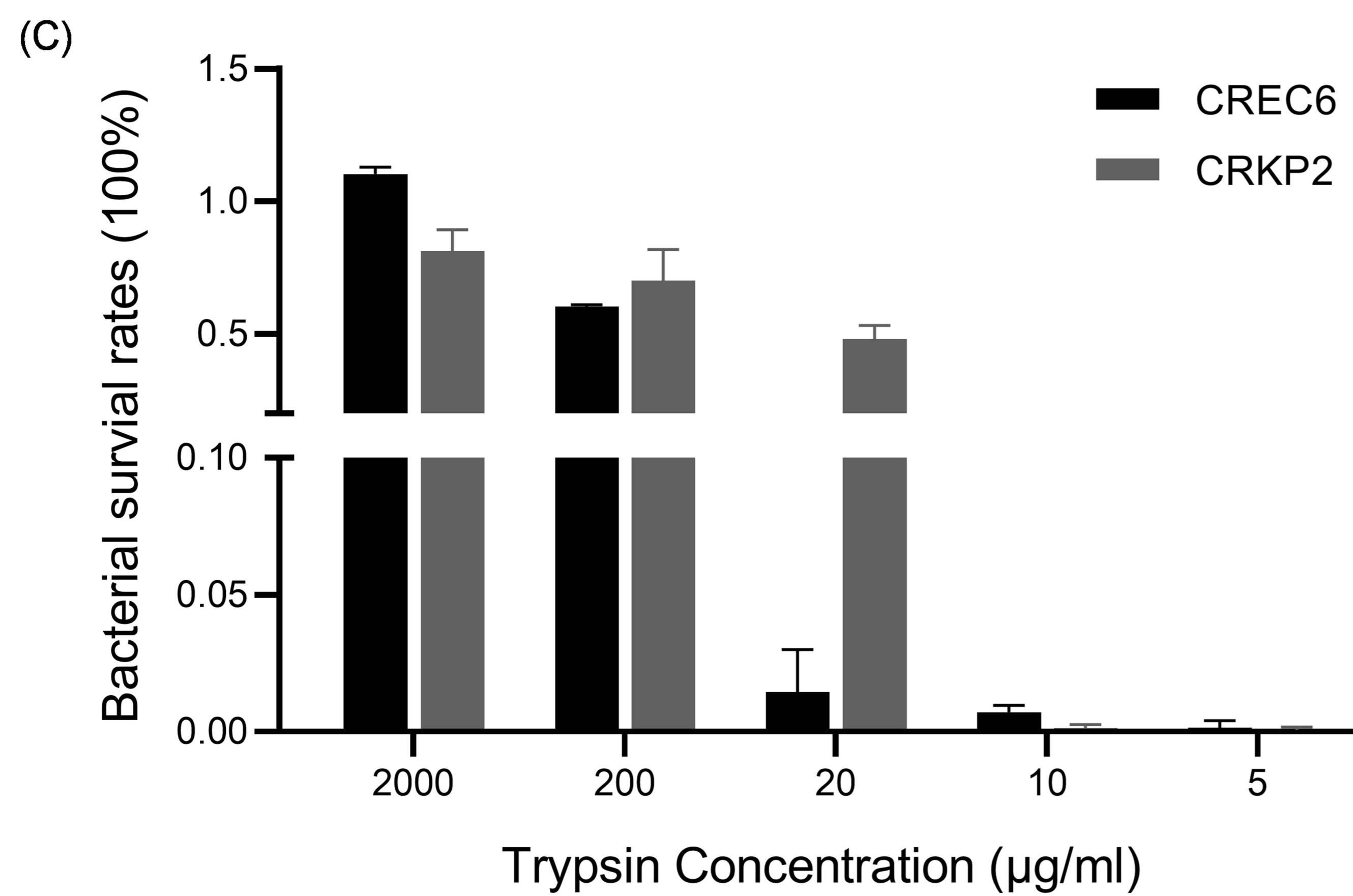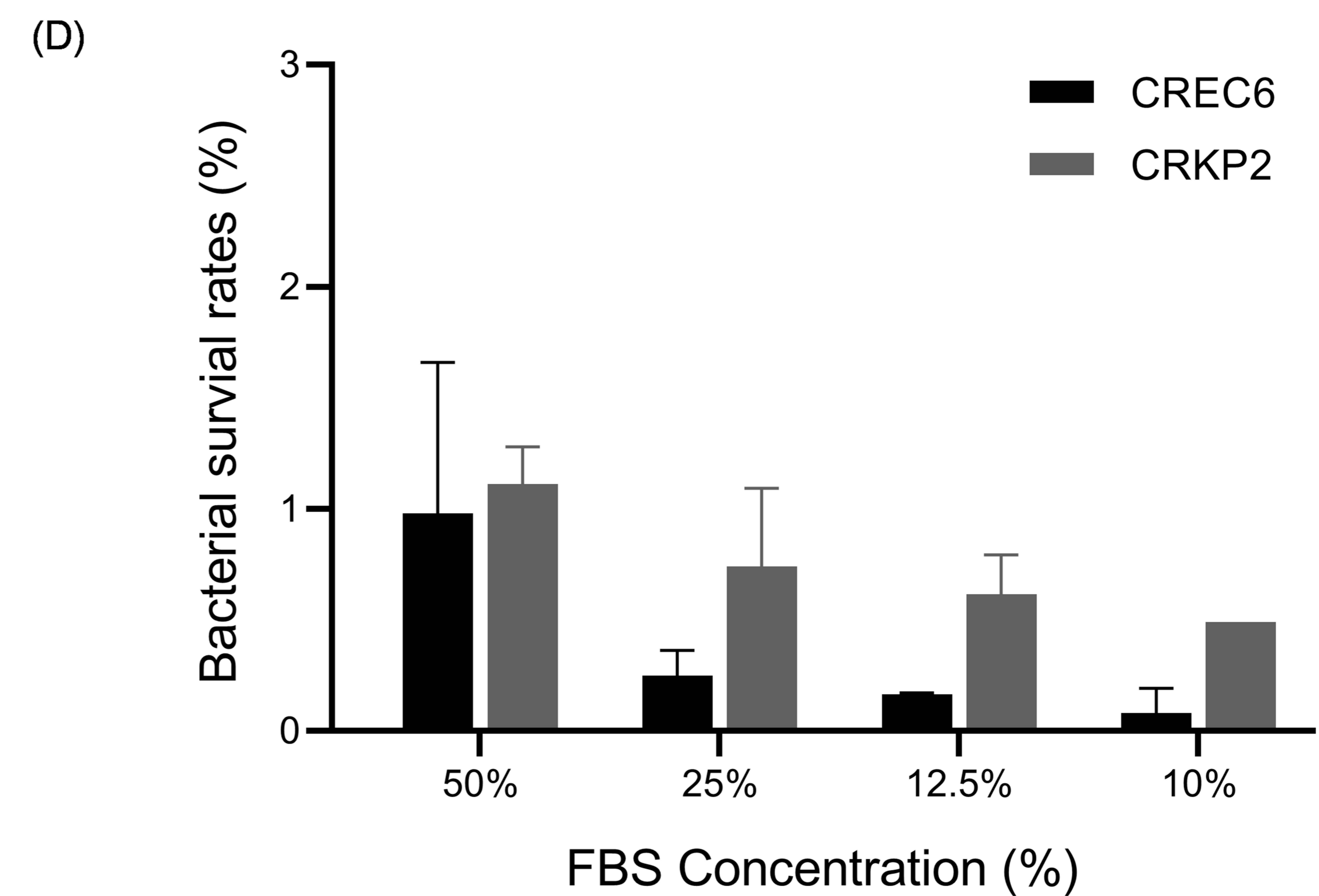

Supplement: Supplementary file 1 — Figure S1. [file MLF2-4-275-s006.pdf]

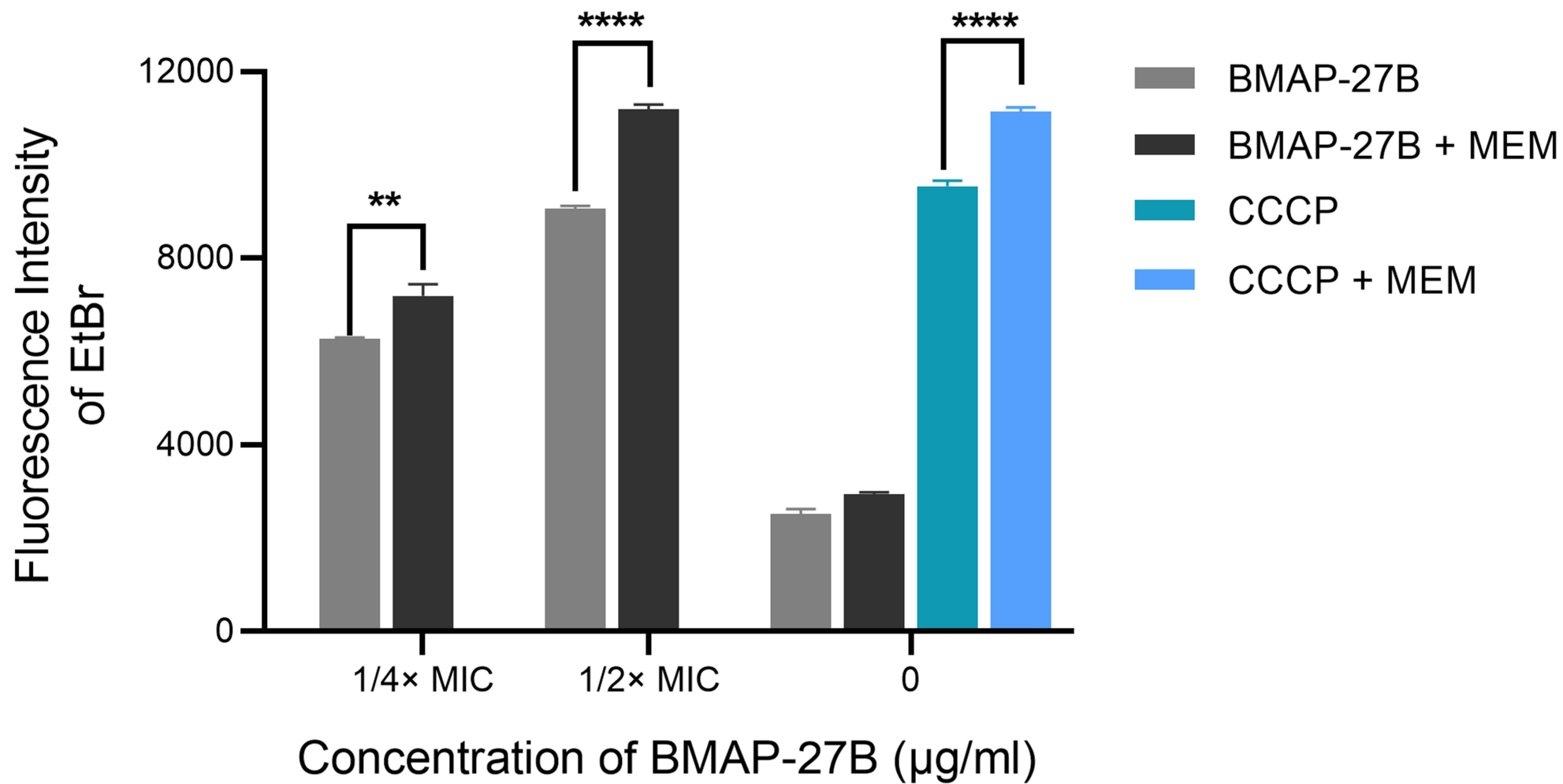

Supplement: Supplementary file 2 — Figure S2. [file MLF2-4-275-s002.pdf]

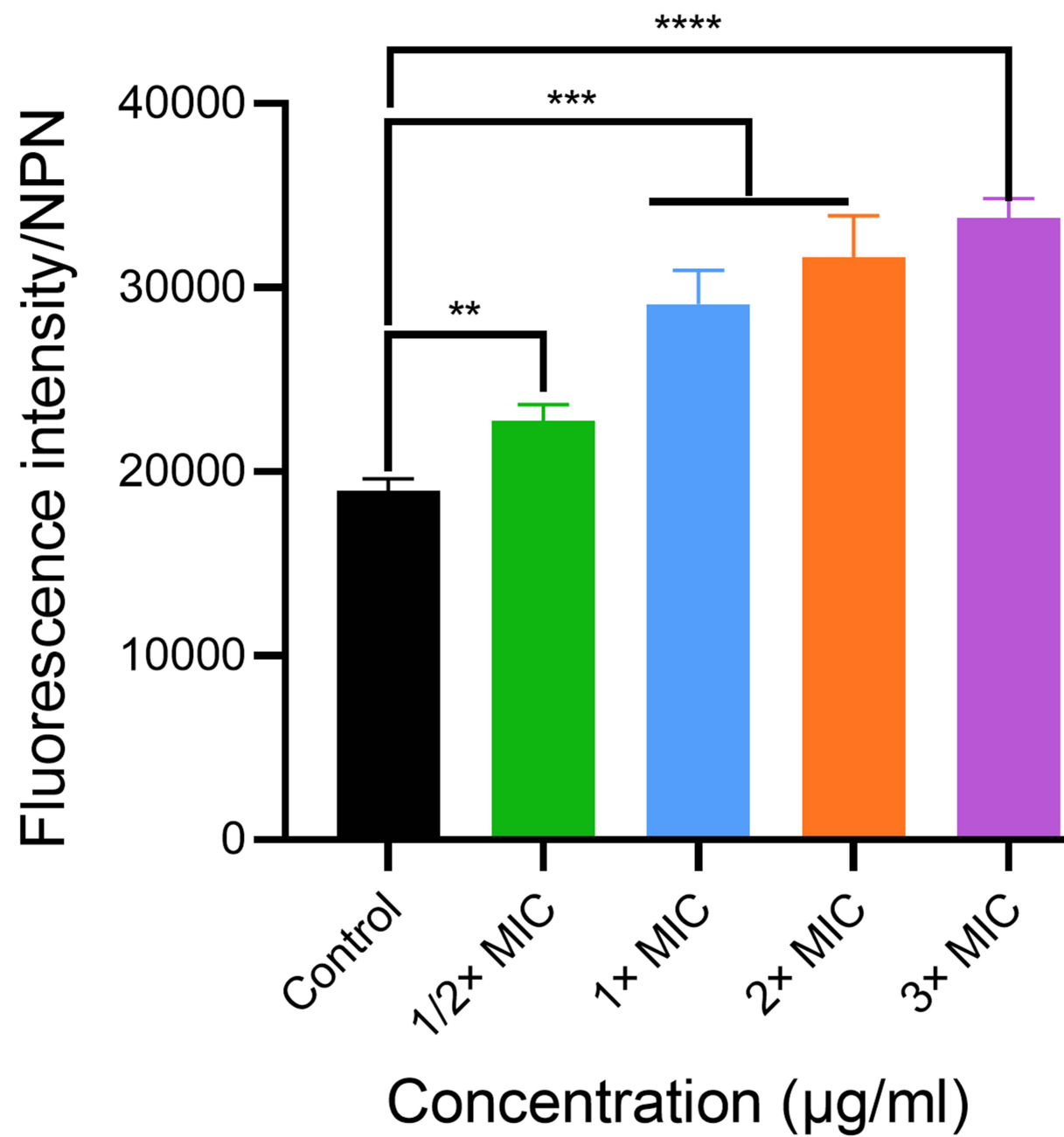

Supplement: Supplementary file 3 — Figure S3. [file MLF2-4-275-s008.pdf]

PI

Bright field

Merge

CRAB1

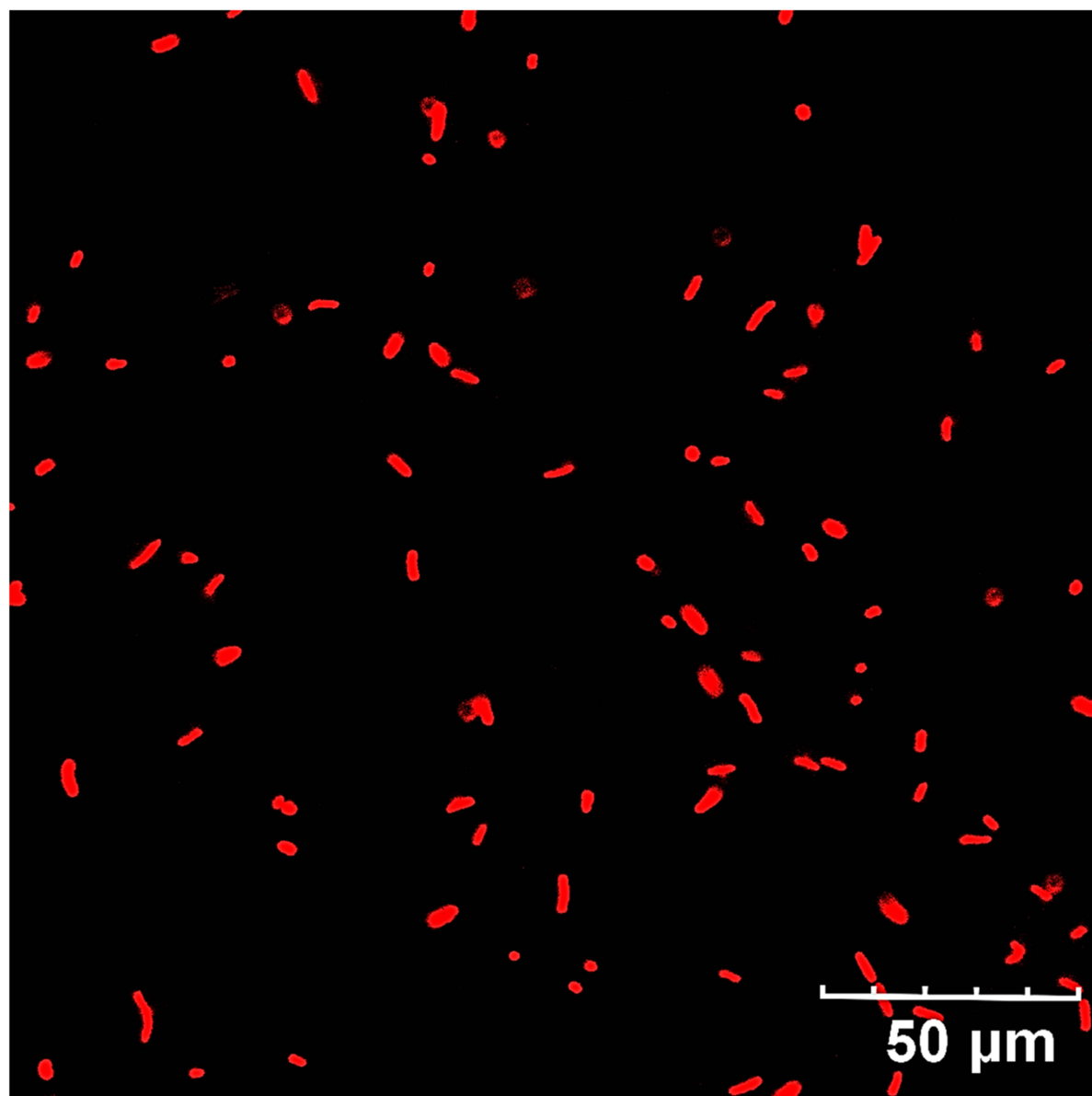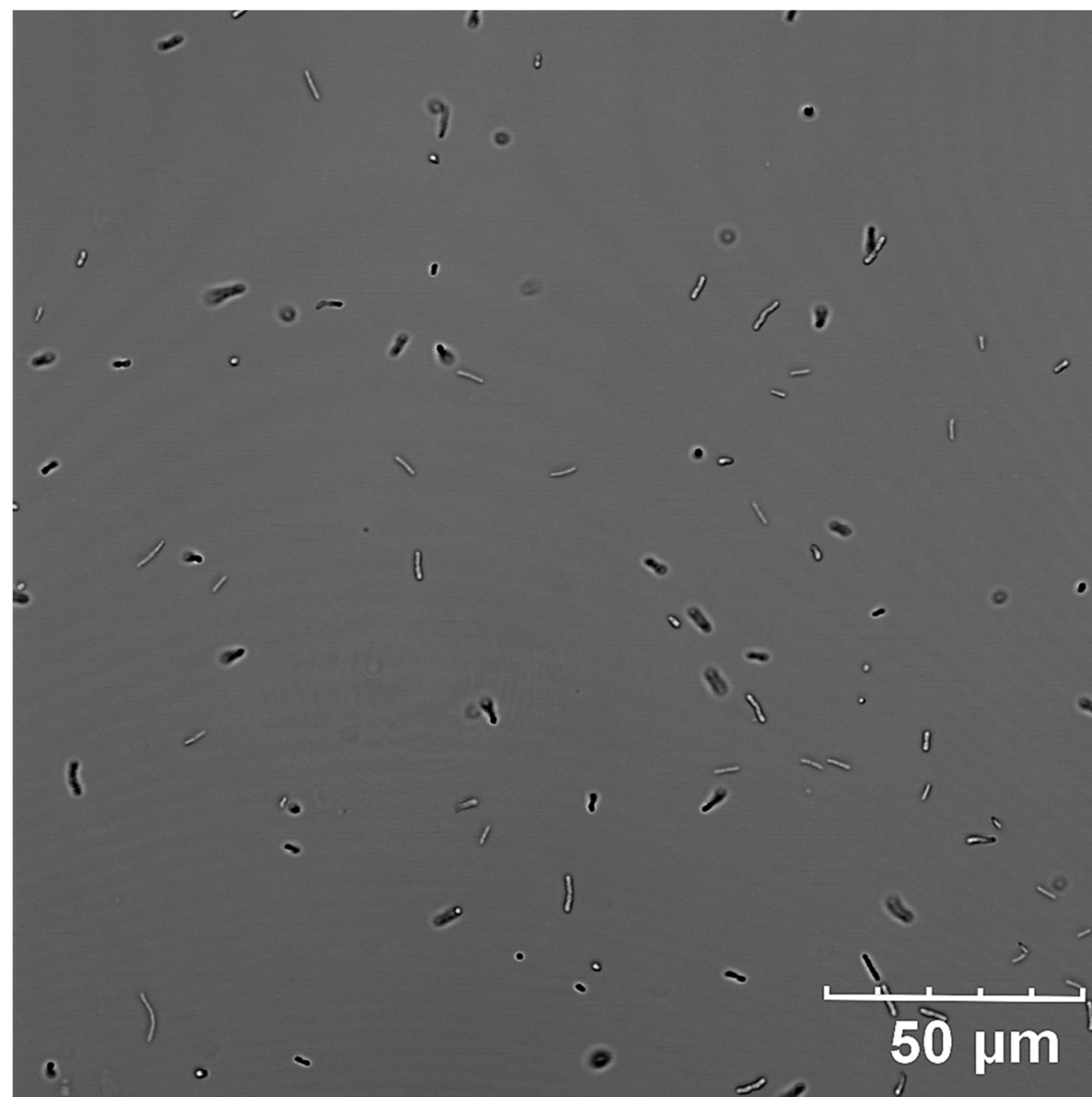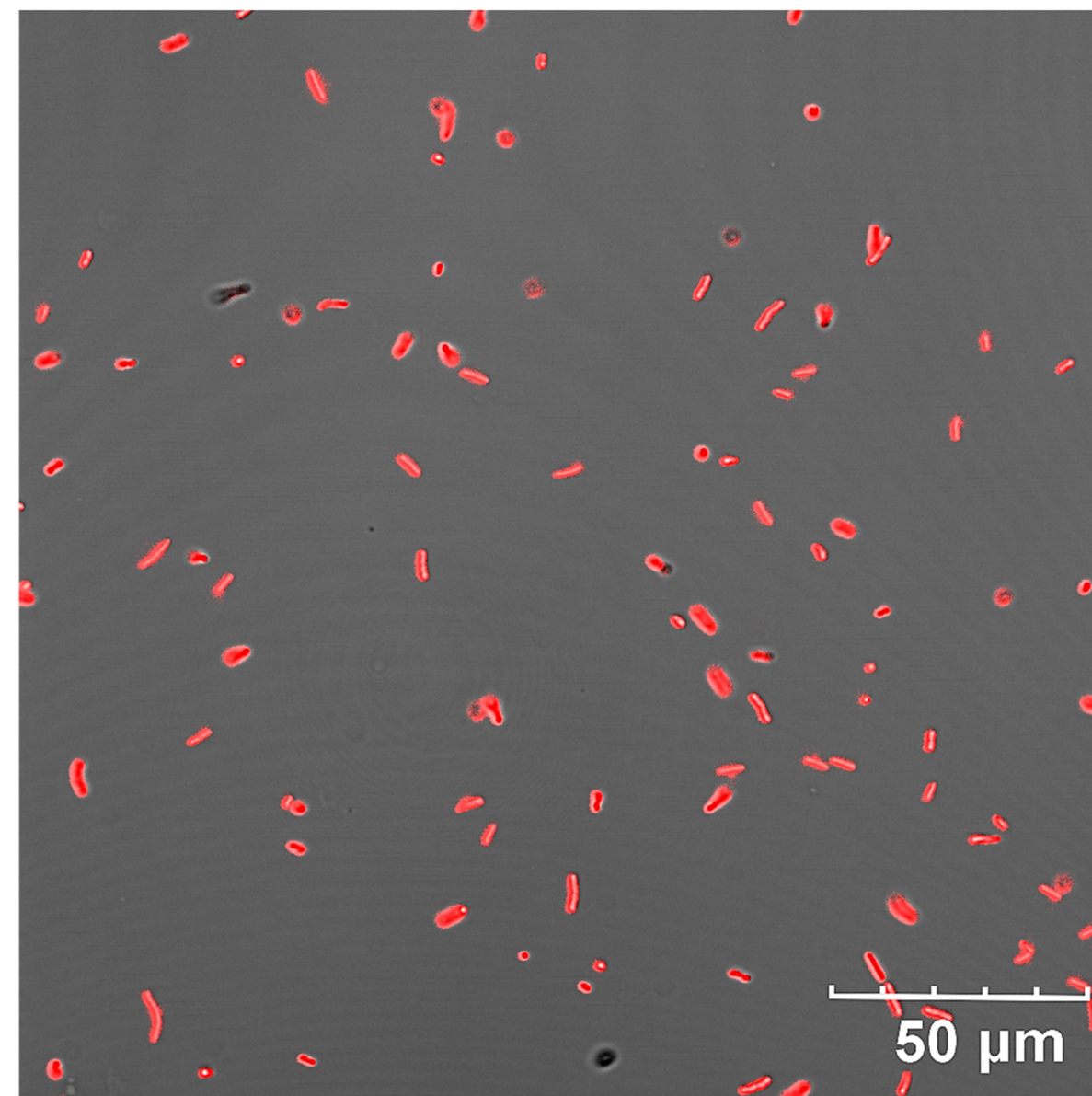

SL1344

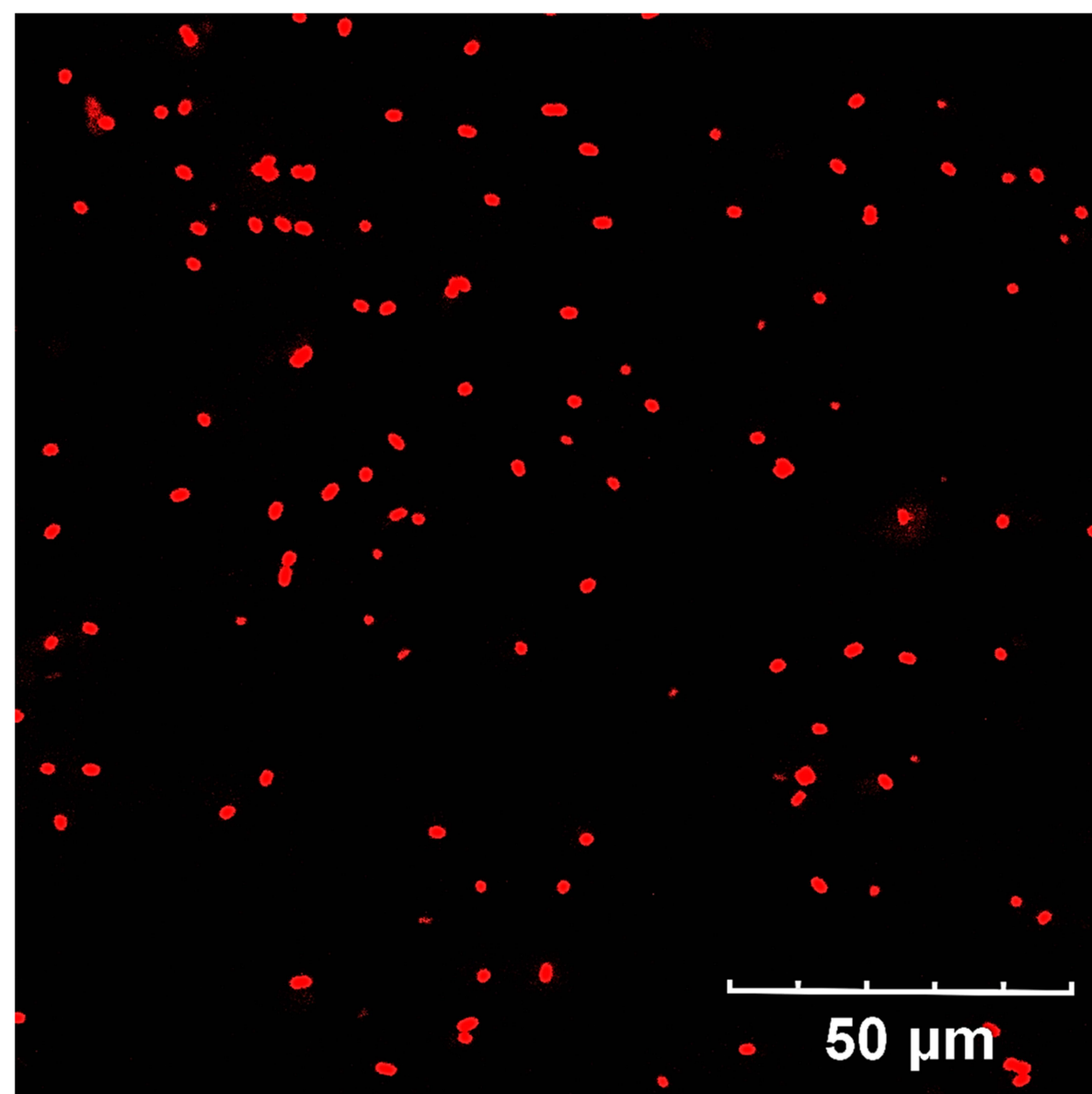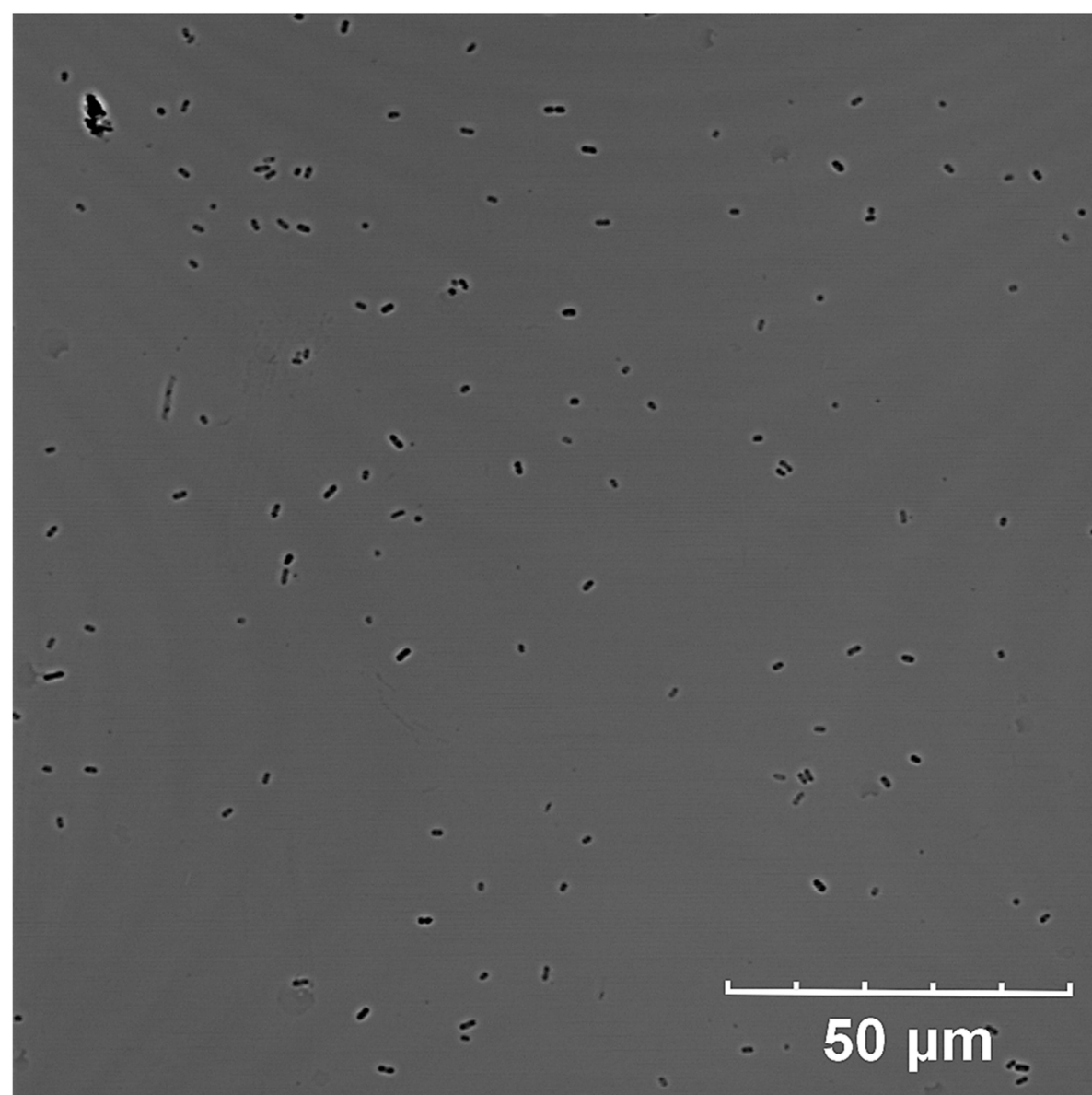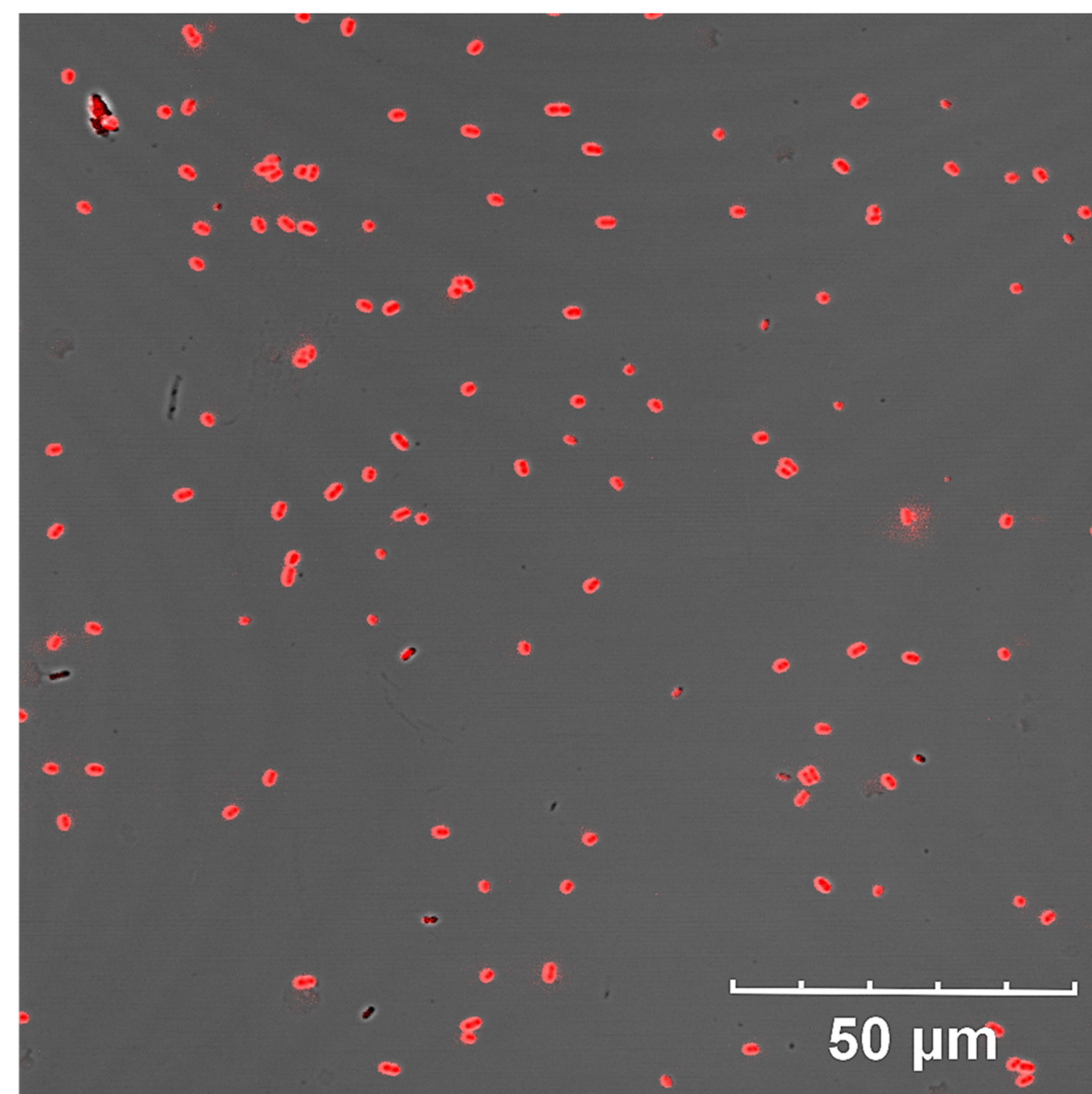

CRCB1

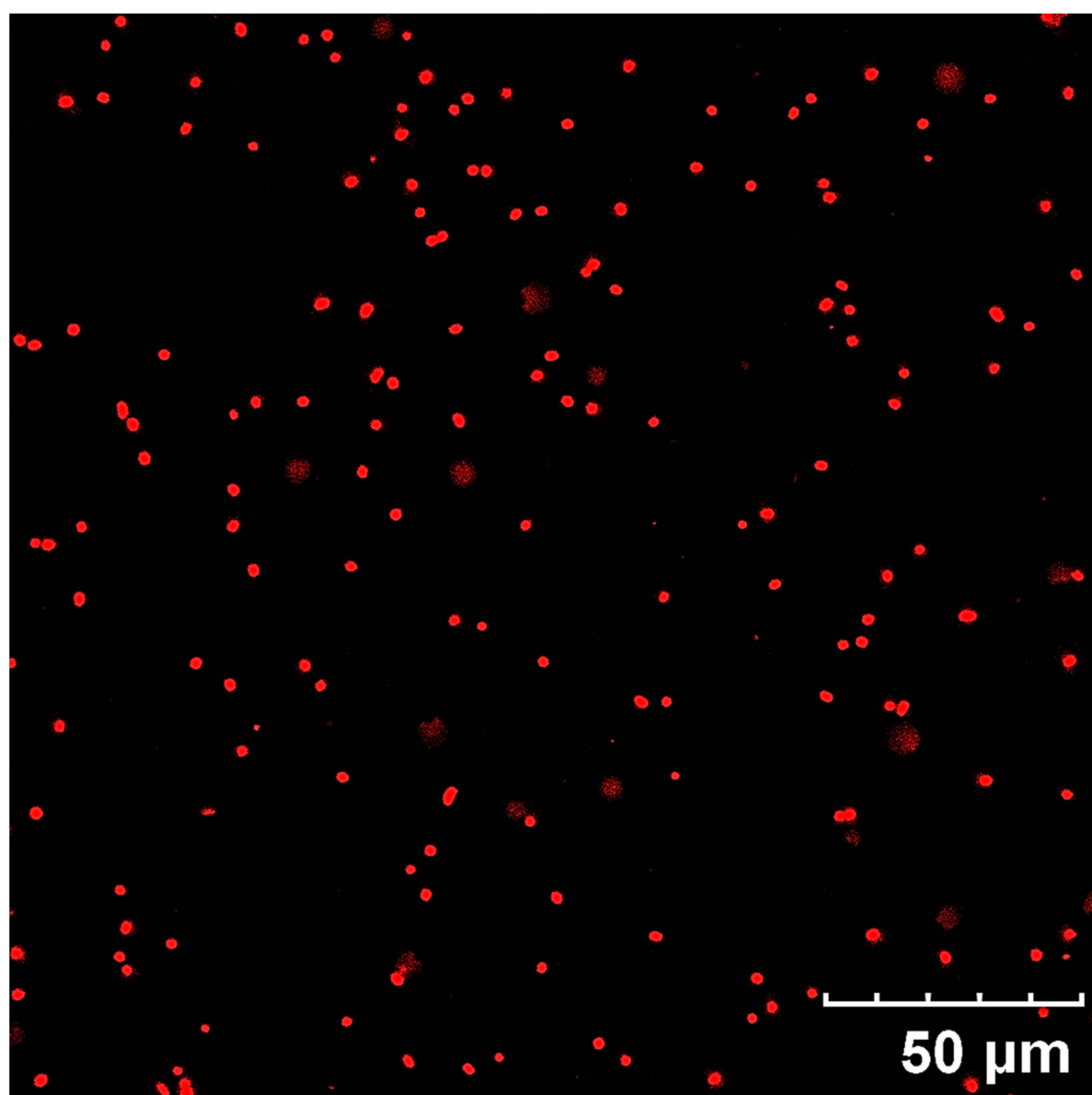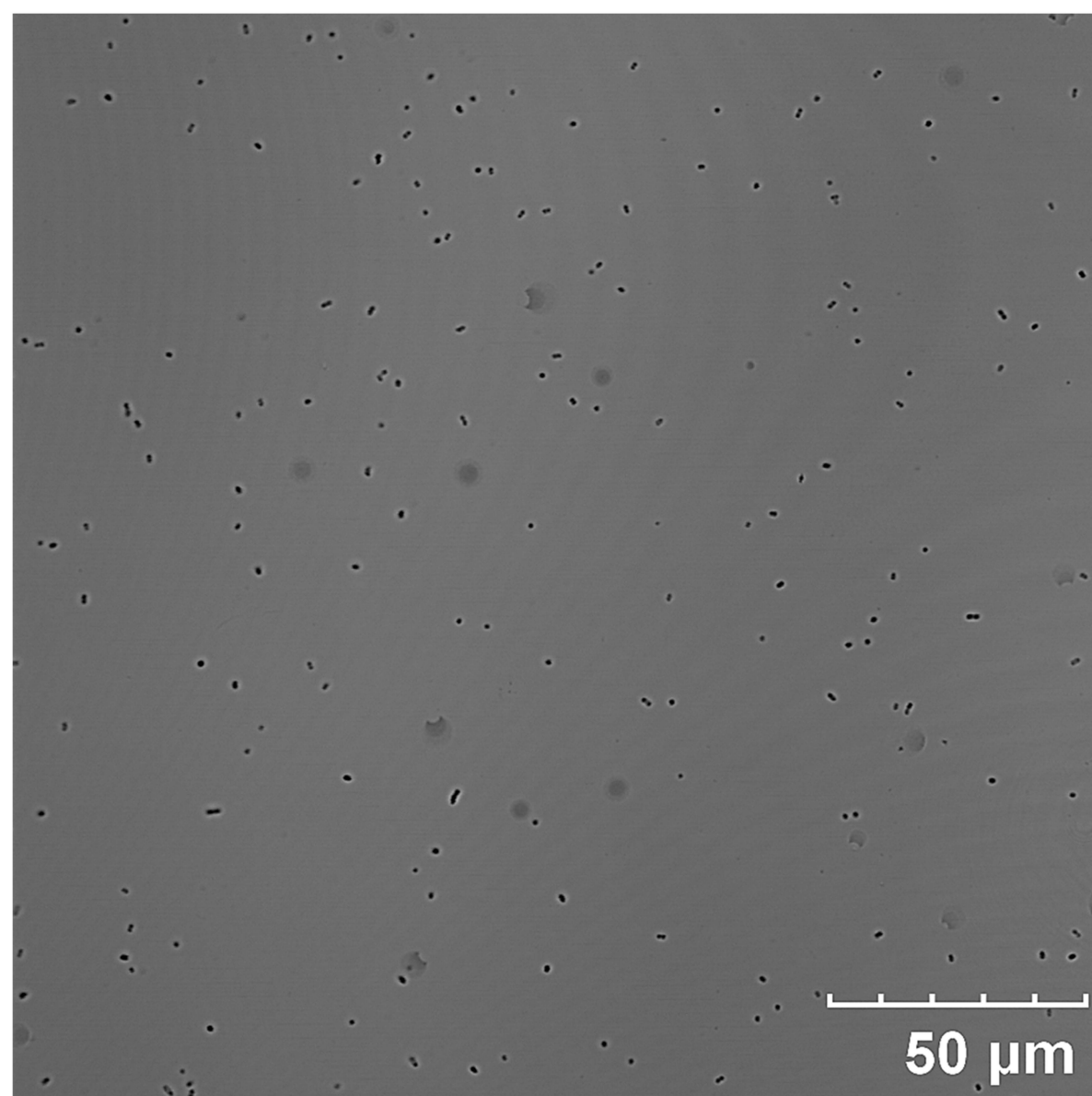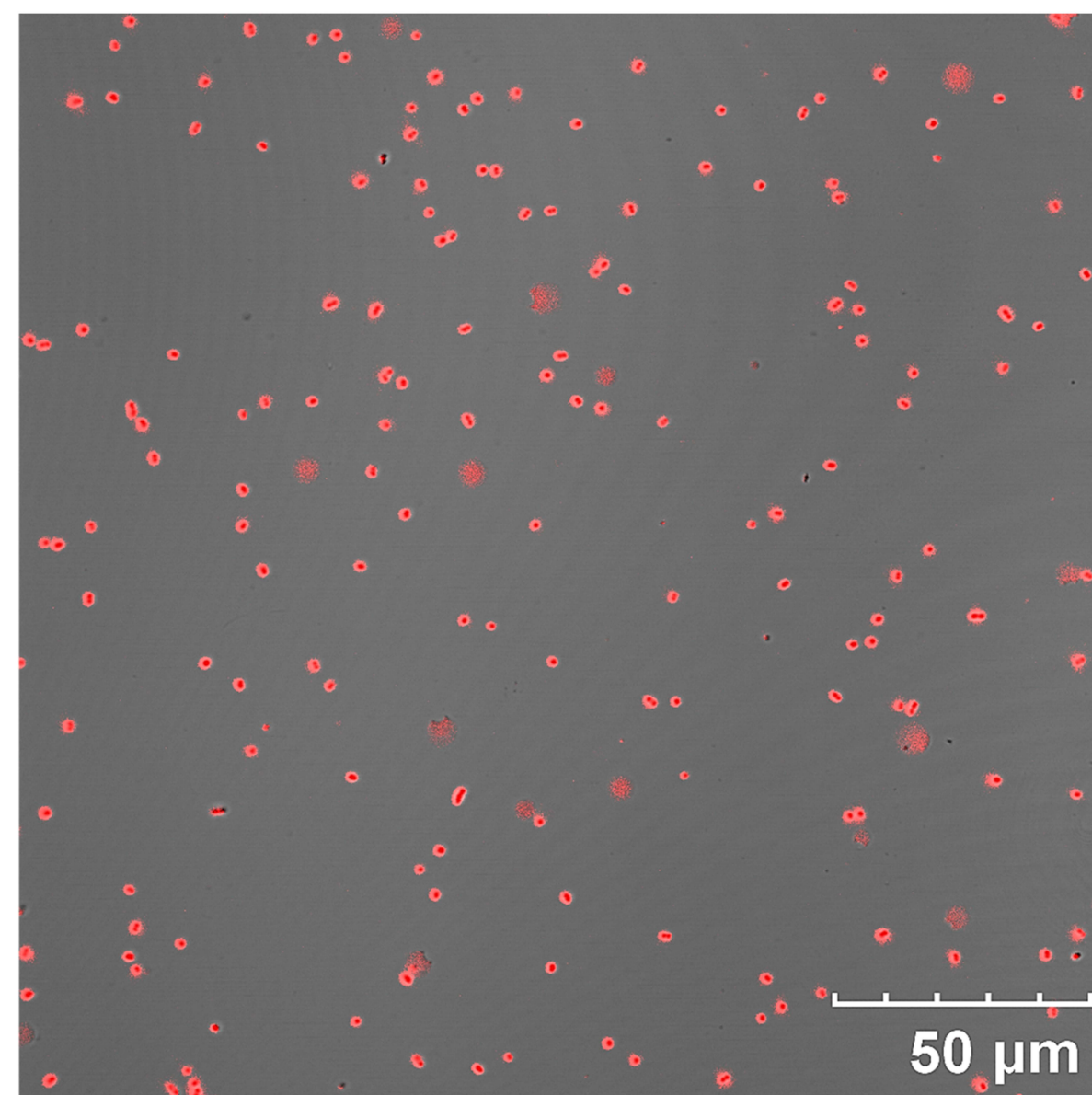

Supplement: Supplementary file 4 — Figure S4. [file MLF2-4-275-s007.pdf]

## Extracellular ATP

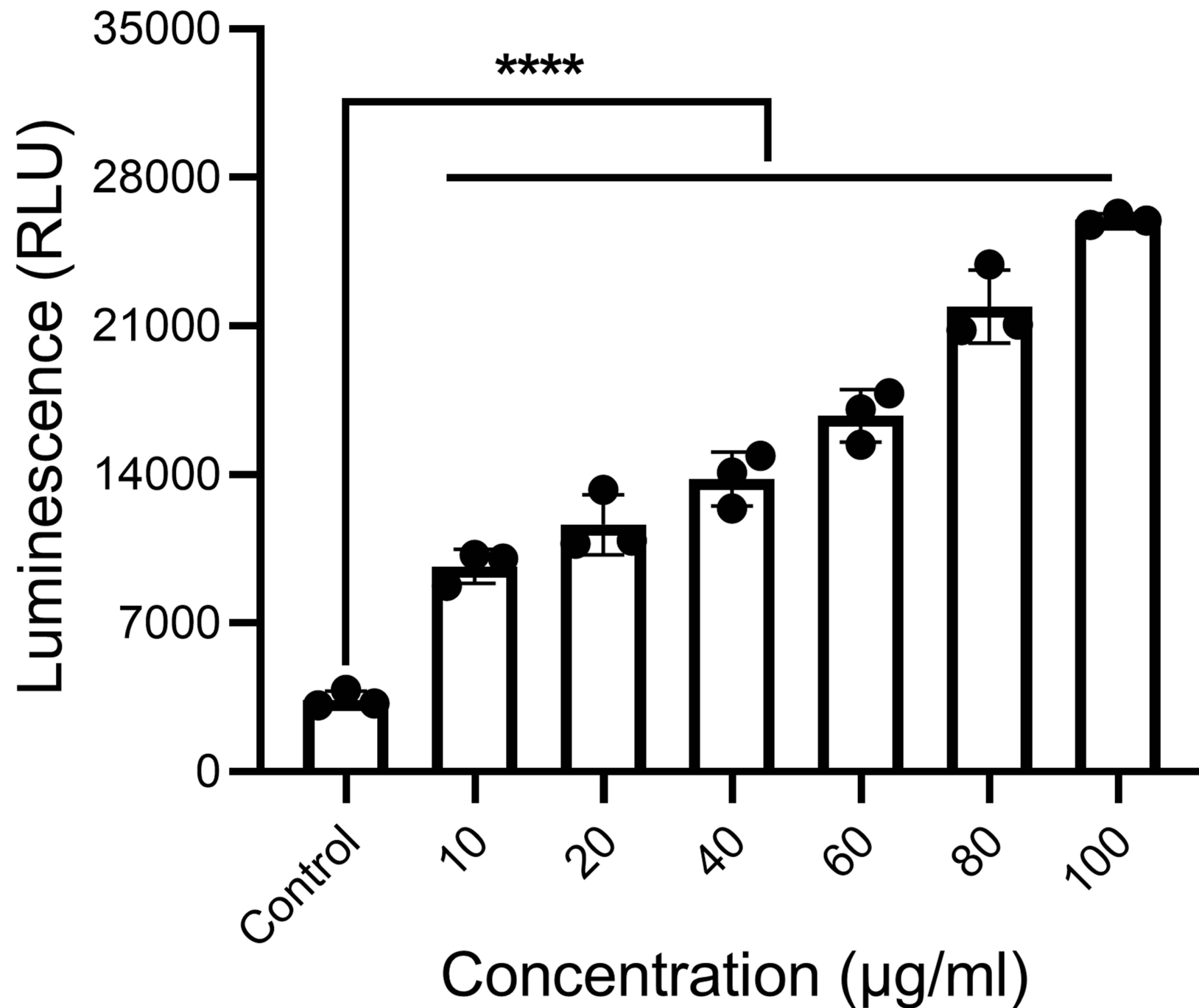

Supplement: Supplementary file 5 — Figure S5. [file MLF2-4-275-s001.pdf]

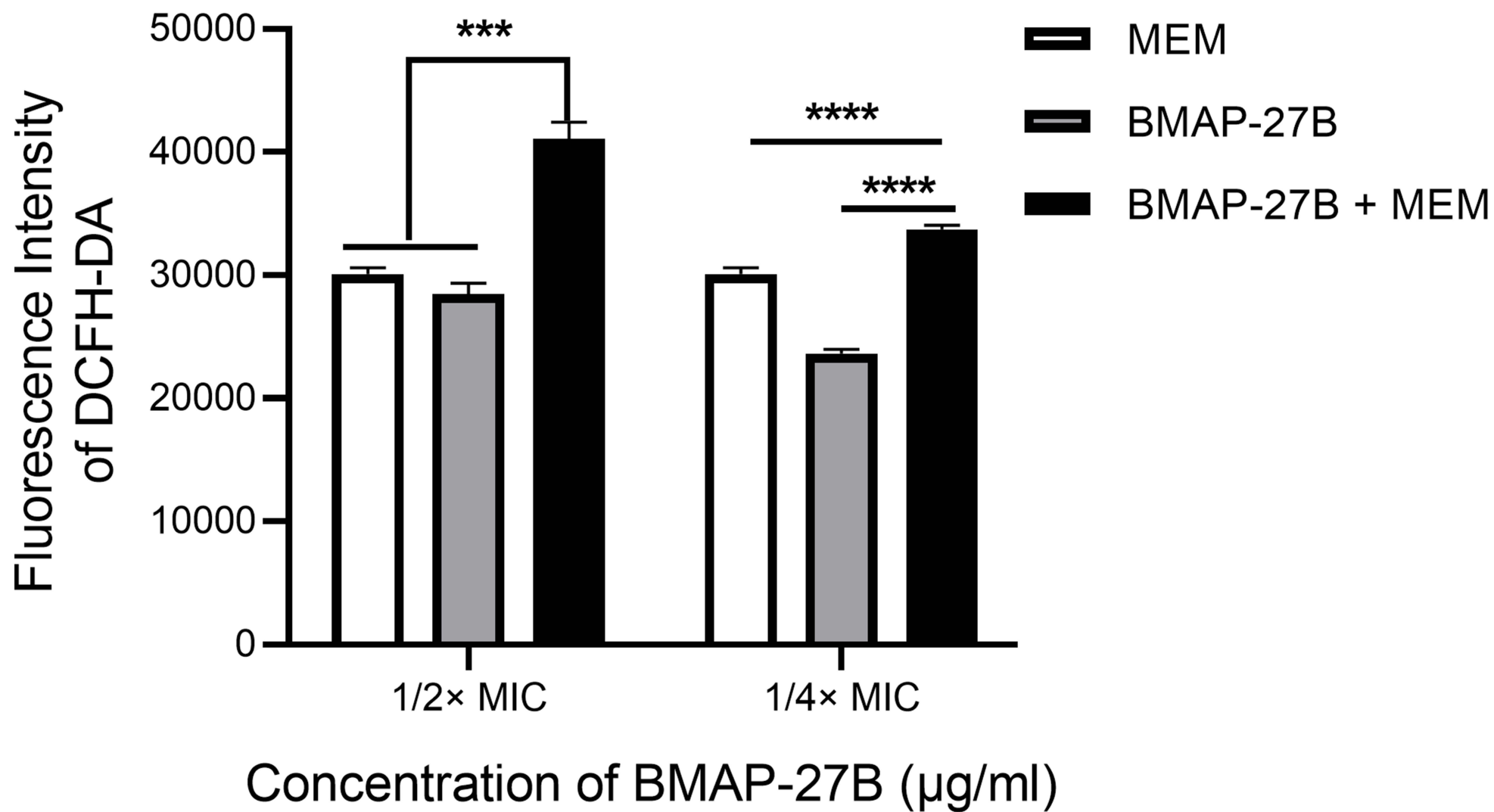

Supplement: Supplementary file 6 — Figure S6. [file MLF2-4-275-s004.pdf]

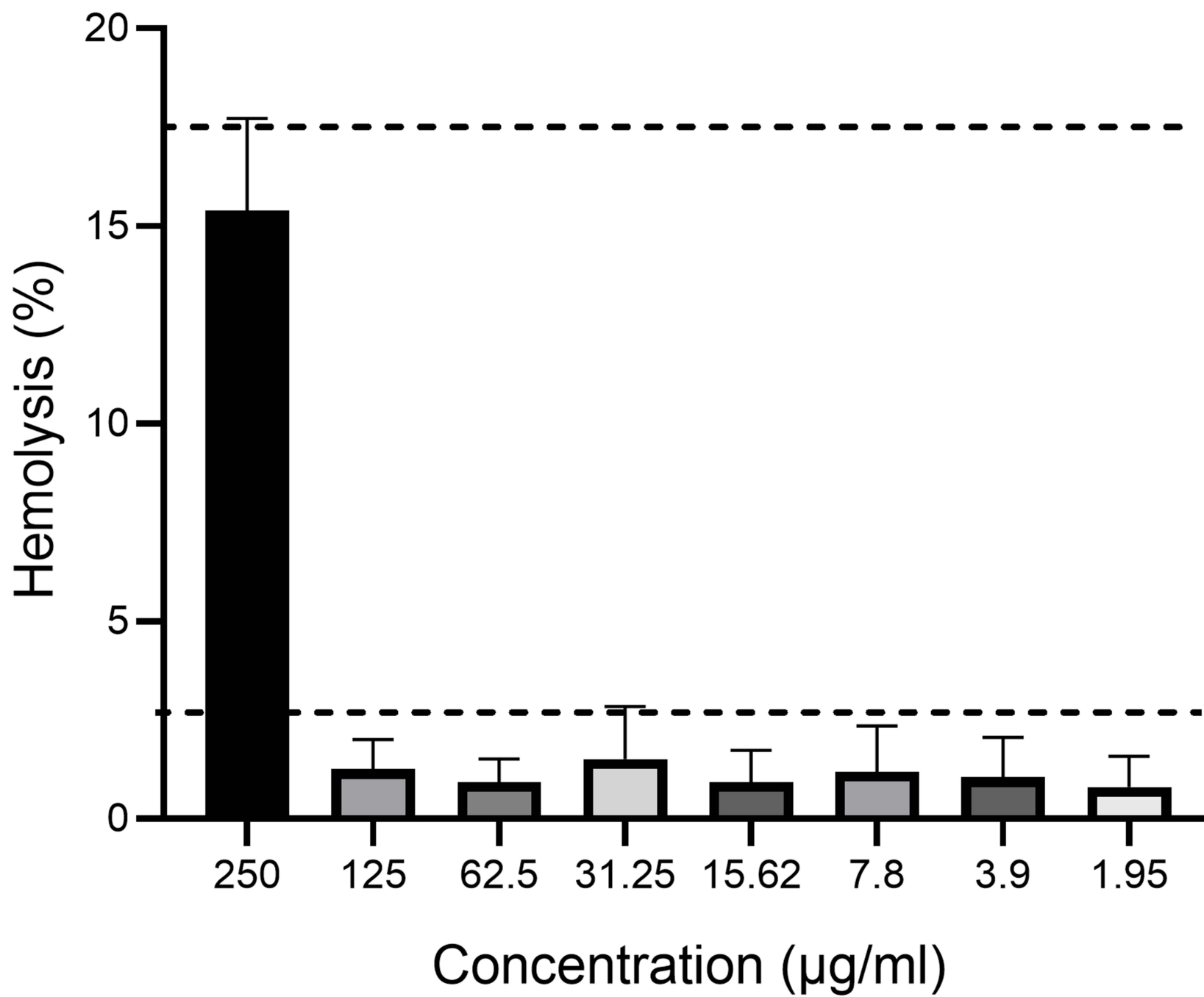

Supplement: Supplementary file 7 — FigureS7. [file MLF2-4-275-s009.pdf]

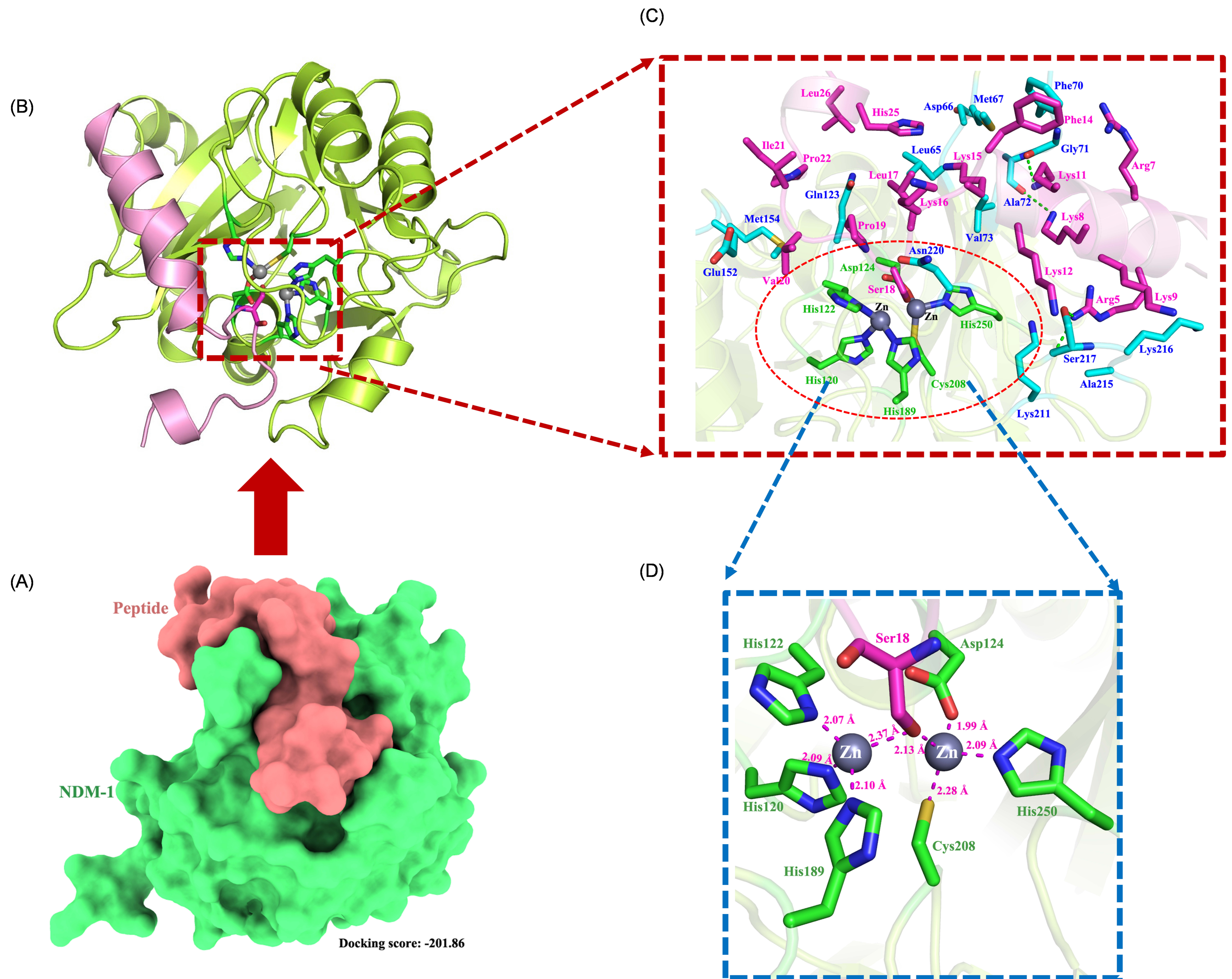

Supplement: Supplementary file 8 — Figure S8. [file MLF2-4-275-s005.pdf]
