# Supplementary material for: The broad‐spectrum antimicrobial peptide BMAP‐27B potentiates carbapenems against NDM‐producing pathogens in food animals
Source: mLife. 2025 Jun 24;4(3):275–93. doi: 10.1002/mlf2.70020 (PMC12207908; doi:10.1002/mlf2.70020)
Supplement: Supplementary file 9 — Supplementary information. [file MLF2-4-275-s003.docx]

**The Broad-Spectrum Antimicrobial Peptide BMAP-27B Potentiates Carbapenems against NDMs‐Producing Pathogens** **in Food Animals**

Xiaoxiao Zhang^a#^, Yongdong Li^b#^, Lei Xu^a^, Zhe Chen^c^, Shengzhi Guo^a^, Jun Liao^a^, Min Ren^c^, Yao Wang^a^, Yi Chen^b^, Chuanxing Wan^c^, Jing Zhang^c^*, Xihui Shen^a^*

^a^ State Key Laboratory for Crop Stress Resistance and High-Efficiency Production, Shaanxi Key Laboratory of Agricultural and Environmental Microbiology, College of Life Sciences, Northwest A&F University, Yangling, China.

^b^ Ningbo Municipal Center for Disease Control and Prevention, Ningbo, Zhejiang 315010, P. R. China;

^c^ College of Life Sciences, Tarim University, Alar, Xinjiang, China.

^#^ These authors contributed equally to this work.

* Author to whom correspondence should be addressed

E-mail: xihuishen@nwsuaf.edu.cn; zhang.jing@taru.edu.cn

Phone: 18966833023


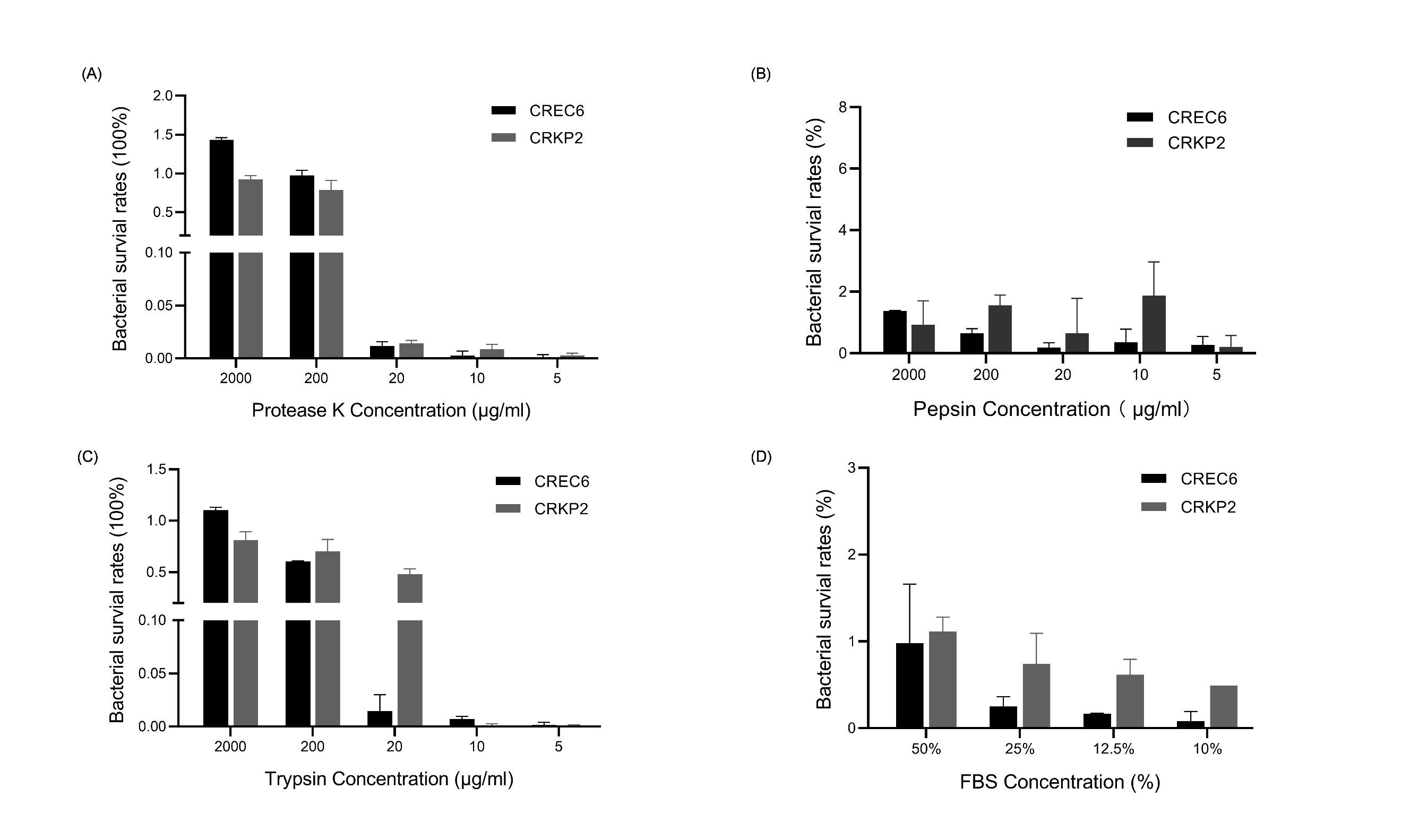
Supporting Information

Supplementary 1: Figures


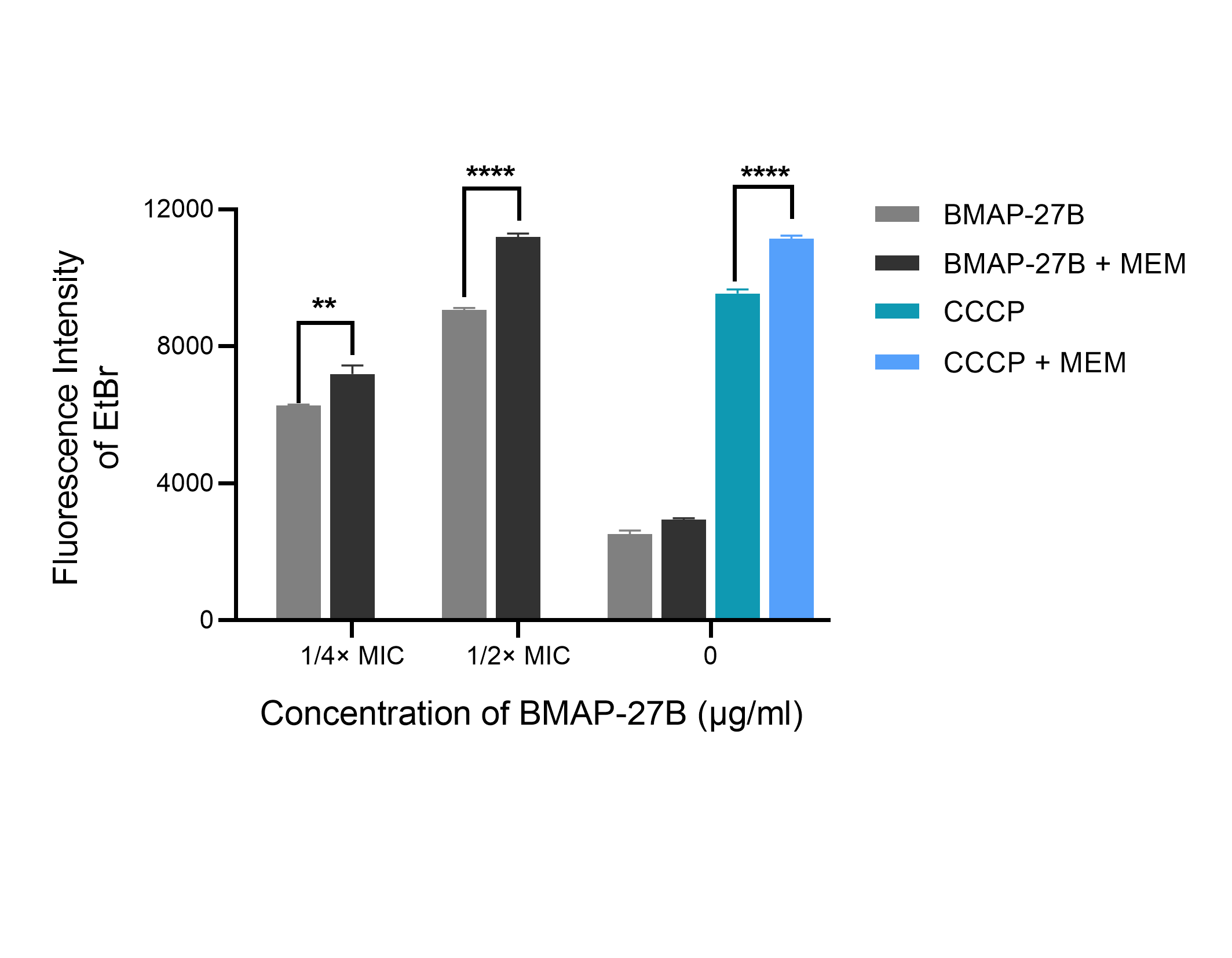
**Figure S1** Antibacterial activity of BMAP-27B at different concentrations of **(A)** protease K, **(B)** pepsin, **(C)** trypsin, and **(D)** serum. Data are expressed as mean values ± SD, based on three biologically independent samples. Statistical significance was analyzed by unpaired Student's t‐test (**p* < 0.05, ***p* < 0.01, ****p*< 0.001, *****p* < 0.0001).

**Figure S2** Inhibitory effect of BMAP-27B on the CRKP2 efflux pump. The accumulation of EtBr was measured to assess the activity of efflux pumps induced by BMAP-27B, meropenem, or their combination. Data are expressed as mean values ± SD, based on three biologically independent samples. Statistical significance was analyzed by unpaired Student's t‐test (**p* < 0.05, ***p* < 0.01, ****p*< 0.001,
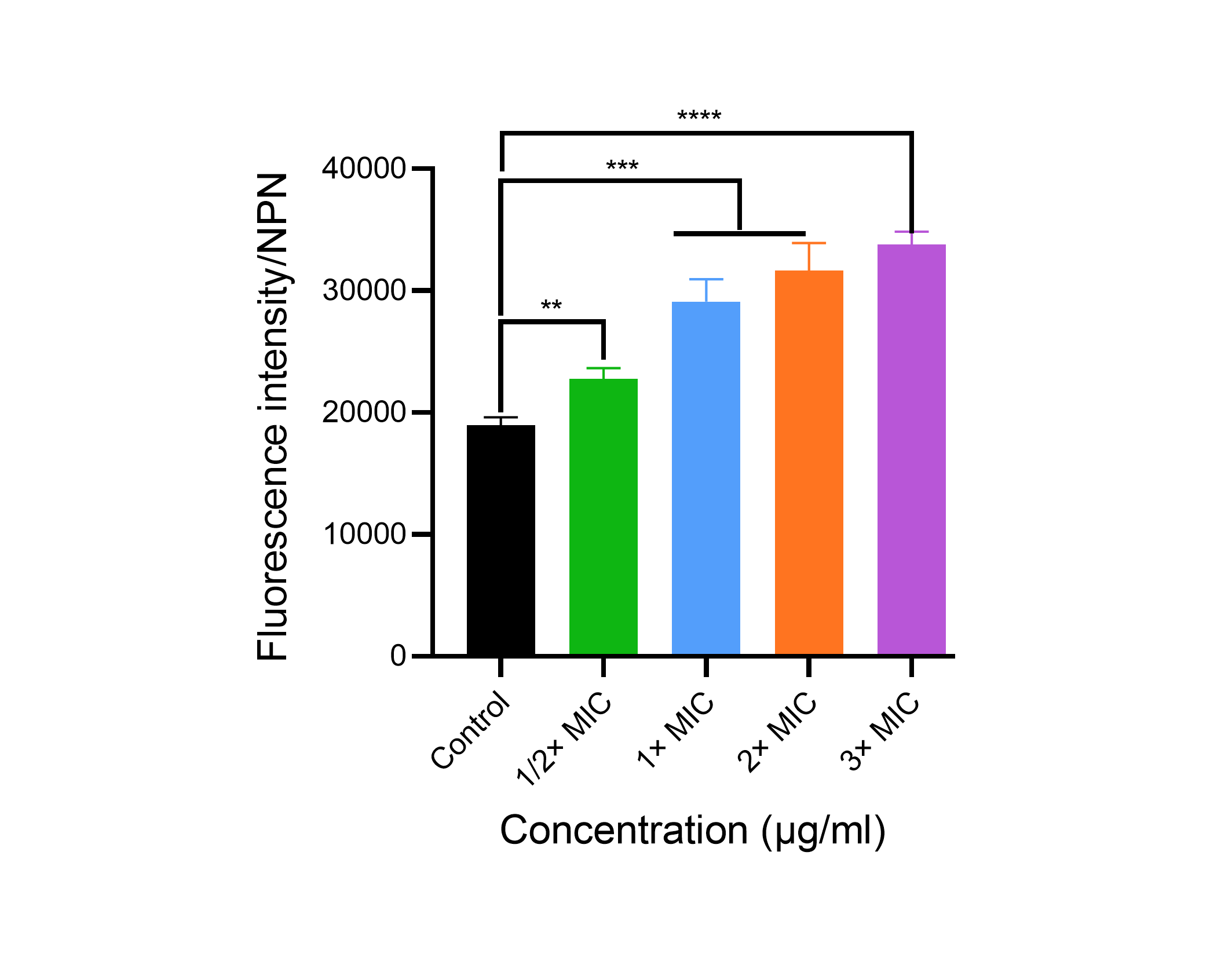
*****p* < 0.0001).

**Figure S3** Outer membrane permeabilization of CRKP2 after treatment with different concentrations and durations of BMAP-27B for 1 hour. Data are expressed as mean values ± SD, based on three biologically independent samples. Statistical significance was analyzed by unpaired Student's t‐test (**p* < 0.05, ***p* < 0.01, ****p*< 0.001, *****p* < 0.0001).


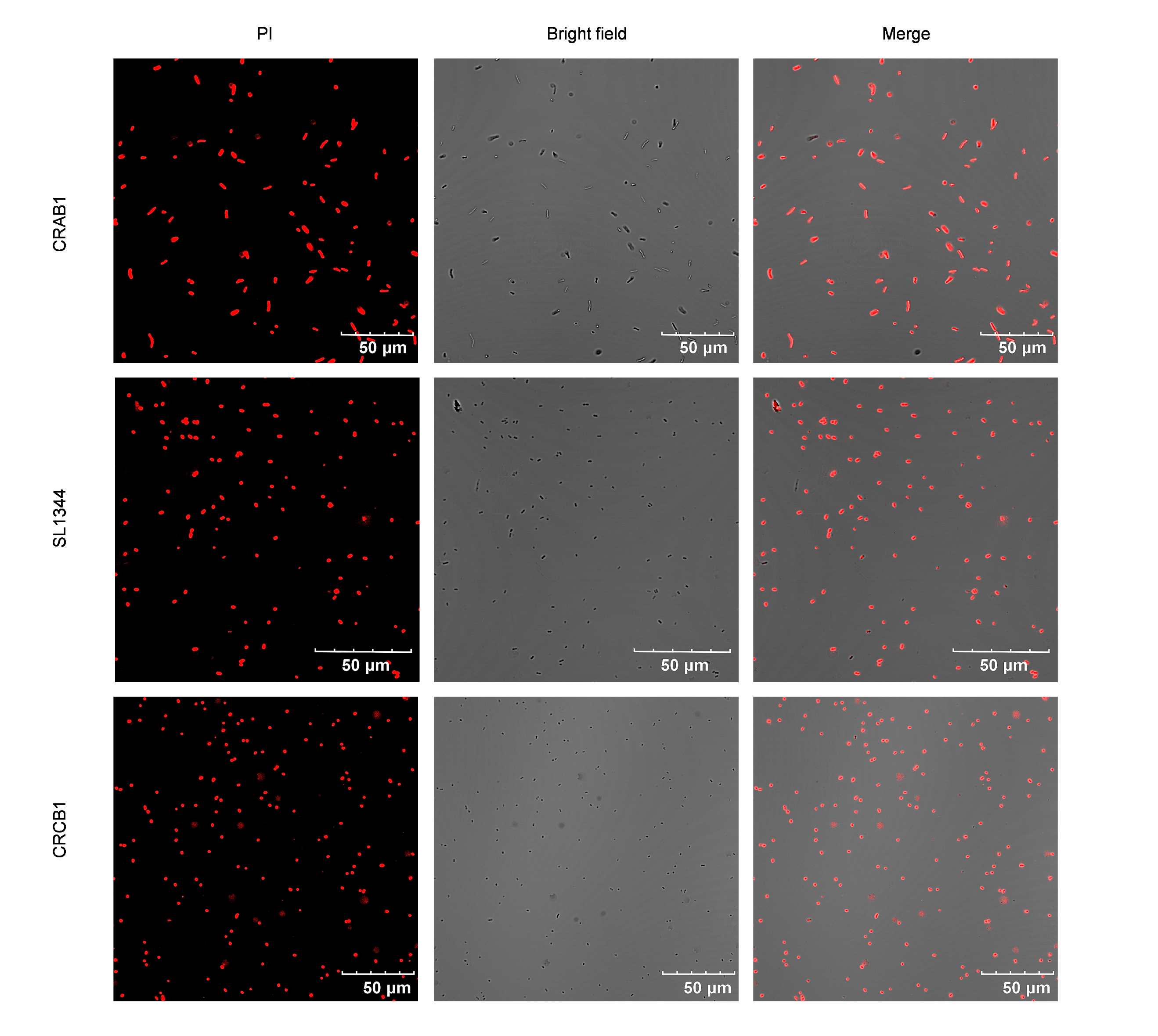
**Figure** **S4** Confocal images of CRAB1, *Salmonella* SL1344, and CRCB1 under the treatment of 1× MIC BMAP-27B at 37 °C for 1 h. Bright field represents viable bacterial cells, whereas dead cells were stained red by PI. Scale bar = 50 µm


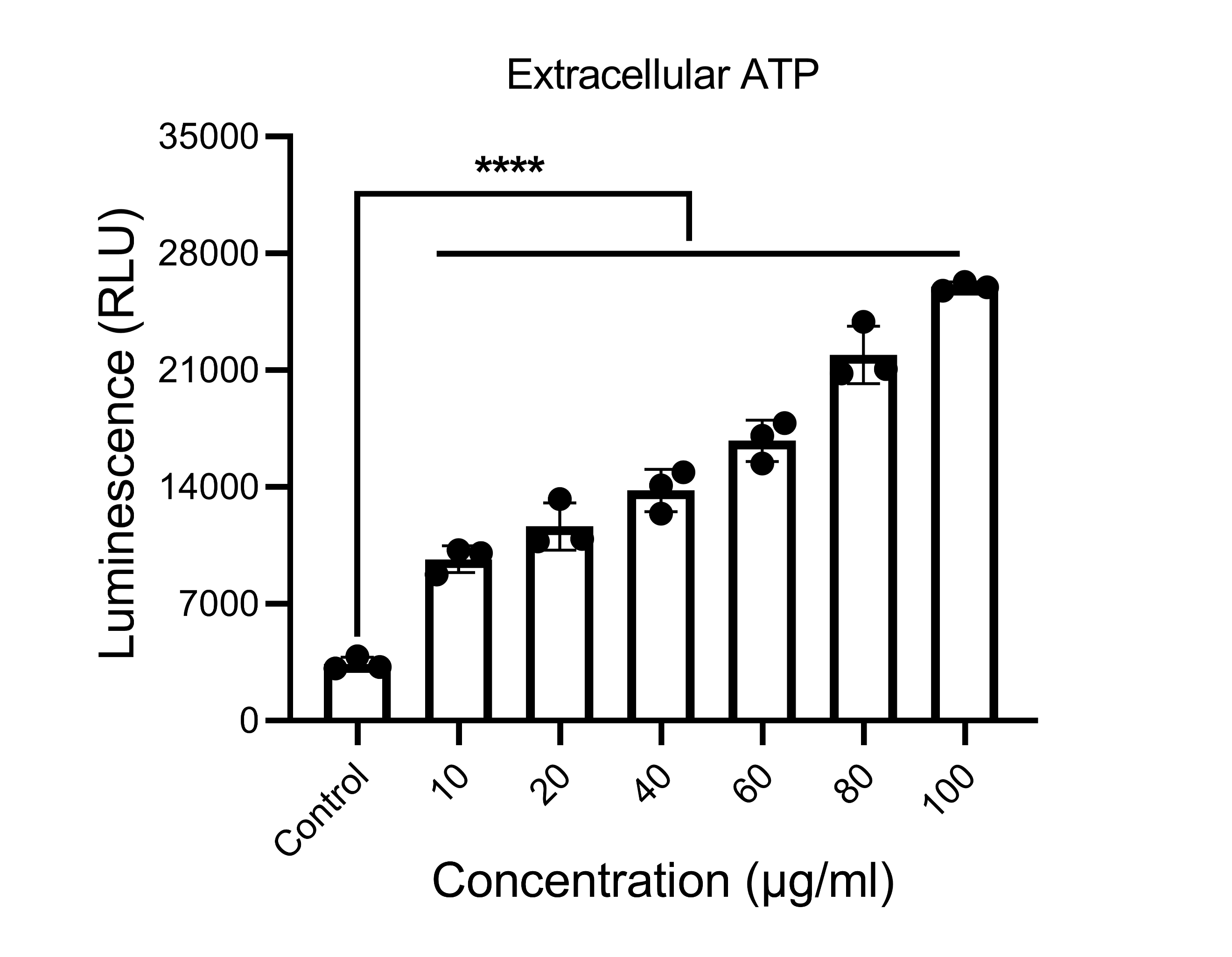


**Figure S5** Change level of extracellular ATP in CREC6 treated with different concentrations of BMAP-27B for 1 hour. Data are presented as mean values ± SD, n = 3 biologically independent samples. Data are expressed as mean values ± SD, based on three biologically independent samples. Statistical significance was analyzed by unpaired Student's t‐test (**p* < 0.05, ***p* < 0.01, ****p*< 0.001, *****p* < 0.0001).


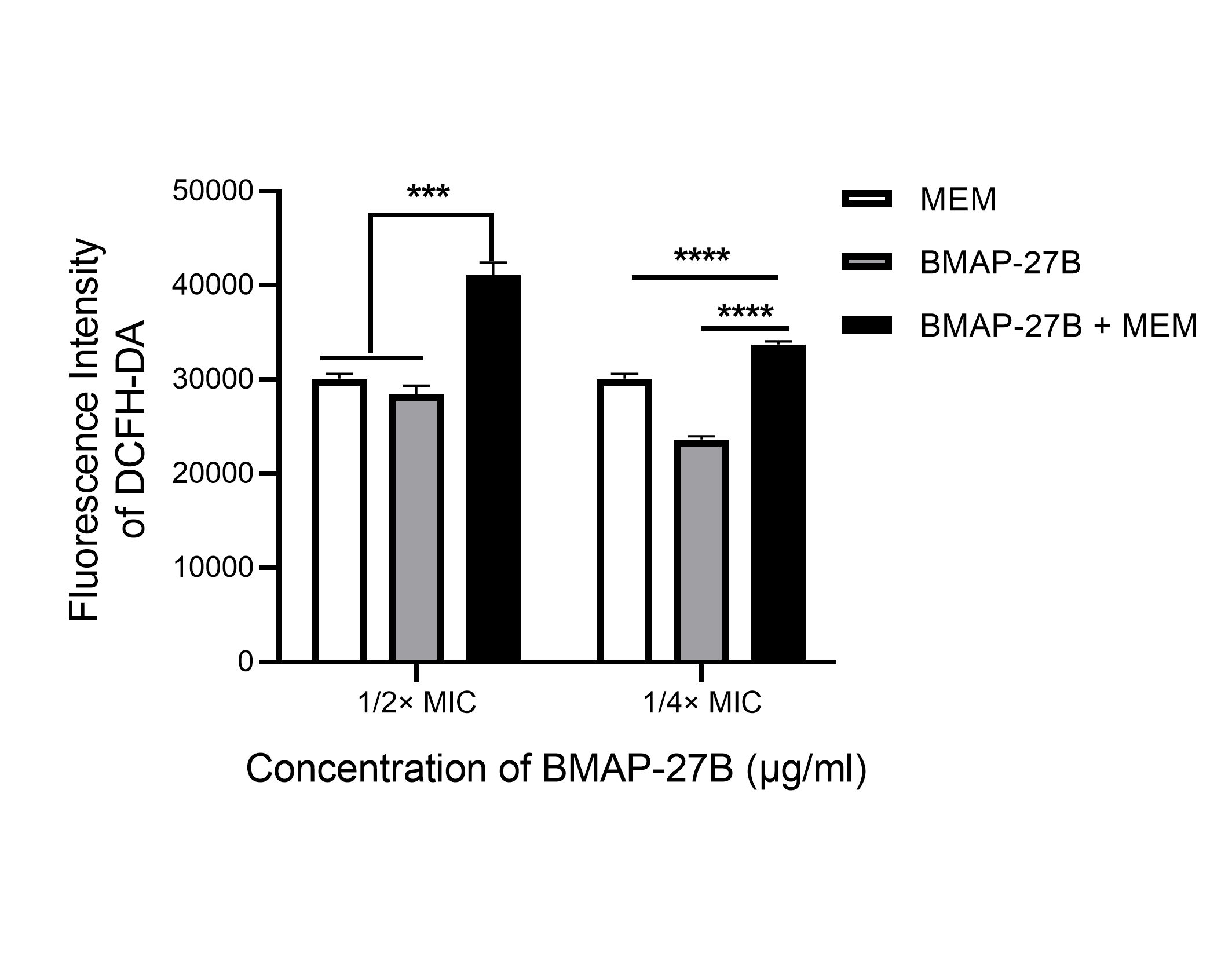


**Figure S6** ROS accumulation in CRKP2 treated with BMAP-27B (1/2× MIC or 1/4× MIC), meropenem (1/2× MIC), and their combinations for 30 minutes. Data are expressed as mean values ± SD, based on three biologically independent samples. Statistical significance was analyzed by unpaired Student's t‐test (**p* < 0.05, ***p* < 0.01, ****p*< 0.001, *****p* < 0.0001).


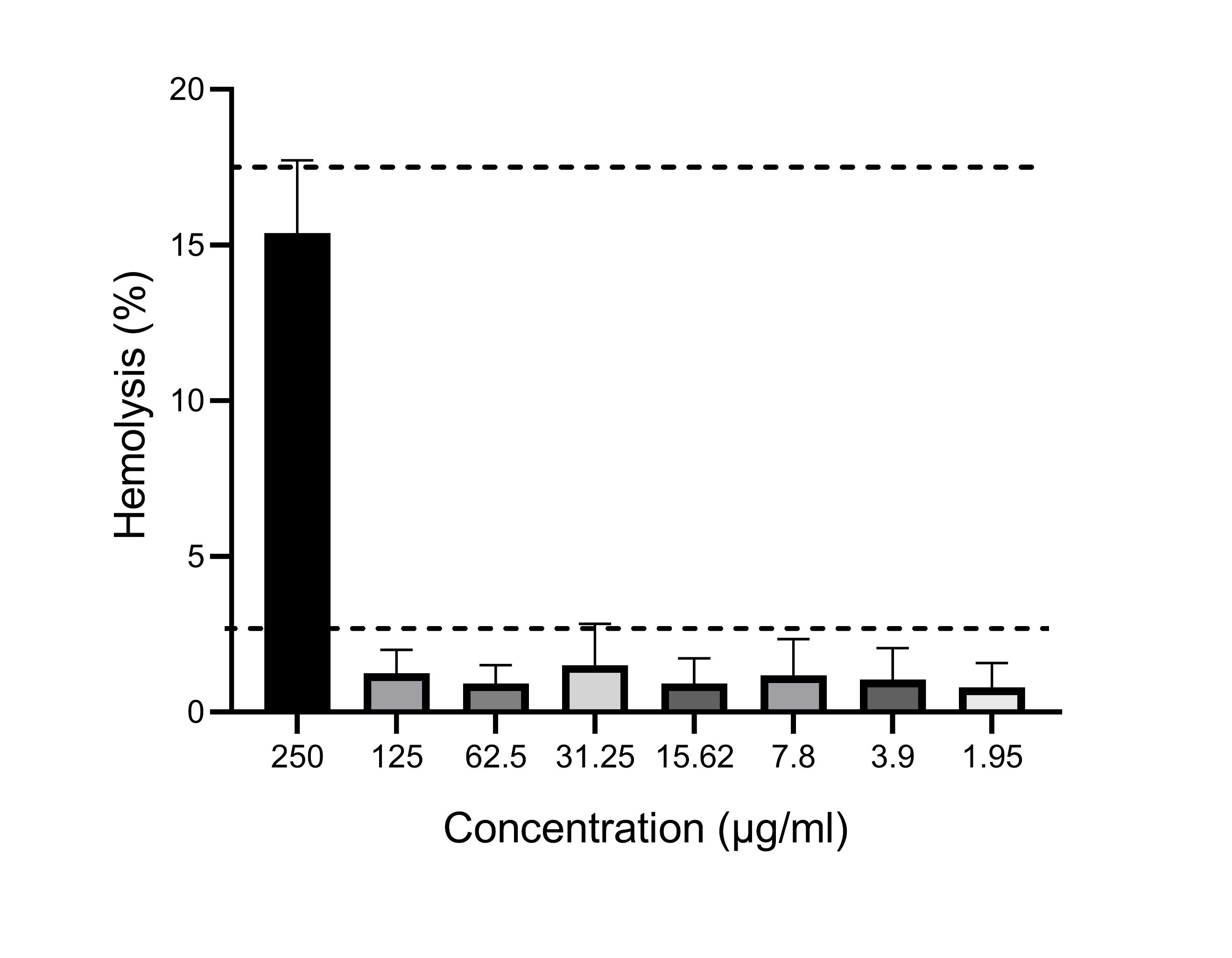
**Figure S7** Hemolytic activity of BMAP-27B. Data are presented as mean values ± SD, n = 3 biologically independent samples. Statistical significance was analyzed by unpaired Student's t‐test (**p* < 0.05, ***p* < 0.01, ****p*< 0.001, *****p* < 0.0001).


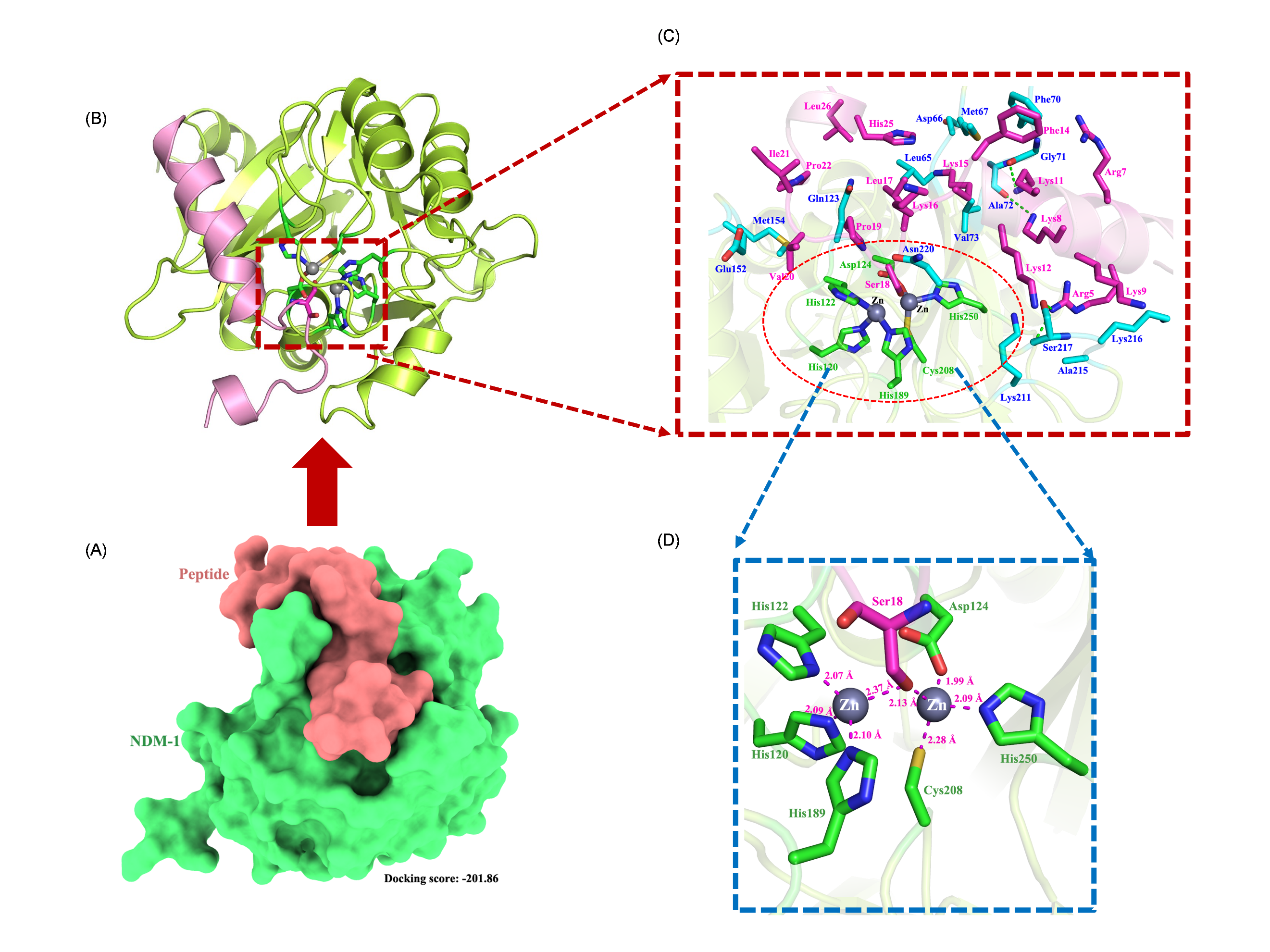


**Figure S8** **BMAP-27B disrupts the activity of NDM-1 by chelating zinc ions. (A)** Structural representation of the complex formed by docking NDM-1 with peptide molecules using the HDOCK program. **(B, C)** Interaction patterns between BMAP-27B and the NDM-1 protein. (The dotted green line shows hydrogen bonding). **(D)** Binding patterns between amino acid residues of peptide molecule and NDM-1 active site.

Supplementary 2: Tables

**Table S1** Interaction and distance between NDM-5 and BMAP-27B

| **NDM-5** | **Peptide** | **Interaction type** | **Distance(nm)** |
| --- | --- | --- | --- |
| Gly69 | Arg7 | Hydrogen bond | 0.27 |
| Gly71 | Lys11 | Hydrogen bond | 0.27 |
| Ala72 | Lys8 | Hydrogen bond | 0.27 |
| Gln123 | Pro19 | Hydrogen bond | 0.28 |
| Asp212 | Arg5 | Hydrogen bond | 0.28 |
| Asn220 | Lys12 | Hydrogen bond | 0.29 |
| Phe70 | Arg7 | Pi-Cation | 0.30 |
| Met67 | Leu17 | Alkyl hydrophobic | 0.49 |
| Val73 | Leu17 | Alkyl hydrophobic | 0.52 |
| Leu154 | Pro22 | Alkyl hydrophobic | 0.48 |
| Phe70 | Arg7 | Pi-Alkyl | 0.54 |
| Phe70 | Leu10 | Pi-Alkyl | 0.54 |
| Phe70 | Lys11 | Pi-Alkyl | 0.47 |
| Trp93 | Pro19 | Pi-Alkyl | 0.53 |
| His122 | Val20 | Pi-Alkyl | 0.48 |
| Leu65 | His25 | Pi-Alkyl | 0.49 |
| His122 | Pro19 | Amide-Pi stacked | 0.42 |
| Met67 | His25 | Pi-Sigma | 0.33 |

**Table S2** Interaction and distance between NDM-1 and BMAP-27B

| **NDM-1** | **Peptide** | **Interaction type** | **Distance(nm)** |
| --- | --- | --- | --- |
| Ser217 | Arg5 | Hydrogen bond | 0.31 |
| Ser217 | Arg5 | Hydrogen bond | 0.30 |
| Ala72 | Lys8 | Hydrogen bond | 0.33 |
| Gly71 | Lys11 | Hydrogen bond | 0.30 |
| Lys216 | Arg5 | Alkyl hydrophobic | 0.45 |
| Lys216 | Lys9 | Alkyl hydrophobic | 0.45 |
| Phe70 | Lys11 | Pi-Alkyl | 0.53 |
| Met67 | Lys15 | Alkyl hydrophobic | 0.46 |
| Val73 | Lys15 | Alkyl hydrophobic | 0.51 |
| His122 | Ser18 | Amide-Pi stacked | 0.34 |
| His122 | Pro19 | Pi-Alkyl | 0.50 |
| His122 | Val20 | Pi-Alkyl | 0.55 |
| Met154 | Pro22 | Alkyl hydrophobic | 0.46 |
| Leu65 | His25 | Pi-Sigma | 0.38 |

**Table S3** The primer sequence information of resistance genes

| Primer name | sequence | product size(bp) | Annealing temperature (℃) |
| --- | --- | --- | --- |
| CTX-M-1-F | CTTCCAGAATAAGGAATCCC | 949 | 55 |
| CTX-M-1-R | CGTCTAAGGCGATAAACAAA |  |  |
| CTX-M-9-F | TGACCGTATTGGGAGTTTG | 902 | 56 |
| CTX-M-9-R | ACCAGTTACAGCCCTTCG |  |  |
| CMY2-F | AACACACTGATTGCGTCTGA | 1228 | 62 |
| CMY2-R | TCCTGGGCCTCATCGTCAGTTAT |  |  |
| CTX-M-2F | CGACGCTAC CCCTGC TAT T | 552 | 58 |
| CTX-M-2R | CCAGCGTCAGATTTTTCAGG |  |  |
| CTX-M-8-F | AACACGCAGACGCTCTAC | 600 | 55 |
| CTX-M-8-R | TCGAGCCGGAAGGTGTCAT |  |  |
| TEM-F | AGGAAGAGTATGATTCAACA | 550 | 58 |
| TEM-R | CTCGTCGTTTGGTATGGC |  |  |
| rmtB-F | ACATCAACGATGCCCTCAC | 725 | 55 |
| rmtB-R | AAGTTCTGTTCCGATGGTC |  |  |
| floR-F | CTGAGGGTGTCGTCATCTAC | 673 | 59 |
| floR-R | GTCCCGACAATGCTGACTAT |  |  |
| cfr-F | TAAGAAGTAATAATGAGC | 518 | 48 |
| cfr-R | TATAGAAAGTCTACGAGG |  |  |
| tetA-F | CTGATCGTAATTCTGAGCACTG | 420 | 60 |
| tetA-R | CGCGACCATCCCGAACCCGAA |  |  |
| tetB-F | TTGGTTAGGGGCAAGTTTTG | 659 | 65 |
| tetB-R | GTAATGGGCCAATAACACCG |  |  |
| NDM-F | GAATTCGCCCCATATTTTTGC | 850 | 56 |
| NDM-R | AACGCCTCTGTCACATCGAAAT |  |  |
| fosA-F | CTCAACCATCTGACCCTCGC | 334 | 58 |
| fosA-R | CGTGCAGCTCCAGCTTGT |  |  |
| fosA3-F | GCGTCAAGCCTGGCATTT | 282 | 57 |
| fosA3-R | GCCGTCAGGGTCGAGAAA |  |  |
| aac(6’)-Ib-F | CAAGAGTCCGTCACTCCATACA | 396 | 61 |
| aac(6’)-Ib-R | ATGGAAGGGTTAGGCATCACT |  |  |
| qnrB-F | ATGACGCCATTACTGTATAA | 562 | 57 |
| qnrB-R | GATCGCAATGTGTGAAGTTT |  |  |
| qnrA-F | ATTTCTCACGCCAGGATTTG | 516 | 55 |
| qnrA-R | GATCGGCAAAGGTTAGGTCA |  |  |
| oqxA-F | GATCAGTCAGTGGGATAGTTT | 670 | 56 |
| oqxA-R | TACTCGGCGTTAACTGATTA |  |  |

**Supplementary Methods**

**Sample collection, Bacterial isolation and identification, detection of carbapenems resistance genes**

We collected samples (feces, anal, nasal and oral swabs of animals; sewage; soil) from pig and chicken farms in different cities of Guangxi. After the samples were processed, the carbapenem-resistant strains were screened out on MacConkey plates containing 4 mg/L meropenem. Subsequently, the presence of *bla*_NDM_ and other resistance genes, were determined by PCR using primers previously described. Primers are listed in Table S3.

**Multilocus sequence typing (MLST)**

The genetic correlation between the *bla*_NDM_-positive isolates was investigated by MLST. The MLST method and primers of *E. coli* were carried out according to the database (<http://mlst.warwick.ac.uk/mlst/dbs/Ecoli>), and the seven housekeeping genes were *adk*, *fum*, *gyrB*, *lcd*, *mdh*, *purA* and *recA*; The seven housekeeping genes (*rpoB*, *gapA*, *mdh*, *pgi*, *phoE*, *infB* and *tonB*) of *K. pneumoniae* were amplified by PCR, and the ST type were obtained on *K. pneumoniae* MLST database (<http://pubmlst.org/kpneumoniae>); MLST typing and identification of *Citrobacter bacillus* were performed as described in the literature (1). The seven housekeeping genes are *aspC*, *clpX*, *fadD*, *mdh*, *arcA*, *dnaG* and *lysP*, respectively.

**Antimicrobial activity of BMAP-27B in varying concentrations of proteases and serum**

BMAP-27B was incubated with different concentrations of proteases solution (protease K, pepsin and trypsin: 2000-5 μg/ml) and serum (50%-10%) at 1:1 (v/v) for 1h, and the incubation was diluted to 2×MIC with MHB medium. The above mixture was added to the bacterial suspension and incubated at 37℃ for 18-24 hours. Finally, the absorbance of OD_60_ was measured and used to calculate the survival rate of bacteria.

**Hemolysis Assays**

The defibrillated sheep red blood cells red blood cells with PBS (PH 7.4) and then resuspended in PBS at 1:100 (v/v). Add 50 μl of the red blood cell suspension into a 96-well plate containing different concentrations of BMAP-27B solution (50 μl) and incubated at 37 °C for 1 hour. Then the mixture was centrifuged and the absorbance of the supernatant at OD_540_ nm was measured. Positive and negative controls are prepared by adding 0.1% Triton X-100 and PBS to the red blood cell suspension, respectively. Hemolysis (%) = [sample absorbance–negative control absorbance) / (positive control absorbance–negative control absorbance)] × 100%.

**Live/dead fluorescence imaging assay of bacteria**

The MDR bacteria in the logarithmic growth phase were resuspended into PBS (10 mM, pH=5.0) and adjusted to OD_600_=0.3 while preparing PI in a 1 mg/ml solution with deionized water. Then, after the bacterial solution and 1×MIC peptide was incubated for 30 min at 37 °C, PI dye with a volume of 30 µl was added, and incubated at room temperature in the dark for 15min. The fluorescent images of stained bacteria were obtained by confocal laser scanning microscope (CLSM).

**Molecular docking assay**

To investigate the potential interaction between BMAP-27B and the NDM enzyme, molecular docking was performed using the HDCOK program. Crystal structures of NDM-1 (PDB ID: 5ZH1) and NDM-5 (PDB ID: 6MGY) were obtained in PDB format, and water molecules and other ligands were removed from these structures to serve as receptors for molecular docking. Using the BLAST method, crystal structure 2KET in the database was found to have a sequence similarity of 92.3% with the target sequence, and the three-dimensional structure of the peptide was constructed based on the crystal structure. Protein and peptide structure files were imported into the HDOCK software, and program parameters were set, including spatial interactions, hydrophobic interactions, hydrogen-bonding energy and rotatable keys numbers in the ligand. Docking scores were calculated based on the ITScorePP or ITScorePR iterative scoring functions. The more negative docking scores indicate potentially stronger binding and interaction in the model. And when the confidence score exceeds 0.7, it suggests a high probability of binding between the two molecules.

**Confidence score = 1.0/[1.0+e0.02*(Docking_Score+150)]**

1. Bai L, Xia S, Lan R, Liu L, Ye C, Wang Y, et al. Isolation and characterization of cytotoxic, aggregative Citrobacter freundii. PLoS One. 2012;7(3):e33054.
